# Prediction and reliability analysis of shear strength of RC deep beams

**DOI:** 10.1038/s41598-024-64386-w

**Published:** 2024-06-25

**Authors:** Khaled Megahed

**Affiliations:** Department of Structural Engineering, Mansoura University, PO Box 35516, Mansoura, Egypt

**Keywords:** Deep beams, Symbolic regression (SR), Support vector regression (SVR), XGBoost (XGB), CatBoost (CATB), Random forest (RF), Gaussian process regression (GPR), Artificial neural networks (ANN), Bayesian optimization (BO) technique, Reliability-based design, Civil engineering, Statistics, Scientific data, Computer science

## Abstract

This study explores machine learning (ML) capabilities for predicting the shear strength of reinforced concrete deep beams (RCDBs). For this purpose, eight typical machine-learning models, i.e., symbolic regression (SR), XGBoost (XGB), CatBoost (CATB), random forest (RF), LightGBM, support vector regression (SVR), artificial neural networks (ANN), and Gaussian process regression (GPR) models, are selected and compared based on a database of 840 samples with 14 input features. The hyperparameter tuning of the introduced ML models is performed using the Bayesian optimization (BO) technique. The comparison results show that the CatBoost model is the most reliable and accurate ML model (R^2^ = 0.997 and 0.947 in the training and testing sets, respectively). In addition, simple and practical design expressions for RCDBs have been proposed based on the SR model with a physical meaning and acceptable accuracy (an average prediction-to-test ratio of 0.935 and a standard deviation of 0.198). Meanwhile, the shear strength predicted by ML models was then compared with classical mechanics-driven shear models, including two prominent practice codes (i.e., ACI318, EC2) and two previous mechanical models, which indicated that the ML approach is highly reliable and accurate over conventional methods. In addition, a reliability-based design was conducted on two ML models, and their reliability results were compared with those of two code standards. The findings revealed that the ML models demonstrate higher reliability compared to code standards.

## Introduction

Reinforced concrete deep beams (RCDBs), characterised by a small span-to-height ratio (typically below 2.5)^1–3^, are commonly employed in various structures such as lower floors, transfer girders, and pile caps due to higher shear strength compared to slender beams. Despite their widespread application, the design of RCDBs poses challenges due to the nonlinear impact of different parameters on their shear behaviour. The primary failure mode of RCDBs is shear stress, often resulting in sudden and catastrophic collapses, introducing significant safety risks. Various shear strength models for RCDBs have been investigated, including those employing machine learning methods^2–11^, the strut-and-tie model^12–14^, the compression field method^15^, and finite element analysis^16^. However, traditional design methods, such as the strut-and-tie model (STM) or mechanism analysis, often fail to adequately capture the complex relationship between parameters affecting shear strength, leading to imprecise and conservative results compared to test results. Furthermore, the design provisions available, e.g., ACI 318^17^, and EC2^18^, and different models^13,14^, provide simple procedures for calculating the shear capacity of RCDBs but their conservative nature and their discrepancy with test results fail in introducing a comprehensive model that can approximate the shear capacity of RCDBs accurately.

In recent developments, new models have been proposed to enhance the prediction of shear capacity in deep beams. Chen et al.^19^ introduced the cracking STM model, which integrates the STM approach with considerations of diagonal crack patterns and strain distributions in horizontal reinforcement. Meanwhile, Chetchotisak et al.^20^ presented a modified interactive STM for RCDBs, relying on two distinct load-bearing mechanisms: the inclined strut and the truss. This model refines the strut mechanism from the interactive STM and incorporates empirical constants into the Mohr–Coulomb failure criterion to define a new concrete failure mode. Fan et al.^21^ proposed an STM for unsymmetrically loaded RC deep beams, where the geometry of the compression nodal zones is determined using Mohr's Circle and the minimum strain energy criteria. Despite aligning well with experimental data, these models require tedious calculations.

Machine learning (ML) has become a promising tool in many engineering aspects, providing an alternative procedure for addressing engineering challenges. ML algorithms, including support vector machines, artificial neural networks, genetic algorithms, and ensemble learning methods, have been extensively used in predicting the shear strength of RCDBs^2–11^. For example, Ma et al.^2^ implemented six ML models to predict the shear strength of RCDBs and compared their performance with five previous closed-form models. Recently, Nguyen et al.^3^ implemented seven machine learning models for predicting the shear strength of RCDBs and found that Gaussian process regression (GPR) is the most reliable and accurate ML model. Feng et al.^6^ studied four typical ensemble learning models, including random forests, gradient boosting regression tree, adoptive boosting and extreme gradient boosting (XGBoost), to predict the shear capacity of RCDBs using a dataset of 271 samples and grid search method for hyper-parameters tunning. The comparison results of these models showed that the XGBoost model is the best model concerning prediction accuracy (R^2^ = 0.992 and 0.917 in the training and testing sets, respectively). However, the metric errors in the testing set are nearly 3–8 times those in the training set, indicating signs of overfitting. Recently, Tiwari et al.^7^ used eight ML models for the shear capacity of RCDBs and found that the XGBoost model exhibited the highest accuracy. Ashour et al.^4^ used genetic expression programming to develop an empirical expression for the shear strength of RCDBs using 141 test data. Shahnewaz et al.^9^ and Wakjira^10^ used a genetic algorithm to predict the shear strength of RCDBs. Liang et al.^22^ devoloped a symbolic regression (SR) model based on the Modified Compression Field Theory to analyze the punching shear resistance of fiber-reinforced polymer (FRP) reinforced concrete slabs.

From literature review, it was found that limited researchers ^23,24^ have examined the safety of RC deep beams designed according to the code design practice. Aguilar et al.^23^ evaluated the reliability of deep beams designed using the strut-and-tie method according to ACI 318. They found that ACI 318 design practice increases the likelihood of nonductile failure and suggested reliability-based strength reduction factors of 0.65 for struts and 0.90 for ties. Muendacha et al.^24^ conducted a safety-based evaluation of shear design methods for RC deep beams using strut-and-tie models (STMs) in accordance with international concrete codes, considering variability in load actions and member resistances as random variables. Their findings indicated that deep beams made from normal-strength concrete and designed using these STMs provided a satisfactory safety level, and they suggested probability-based reduction factors to achieve a target reliability index greater than 3.5. Regarding the integration of reliability analysis with machine learning, Shen et al.^25^ combined reliability analysis with machine learning by using Monte Carlo simulation alongside a machine learning-based surrogate model to calibrate the reliability of slab-column joints for punching shear resistance.

It can be concluded from these studies that ML can be used successfully to predict the shear strength of RCDBs accurately. However, most models depend on primitive search algorithms, such as grid search techniques for tuning ML parameters, lacking sophistication in refining the ML models. Moreover, most recent studies lack a real-world practical application and fail to highlight the gap between the theory and practical implementation. While many ML models exhibit superior results, deriving an explicit design formula from these models is challenging. The black-box and difficult-to-interpret nature of these models hinders their practical implementation in engineering design. Moreover, previously introduced ML studies primarily focus on prediction outcomes and accuracy without engaging in reliability-based design to bridge the gap between ML and practical engineering applications. Furthermore, many studies develop separate models for specific beam cases, such as those with or without web reinforcements^2,10^. This approach not only lacks generalisation but also introduces fluctuations in the results. In addition, most expressions introduced through ML techniques, i.e., genetic expression programming (GEP) and genetic algorithm (GA), lack clear interpretation, lack physical meaning, and are overly complex^4,9,10^. Table 1 provides an overview of ML models and previous formulas employed in previous studies, as well as their associated results.

**Table 1:** Summary of previous ML models in predicting RCDBs shear strength.

Reference	Category (number)*	Models: Statistical criteria
Wakjira^10^	WOR (371)	**GP**: ${V}_{th}=0.0456{f}_{c}^{\prime 0.619}{\rho }_{l}^{0.411}{\left(\frac{a}{d}\right)}^{-0.874}{b}_{w}d$ μ = 0.82, COV = 0.305
Ashour^4^	All (141)	**GP:** $V={b}_{w}h\sqrt{{f}_{c}^{\prime}}\left[\left(-4.56+1.68\frac{a}{d}\right){\rho }_{l}^{2}+\left(2.45+0.1{\left(\frac{a}{d}\right)}^{2}-1.16\frac{a}{d}+3.12{\rho }_{t}\right){\rho }_{l}+0.3{\rho }_{hw}+0.4{\rho }_{vw}\right]$ μ = 1.11, Std = 0.21
Shahnewaz^9^	All (381)	GA: ${V}_{u}={b}_{w}h{f}_{c}^{\prime}\left[\frac{2}{5}-\frac{1}{4}{\left(\frac{a}{d}\right)}^{0.23}+0.85{\left({\rho }_{l}{\rho }_{hw}{\rho }_{vw}\right)}^{0.1}-\frac{3}{5}{\left(\left(\frac{a}{d}\right){\rho }_{hw}{\rho }_{vw}\right)}^\frac{1}{16}-200{\left(\left(\frac{a}{d}\right){\rho }_{l}{\rho }_{hw}{\rho }_{vw}\right)}^{2.65}\right]$ μ = 0.99, CoV = 0.232 GA: ${V}_{u}={b}_{w}h{f}_{c}^{\prime}\left[1.74-2{\left(\frac{a}{d}\right)}^{0.044}+0.5{\rho }^{0.14}\right]$ μ = 1.01, CoV = 0.257
Cheng^5^	All (106)	EMARS, BPNN, RBFNN, SVM. EMARS is the best model. Grid search with cross-validation **EMARS**: training MAPE = 5.67, R^2^ = 0.989, testing MAPE = 5.887, R^2^ = 0.973
Feng^6^	All (271)	DT, SVM, ANN, RF, AdaBoost, GBRT, XGBoost. XGboost is the best model. Grid search with cross-validation **XGboost:** Training R^2^ = 0.999, MAPE = 0.74, testing R^2^ = 0.928, MAPE = 10.44% (overfitting)
Hameed^11^	All (271)	LWR, RF, MLR, ELM. LWR is the best model. Grid search with cross-validation **LWR:** Training: RMSE = 22.563, MAE = 13.249, a20-index = 98.89, testing: RMSE = 57.776, MAE = 33.933, a20-index = 85.87
Liu^8^	All (267)	LR, SVR, ANN, RF, XGBoost, NGBoost using Bayesian optimisation technique. NGBoost is the best model **NGboost:** R^2^ = 0.9045, RMSE = 38.7976 kN
Tiwari^7^	All (271)	DT, SVR, RF, GB, Adaptive boosting, XGBoost, voting regression. XGboost is the best model. Grid search with cross-validation **XGboost:** Training R^2^ = 0.999, MAPE = 0.78, RMSE = 1.45 kN, testing R^2^ = 0.928, MAPE = 9.79, RMSE = 47.76 (overfitting). μ = 1.00, CoV = 6.38%
Nguyen^3^	All (518)	LR, ANN, SVR, DT, GPR, XGBoost using Bayesian optimisation technique. GPR is the best model **GPR:** Training R^2^ = 0.99, MAE = 12.77, RMSE = 18.84 kN, validation R^2^ = 0.89, MAE = 41.72, RMSE = 71.06 kN, testing R^2^ = 0.94, MAE = 38.44, RMSE = 63.38
Ma^2^	All (457)	kNN, DTM RFM GBDT, CatBoost, XGboost. XGboost is the best model. Grid search with cross-validation **XGboost:** Training R^2^ = 0.992, MAE = 0.148, RMSE = 0.26, testing R^2^ = 0.917, MAE = 0.531, RMSE = 0.777 WOR*: μ = 1.03, Std = 0.128, WVR*: μ = 1.005, Std = 0.073, WHR*: μ = 1.003, Std = 0.077, WVHR*: μ = 1.01, Std = 0.084
This study	All (840)	**CATBoost**: Training: μ = 1.005, CoV = 0.062, a20-index = 0.9894, MAPE = 4.41, RMSE = 36.8 kN Testing: μ = 1.026, CoV = 0.141, a20-index = 0.899, MAPE = 9.32, RMSE = 160.9 kN **SR**: (WOR)* μ = 1.003, CoV = 0.207, a20-index = 0.68, MAPE = 16.80, RMSE = 115.9 kN **SR**: (WWR)* μ = 1.004, CoV = 0.192, a20-index = 0.78, MAPE = 13.70, RMSE = 196.7 kN

*WOR and WWR stand for without web reinforcement and with web reinforcement cases.

The present study introduces novel contributions in several key aspects. Firstly, it develops unified ML-based models for RCDBs shear strength, combining both beam cases, i.e., with and without web reinforcements, in a unique predictive model. while many previous studies focused on predicting each type independently^2,10^. Furthermore, the ML results are compared with those of mechanics-driven models, including two prominent design codes (American code (ACI318)^17^ and European code (EC2)^18^) and two previous mechanic-based models^13,14^ to validate the performance of the developed ML models. Secondly, the Bayesian optimization (BO) technique is adopted for selecting the optimal hyperparameters for the introduced ML models. This approach differs from the conventional and less advanced searching techniques commonly found in literature, such as the grid search technique. Thirdly, simple and practical design expressions for RCDBs have been proposed based on the symbolic regression (SR) model. These expressions are simple and easy to interpret and demonstrate remarkable accuracy compared to previous closed-form models. Finally, a reliability-based design assessment is conducted on two different ML models and two code standards to evaluate the reliability of utilising ML models in practical design applications.

## Experimental database of RC deep beams

**Figure 1:**
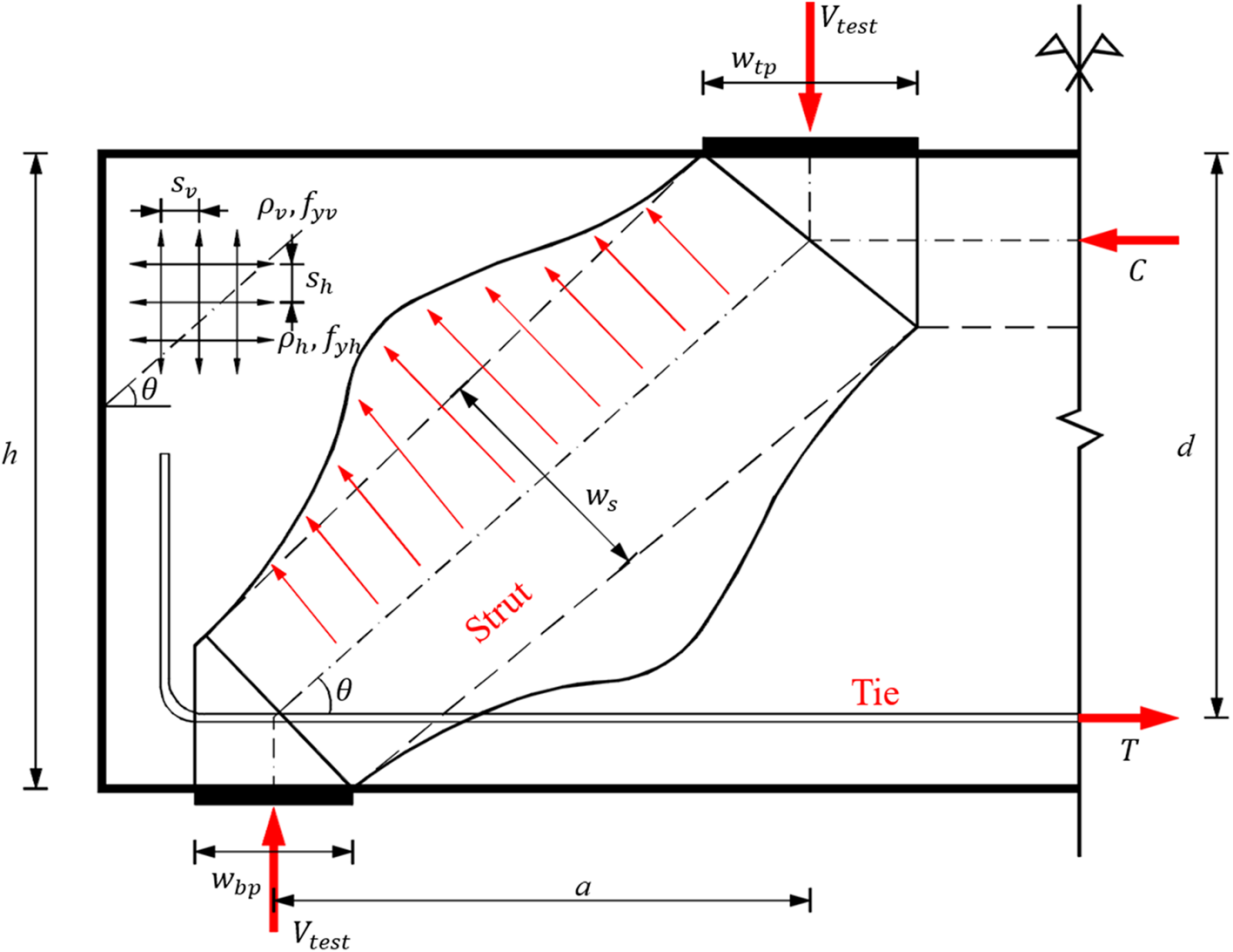
The dimensions of RC deep beam.

The schematic diagram of the shear mechanism of RCDBs is shown in Fig. 1. To construct robust ML models and investigate their influencing parameters, a dataset comprising 840 RCDB tests was collected in existing literature and from a database collected by Chetchotisak et al.^20^, including 322 specimens without web reinforcement (WOR) and 518 specimens with web reinforcement (WWR). The details of the collected database are provided in Supplementary data. Based on the results of various experimental and theoretical studies^12–14,26^, the shear capacity of RCDBs is influenced by different shear components, which typically encompass the strength of concrete material, longitudinal rebars and web reinforcement. Therefore, 14 different design features were set as the input variables, grouped into five categories^26^: (1) geometric dimensions: beam height (*h*), effective height (*d*), width (*b*_*w*_), shear span (*a*) and shear span-to-depth ratio (*a*/*d*); (2) concrete property, i.e., concrete strength (*f*_*c*_′); (3) bottom longitudinal reinforcement properties: reinforcement ratio (*ρ*_*l*_), and strength (*f*_*yl*_); (4) web reinforcement properties: vertical web reinforcement (VWR) ratio (*ρ*_*v*_) and strength (*f*_*yv*_), horizontal web reinforcement (HWR) ratio (*ρ*_*h*_) and strength (*f*_*yh*_); (5) top plate width (*w*_*tp*_) and bottom plate width (*w*_*bp*_). The corresponding output is the shear strength index of the RCDBs (*V*_*u*_/*b*_*w*_*h f*_*c*_′), denoted by *v*_*n*_, where *V*_*u*_ is the web shear capacity. Table 2 summarises statistical information for the output and 14 input features within the established database.

**Table 2:** Statistic features of the experimental dataset.

Variable	Symbol	Type	Statistics					
			Min	Max	Mean	Std	Skewness	Kurtosis
Beam height	$h$(mm)	Input	152	2100	564	291	2.076	5.407
Beam effective height	$d$(mm)	Input	137	2000	499	271	2.16	5.962
Beam width	${b}_{w}$(mm)	Input	51	914	196	120	2.601	9.865
Shear span	$a$(mm)	Input	80	4375	637	465	2.629	9.976
Shear span-to-depth ratio	$a/d$	Input	0.27	2.502	1.304	0.541	0.349	− 0.499
Concrete strength	${f}_{c}^{\prime}$(MPa)	Input	11.3	120.1	40.2	21.6	1.122	0.698
Bottom reinforcement ratio	${\rho }_{l}$	Input	0.003	0.113	0.02	0.011	1.704	7.3
Bottom reinforcement strength	${f}_{yl}$(MPa)	Input	267	1330	470	131	2.828	13.993
Vertical web reinforcement ratio	${\rho }_{v}$	Input	0	0.029	0.003	0.004	2.493	9.767
Vertical web reinforcement strength	${f}_{yv}$(MPa)	Input	0	1051	273	227	0.103	− 0.627
Horizontal web reinforcement ratio	${\rho }_{h}$	Input	0	0.032	0.002	0.003	3.553	17.956
Horizontal web reinforcement strength	${f}_{yh}$(MPa)	Input	0	855	206	230	0.426	− 1.329
Top plate width	${w}_{tp}$(mm)	Input	10	914	146	107	3.094	12.72
Bottom plate width	${w}_{bp}$(mm)	Input	10	610	136	82	2.534	8.657
Shear strength index	${v}_{n}=\frac{{V}_{u}}{{b}_{w}h{f}_{c}^{\prime}}$	Output	0.011	0.293	0.134	0.053	0.384	− 0.287

**Figure 2:**
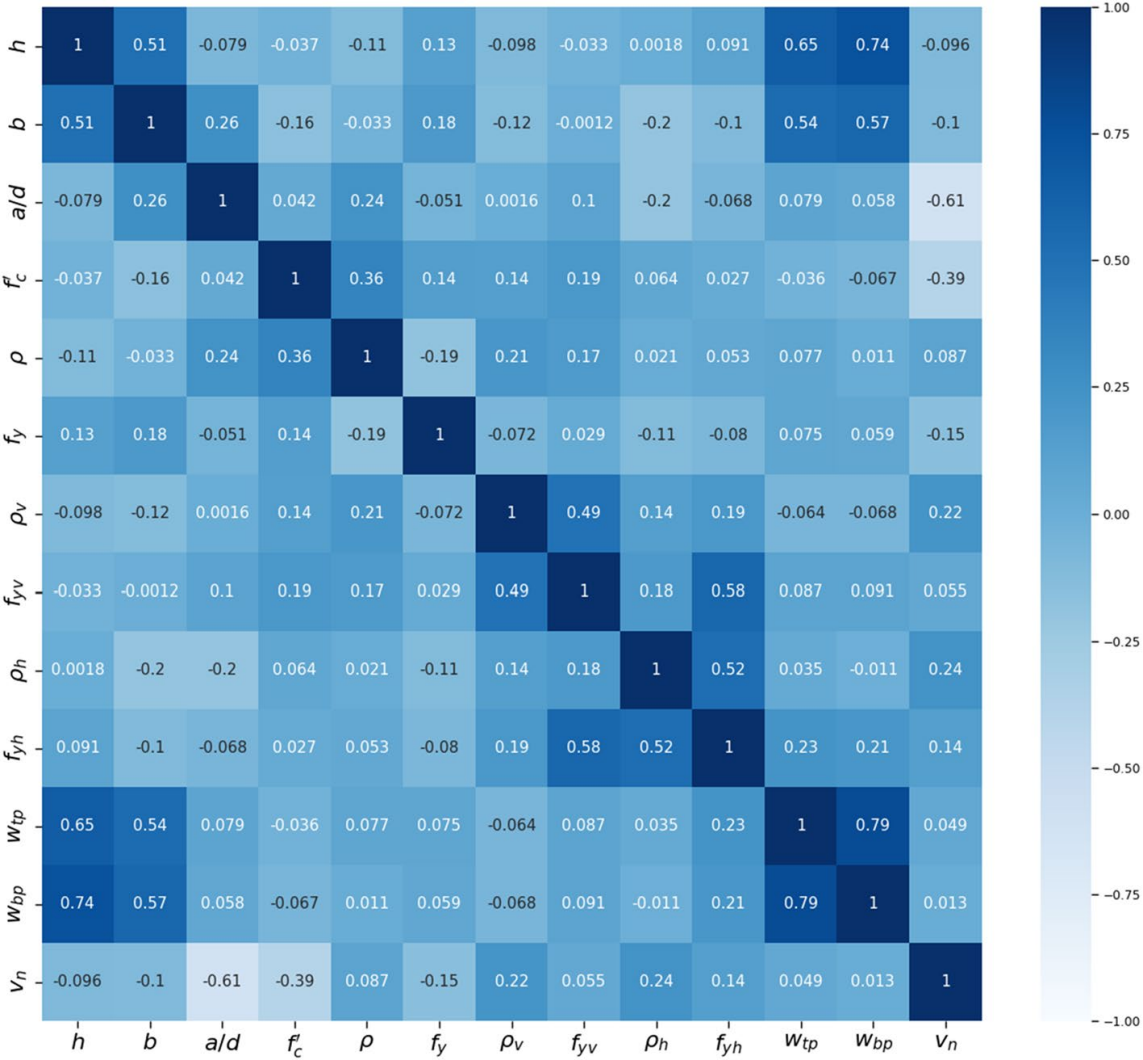
Correlation matrix for the RC deep beams database.

The Pearson correlation coefficient (r) is used in this study to assess the strength of the linear correlation between any two features^27^. Spanning from − 1.0 to 1.0, a value of − 1.0 indicates a strong negative relationship, 1.0 signifies a strong positive relationship, and 0 denotes no correlation. As illustrated in Fig. 2, the Pearson correlation matrix displays that the relationship between most input features is insignificant. However, a relatively high degree of correlation is observed between the VWR/HWR ratios and VWR/HWR strengths and between the widths of the upper and lower bearing plates. The former correlation is attributed to the presence of 322 specimens without reinforcement (*ρ*_*v*_ = *f*_*yv*_ = *ρ*_*h*_ = *f*_*yh*_ = 0) out of the total 840, leading to a pseudo correlation effect. While the latter correlation between the widths of the upper and lower bearing plates arises from the fact that a significant portion of the tests were conducted with identical plate widths. Among all the input variables, the ratio *a*/*d*, concrete strength *f*_*c*_′, HWR ratio *ρ*_*h*_*,* and VWR ratio *ρ*_*v*_ appear to have the most significant impact on the shear strength index (*V*_*u*_/*b*_*w*_*h f*_*c*_′), with correlation values of − 0.91, − 0.39, 0.24, and 0.22, respectively. These findings imply that increasing the ratio *a*/*d* will significantly reduce the shear strength index. Similarly, increasing concrete strength triggers the brittle failure of the beam, leading to a reduction in the strength index, while increasing VWR/HWR ratios enhances the ductility of the RCDBs. These observations align well with the mechanical behaviour and experimental results of RCDBs^12–14,26^.

### Research significance

This study presents novel contributions in multiple domains: Firstly, it introduces unified machine learning models for predicting shear strength in Reinforced Concrete Deep Beams (RCDBs). Additionally, the study employs Bayesian Optimization for hyperparameter tuning. Simple and practical design expressions based on symbolic regression are proposed, demonstrating remarkable accuracy compared to previous mechanism models. In addition, a reliability-based design assessment evaluates the reliability of using machine learning models in practical design applications.

## ML algorithms

In this study, eight typical ML models are selected to predict the shear strength of RCDBs, including symbolic regression (SR)^28,29^, Gaussian process (GPR)^30^, artificial neural network (ANN), light gradient-boosting machine (LightGBM)^31^, random forests (RF)^32^, categorical boosting (CatBoost)^33^, extreme gradient boosting (XGBoost)^34^, and Support vector regression (SVR)^35^. The predictive performances of these models are then evaluated and compared. In general, ensemble learning tends to exhibit higher accuracy and stability compared to individual models^2,6–8^.

Random forests, proposed by Breiman^32^, falls under the category of ensemble learning based on bagging, which utilises bagging sampling to create a subset for training weak learners (such as decision trees) and makes decisions on regression or classification tasks through averaging or voting. Several crucial parameters, including the number of trees, the maximum number of features, and the maximum depth of trees, significantly impact the training results. On the other hand, CatBoost, LightGBM, and XGBoost are all part of ensemble learning based on boosting, which combines weak learners into a strong one through an iterative process^36^. CatBoost excels in handling categorical features, eliminating the need for preprocessing non-numerical features^33^. It solves the problem of gradient bias and enhances the generalization ability by employing unbiased boosting techniques with categorical features. LightGBM^31^ uses a histogram-based approach for splitting, while XGBoost^27^ utilises a level-wise depth-first approach, which results in faster training times and better handling of large databases with LightGBM compared to XGBoost. In subsequent sections, this paper will introduce two innovative ML models, including CatBoost and symbolic regression models.

## CatBoost model

CatBoost is a gradient boosting algorithm^33,37^, which differs from other gradient boosting algorithms in its use of ordered boosting, an efficient modification of gradient boosting algorithms. This modification can handle the problem of target leakage and can reduce prediction shift during training^33^. It is beneficial for small datasets, and it can handle categorical features. Specifically, the original variable is replaced with a new binary feature for each category. Another advantage of CatBoost is its use of random permutations in estimating leaf values during the selection of the tree structure^33^. This strategy helps overcome overfitting issues commonly associated with traditional gradient-boosting algorithms. Furthermore, CatBoost utilises binary decision trees as the foundational predictor.

**Figure 3:**
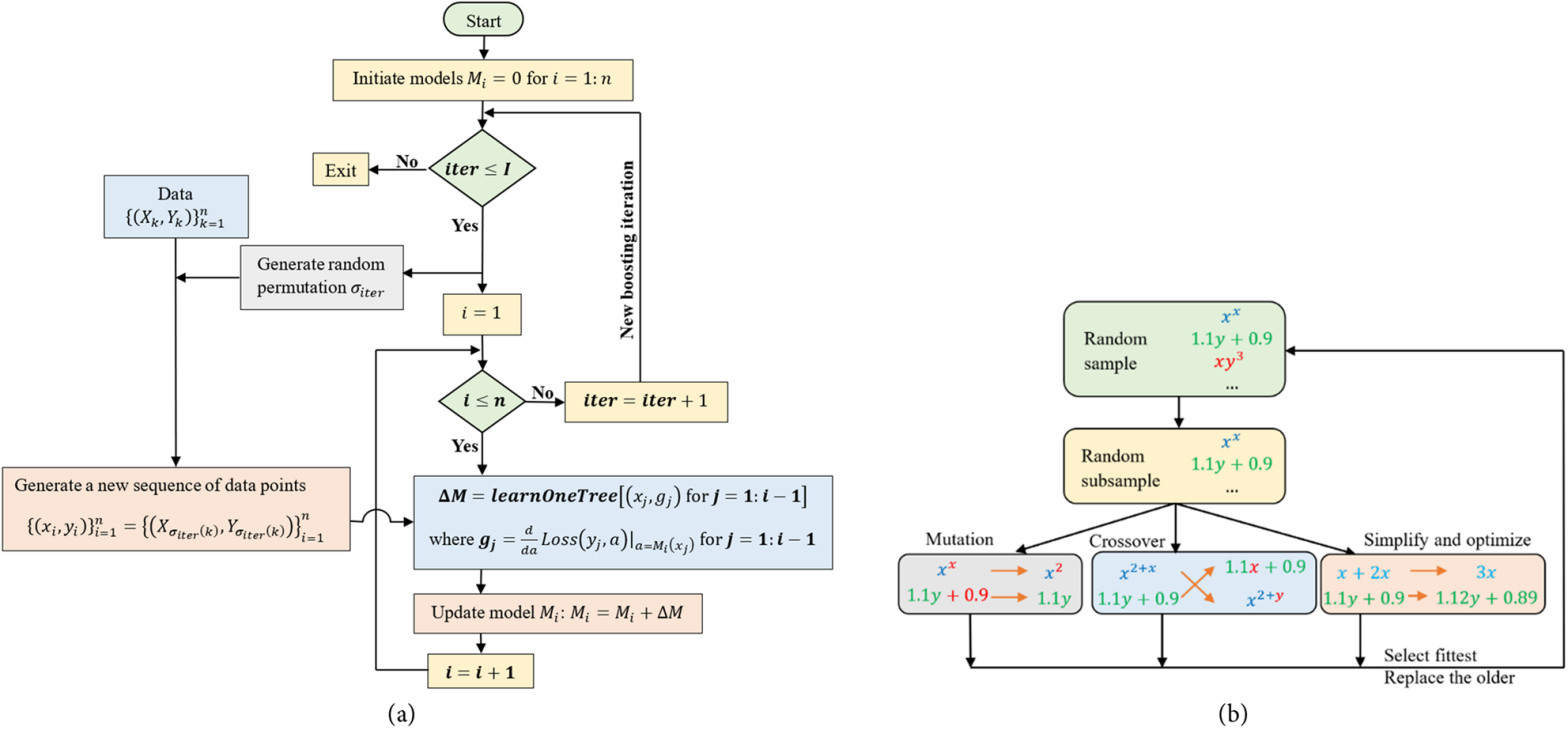
Flow charts of the introduced ML models. (**a**) CatBoost, (**b**) Symbolic regression.

As described by Dorogush et al.^33^, CatBoost can be outlined as follows: Let *T*_*i*_ represent the model built after constructing first *i* trees, ${g}_{i}\left({X}_{k}, {Y}_{k}\right)$ denote the gradient value on *k*-th training sample after constructing *i* trees. To ensure an unbiased gradient concerning the model *T*_*i*_, it is essential to train *T*_*i*_ without the observation *X*_*k*_. The standard training process appears impossible without observations since unbiased gradients are required for all training examples. The following trick is considered to handle this problem: for each example, *X*_*k*_, a separate model *M*_*k*_ is trained and never updated using a gradient estimate for that specific example. With *M*_*k*_, the gradient on *X*_*k*_ is estimated and used to score the resulting tree. Let us present the flowchart shown in Fig. 3a that explains how this trick can be performed. Let *Loss*(*y, a*) be the optimising loss function, where *y* is the label value and *a* is the formula value.

## Symbolic regression and proposed equations

Symbolic regression (SR)^28,29^ is a genetic programming technique^38^ which seeks to search simple and interpretable analytic formulas providing the best fit for a given model by exploring a predefined space of mathematical expressions and functions. SR are treated as multi-objective optimisation problems, finding a balance between the model's predictive accuracy and complexity. The genetic programming techniques are often utilised in SR by applying natural selection and evolution principles to iteratively refine candidate mathematical expressions until satisfactory models are obtained. This paper uses a Python library named PySR^32^ to search interpretable simple expressions for the shear capacity of the RCDBs.

**Figure 4:**
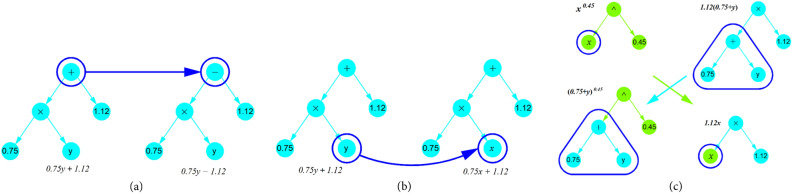
Mutation and crossover operations in SR model. (**a**) A mutation operation on expression tree, (**b**) a mutation operation on input variable, (**c**) a crossover operation between two trees.

The SR algorithm initiates by constructing an initial population with a random combination of operational symbols or functions (e.g., +, −, /, *, ^, etc.) and terminals, including input variables and constants. This process generates a tree-like expression for each individual in the population. Individuals are probabilistically selected, giving preference to the best-performing ones. The selected individuals undergo mutation (Fig. 4a,b) or crossover (Fig. 4c) to produce a new generation of populations, using a fitness function to identify the best individuals in each population generation is defined as^39^

1 $$l\left(E\right)={l}_{pred}\left(E\right).\text{exp}\left(\text{frecency}\left[C\left(E\right)\right]\right)$$

where ${l}_{pred}\left(E\right)$ is the prediction loss (selected as the mean absolute error), $C\left(E\right)$ is the complexity of the expression *E*, (the total number of nodes in the expression), and frecency [*C*(*E*)] measures the frequency and recency of the expression occurring at complexity *C*(*E*) in the population. This measure is employed to prevent excessive growth and redundancies in expressions generated, balancing error minimisation and simplicity. Table 3 outlines the SR parameters used in expression generation. The core steps of SR are presented in Fig. 3b.

**Table 3:** The parameters of the SR model used in generating expressions.

Parameters	Value	Parameters	Value
Number of generations	200	Allowed Binary operators	+, *, ^, /
Total number of populations	50	Loss function	Mean absolute error
Population size	20	Constraints	{‘^’:(–1,10)}^(a)^
Maximum length of expressions (total number of nodes)	30 (WWR), 22 (WOR)	Nested constraints	‘^’:{‘^’:0,’/’:1}, ‘/’:{‘/’:0,’^’:1}^(b)^
Parsimony (factor control the expression complexity)	0.02	Model_selection	Accuracy

(a) The constraint ‘^’:(− 1, 10) says that power laws can have any complexity in the left argument, but only 10 complexity (nodes) in the right argument.

(b) The nested constraints specify how many times a combination of operators can be nested. The constraint ‘/’:{‘/’:0,‘^’:1} indicates that ‘/’ may never appear within ‘/’, but ‘^’ can be nested once in ‘/’.

Selecting the optimal expression requires numerous iterations and a thorough investigation for each iteration. These iterations encompass trying various custom functions, a diverse set of operators, and extensive combinations of input features, which could potentially affect the shear strength of RCDBs^40^. The parameter selection process includes the most significant features identified from the Pearson correlation matrix, such as span-to-depth ratio (*a*/*d*), concrete strength (*f*_*c*_′), and reinforcement ratios (*ρ*_*l*_, *ρ*_*v*_, *ρ*_*h*_). Additionally, parameters from previous equations are considered, as outlined in Table 6, such as web reinforcement contribution (*ρ*_*v*_*f*_*yv*_, *ρ*_*h*_*f*_*yh*_) and the angle between the strut and the longitudinal axis (*θ*). The author also introduced some unitless parameters, including vertical and horizontal web reinforcement contribution factors (*ρ*_*v*_*f*_*yv*_/*f*_*c*_′, *ρ*_*h*_*f*_*yh*_/*f*_*c*_′) and the shear strength index (*V*_*u*_/*b*_*w*_*h f*_*c*_′). The SR algorithm generates different expressions for each iteration using various combinations of these parameters. Each resulting equation extracted with each iteration undergoes exhaustive evaluation and refinement. The selection criteria carefully weigh multiple factors, including equation complexity, accuracy, and interpretability. For RCDBs without web shear reinforcement, the following equation is derived:

2 $$\frac{{V}_{n}}{{b}_{w}h}=1.5{f}_{c}^{\left(0.85-0.22\frac{a}{d}\right)}{\rho }_{l}^{\left(0.29-\frac{a}{d}{\rho }_{l}\right)}, \quad \frac{a}{d}\le 2.5, \; \, {\rho }_{l}\le 0.1$$

For RCDBs with web shear reinforcement, the following equation is extracted:

3 $$\frac{{V}_{n}}{{b}_{w}h}=\frac{29{\rho }_{l}+3.8{\rho }_{l}^{0.3} \sqrt{{f}_{c}^{\prime}}}{{\left(0.3\right)}^{{\Psi }_{vh}}\left(\frac{a}{d}\right)+0.47}, \; {\Psi }_{vh}={\rho }_{h}\left(\frac{{f}_{yh}}{{f}_{c}^{\prime}}\right)+{\rho }_{v}\left(\frac{{f}_{yv}}{{f}_{c}^{\prime}}\right)$$

where ${\Psi }_{vh}$ represents the web shear reinforcement contribution factor. The proposed equations establish a comprehensive and simple framework for predicting the shear strength of RCDBs with meaningful physical interpretations. In the context of the RCDBs without web shear reinforcement in Eq. (2), it is evident that increasing the longitudinal reinforcement ratio ${\rho }_{l}$ or the concrete compressive strength will enhance shear capacity while increasing the shear span-to-depth ratio will weaken the beam shear strength. Notably, these findings align well with the conclusions drawn in the study of Ashour's study^4^, which identified the *a*/*d* ratio and *ρ*_*l*_ as the most significant parameters influencing shear behavior. Concerning RCDBs with web shear reinforcement in Eq. (3), the shear strength of RCDBs increase with increasing concrete strength, longitudinal reinforcement ratio *ρ*_*l*_, web shear reinforcement contribution ${\Psi }_{vh}$ and decreasing a/d ratio. These observations align well with the mechanical behaviour and experimental results of RCDBs^12–14,26,41^. Furthermore, the developed expressions are simple, robust, and have physical meaning compared to that of GEP and GA models introduced in the previous studies in Table 1.

## Data preprocessing and hyperparameter Bayesian optimisation technique

In this study, the min–max scaling technique is utilised for data normalisation to mitigate the adverse effects of multidimensionality. Following normalisation, the datasets are partitioned into two subsets for training and testing. Eighty percent of the original dataset is randomly allocated for training, while the remaining 20% is reserved for testing.

The performance of most ML algorithms relies heavily on their hyperparameters, which are predefined before model training. Properly tuning these hyperparameters is essential to ensure optimal prediction performance. Finding the best hyperparameters requires trying various sets of hyperparameters and selecting the parameter combination that yields the best performance with the validation data. Traditional techniques such as grid search (GS) and random search (RS) can be exhaustive and time-consuming, especially for models with various hyperparameters and large search space. In contrast, Bayesian optimization (BO) models utilise surrogate functions, i.e., Gaussian processes and tree-structured Parzen estimators (TPE)^34^, which guide the next selection of the hyperparameter combination depending on the performance of the previous history of tested hyperparameter values. This strategy minimises redundant evaluations, enabling BO to reach the optimal hyperparameter combination in fewer iterations compared to GS and RS methods^42^. This study adopted the TPE model^34^ to optimise the introduced ML models due to its superior robustness compared to other surrogate functions^42^. Mean Absolute Percentage Error, MAPE is chosen as the objective function in the validation dataset. The expected improvement (EI) of TPE, defined in Eq. (4), builds a probability model of the objective function and uses it to select the most promising hyperparameters to evaluate in the true objective function^43^:

4 $$E{I}_{{s}^{*}}\left(z\right)=\frac{\text{constant w}.\text{r}.\text{t }\left(z\right)}{\gamma +\left(1-\gamma \right)\frac{g\left(z\right)}{l\left(z\right)}}$$

where z is the hyperparameter combination chosen from the search space and s^*^ is a threshold chosen to be some quantile γ of the observed s values, so that $p\left(s<{s}^{*}\right)=\gamma$. Additionally, $l\left(z\right)$ and $g\left(z\right)$ correspond to two distinct distributions: one where the objective function values are below the threshold, l(z), and another where the values exceed the threshold, g(z). To maximize EI, TPE focuses on drawing samples of hyperparameters with the maximum l(z)/g(z) ratios from Eq. (4). Finally, cross-validation was applied to assess the introduced models' effectiveness, avoid overfitting, and obtain accurate predictions for the testing data. Table 4 presents the optimal hyperparameters for the introduced ML models.

**Table 4:** The optimal hyperparameters for ML models.

ML model	Optimal hyperparameters
CatBoost	iterations = 1696, learning_rate = 0.0906, depth = 4, subsample = 0.389, colsample_bylevel = 0.784, min_data_in_leaf = 10
GPR	Kernel: Constant*RBF + Constant*Matern + Constant*WhiteKernel + Constant* RationalQuadratic, gpr.alpha = 0.002
LightGBM	n_estimators = 1972, learning_rate = 0.0329, max_depth = 50, num_leaves = 10, boosting_type = Gradient Boosting Decision Tree
XGBoost	n_estimators = 1968, max_depth = 41, learning_rate = 0.0611, booster = ‘dart’, gamma = 0.01
RandomForest	random_state = 1000, n_estimators = 1134, max_depth = 22, min_samples_leaf = 2, max_features = ‘log2’, bootstrap = False
ANN	number of hidden layers = 1, neurons number of hidden layer = 12
SVR	Log(C) = 0.9988, log(epsilon) = − 74.88644548, log(gamma) = − 1.72300541

## Performance and results of ML models

**Figure 5:**
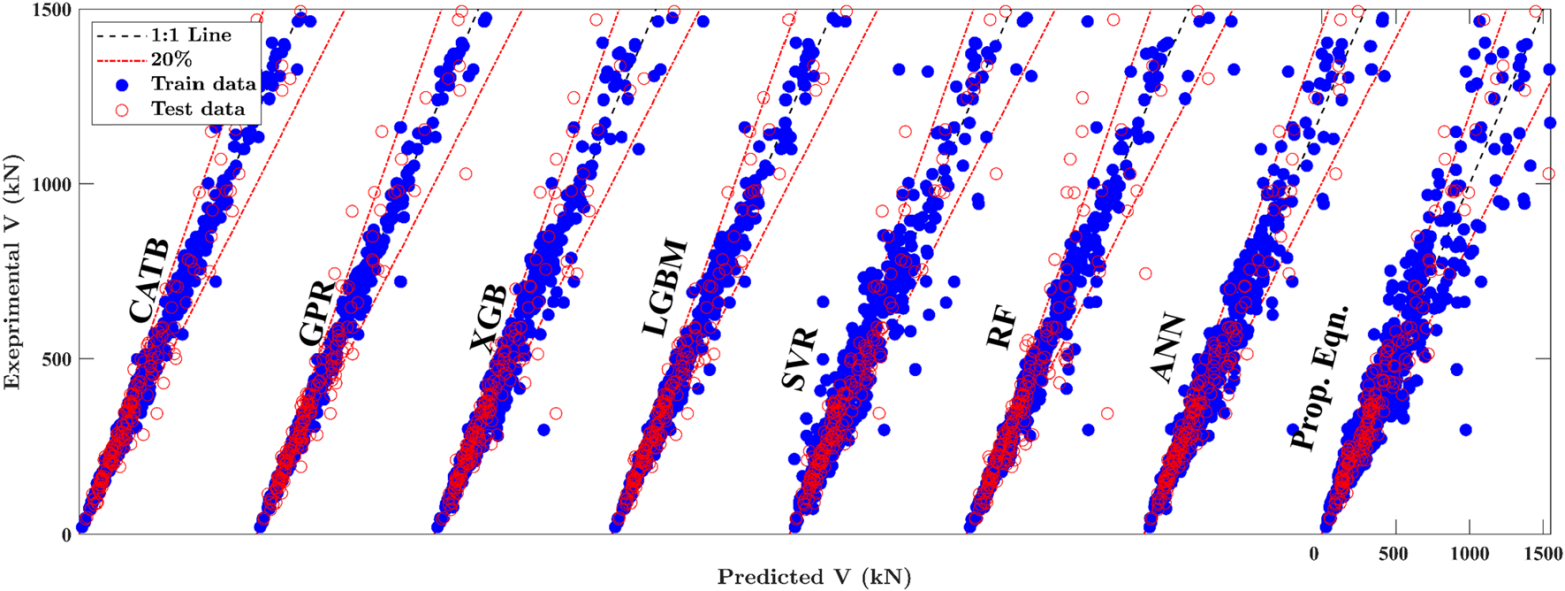
Comparison between proposed equations and ML models for training and testing datasets.

In this section, a comparison of the performance of the developed ML models is made. The details of established ML models are provided in Supplementary data, including hyperparameter tuning and results. In Fig. 5, the scatter plots depict the relationship between experimental and predicted results across different ML models. As noticed, the data points cluster closely around the diagonal line for most of the developed ML models, indicating a strong alignment between model expectations and test results. This alignment emphasises the reliability and prediction accuracy achieved by the developed models. Table 5 highlights evolution metrics used to study the performance of the implemented models, i.e., coefficient of determination (*R*^2^), the mean (*μ*), coefficient of variance (CoV), mean absolute percentage error (MAPE), root mean squared error (RMSE), and a20-index, defined as follows:

5 $${R}^{2}=1-\frac{\sum_{i=1}^{n}{\left({\widehat{y}}_{i}-{y}_{i}\right)}^{2}}{\sum_{i=1}^{n}{\left({y}_{i}-\overline{y }\right)}^{2}}, \; \mu =\frac{1}{n}\sum_{i=1}^{n}\frac{{\widehat{y}}_{i}}{{y}_{i}},\; MAPE=\frac{100\%}{n}\sum_{i=1}^{n}\left|\frac{{\widehat{y}}_{i}}{{y}_{i}}-1\right|,\; RMSE=\sqrt{\frac{1}{n}\sum_{i=1}^{n}{\left({\widehat{y}}_{i}-{y}_{i}\right)}^{2}}$$

where ${\widehat{y}}_{i}$ and ${y}_{i}$ are the predictions and actual output values of the *i-*th specimen, respectively, $\overline{y }$ is the mean value of actual observations, and *n* is the number of samples in the database. The a20-index^44^ introduces the ratio of specimens ${\widehat{y}}_{i}/{y}_{i}$ ratio within the interval of 0.80–1.20.

**Table 5:** Comparison of the developed ML models.

Metrics	Training data			Testing data			All data				
	CatB	GPR	LGBM	CatB	GPR	LGBM	CatB	GPR	LGBM	Prop.Eqn (WOR)	Prop.Eqn (WWR)
Mean $\mu$	1.005	1.006	1.008	1.026	1.023	1.026	1.01	1.01	1.012	1.003	1.004
CoV	0.062	0.062	0.076	0.141	0.151	0.154	0.085	0.088	0.098	0.207	0.192
R^2^	0.997	0.997	0.991	0.933	0.947	0.875	0.986	0.988	0.971	0.917	0.937
MAPE	4.41	4.44	5.32	9.32	10.27	9.61	5.39	5.60	6.18	16.80	13.70
RMSE( kN)	36.8	39.6	63.6	160.9	143.3	219.9	79.1	73.2	113.6	115.9	196.7
a20-index	0.994	0.993	0.981	0.899	0.857	0.887	0.975	0.965	0.962	0.68	0.78

As shown in Table 6, all introduced ML models display mean *μ*, *R*^*2*^, and a20-index values close to 1.0 and small values for CoV, MAPE, and RMSE. The MAPE values for the CATB model are 4.41 and 9.32 in the training and testing sets, respectively, which reach the lowest values compared to other models. Similarly, those of the GPR model are 4.94 and 10.27, and those of the LGBM model are 5.32 and 9.61, indicating the high accuracy of the developed models. The CoV and MAPE for all ML models are nearly twice as high for the testing data compared to the training data, indicating consistent training with minimal overfitting tendencies. Furthermore, the *μ* values of the CATB model are 1.005 and 1.026, the *R*^2^ values are 0.997 and 0.933, and the a20-index values are 0.994 and 0.899 in the training and testing sets, respectively, which are all close to 1.00. Such evaluation metrics reveal that the CATB model introduces the best prediction accuracy and predictive balance between the training and testing sets.

**Table 6:** Summary of previous mechanical models in predicting RCDBs shear strength. MW stands for Matamoros and Wong's formula.

Code Standard	Formulas
ACI318^17^	${V}_{ACI}=0.85{\beta }_{s}{f}_{c}^{\prime}{b}_{w}{w}_{s}\text{sin}\theta ,{w}_{s}=\left[1.8{w}_{t}*\text{cos}\theta +\left({w}_{tp}+{w}_{bp}\right)\text{sin}\theta \right]/2$
EC2^18^	${V}_{u,EU}=0.85{\beta }_{s}*{f}_{c}^{\prime}{b}_{w}{w}_{s}\text{sin}\theta ,{w}_{s}=[1.85{w}_{t}*\text{cos}\theta +\left({w}_{tp}+{w}_{bp}\right)\text{sin}\theta ]/2$
Matamoros and Wong^13^	${V}_{M-W}={C}_{c}{f}_{c}^{\prime} {b}_{w}{w}_{s}+{C}_{wv}{\rho }_{v}{b}_{w}\frac{a}{3}{f}_{yv} + {C}_{wh}{\rho }_{h}{b}_{w}\frac{d}{3}{f}_{yh},$ ${C}_{c}=0.3/\left(\frac{a}{d}\right)$, ${C}_{wv}=1$,${C}_{wh}=3(1 -a/d)$
Russo et al.^14^	${V}_{Russo}=0.76\left(k\chi {f}_{c}^{\prime}\text{sin}\theta +0.35\frac{a}{d}{\rho }_{v}{f}_{yv}+0.25\text{tan}\theta {\rho }_{h}{f}_{yh}\right){b}_{w}d, \chi =0.74{r}^{3}-1.28{r}^{2}+0.22r+0.87, r={f}_{c}^{\prime}/105$
Current study	Without web reinf.: $\frac{{V}_{n}}{{b}_{w}h}=1.5{f}_{c}^{\left(0.85-0.22\frac{a}{d}\right)}{\rho }_{l}^{\left(0.29-\frac{a}{d}{\rho }_{l}\right)}, \frac{a}{d}\le 2.5, {\rho }_{l}\le 0.1$ With web reinf.: $\frac{{V}_{n}}{{b}_{w}h}=\frac{29{\rho }_{l}+3.8{\rho }_{l}^{0.3} \sqrt{{f}_{c}^{\prime}}}{{\left(0.3\right)}^{{\Psi }_{vh}}\left(\frac{a}{d}\right)+0.47}, {\Psi }_{vh}={\rho }_{h}\left(\frac{{f}_{yh}}{{f}_{c}^{\prime}}\right)+{\rho }_{v}\left(\frac{{f}_{yv}}{{f}_{c}^{\prime}}\right)$

where *β*_s_ is coefficient of strut, *θ* is the angle between the strut and the longitudinal axis, w_s_ and w_t_ are the widths of the strut and tie, ${\varepsilon }_{s}$ is the tie's tensile strain, *ρ*_v_ is reinforcement ratio for VWR, and *ρ*_h_ is reinforcement ratio for HWR; the χ function is obtained for 10 ≤ fc′ ≤ 105 MPa.

While CATB, GPR, and LGBM models exhibit superior results, deriving an explicit design formula from these models is challenging. The black-box and difficult-to-interpret nature of these models hinders their practical implementation in engineering design. Therefore, this study tackles this challenge by introducing straightforward and practical explicit design formulas through the SR technique. As shown in Table 6, the proposed equations yield *μ* values of 1.003 and 1.004, *R*^2^ values of 0.917 and 0.937, and CoV values of 0.207 and 0.192 for the RCDBs without web reinforcement (WOR) and with web reinforcement (WWR) cases, respectively. Despite their slightly lower accuracy compared to the introduced ML models, these SR-derived formulas are more accessible and easier to interpret, encouraging their practical utility in engineering applications.

## Comparisons with closed-form models

**Figure 6:**
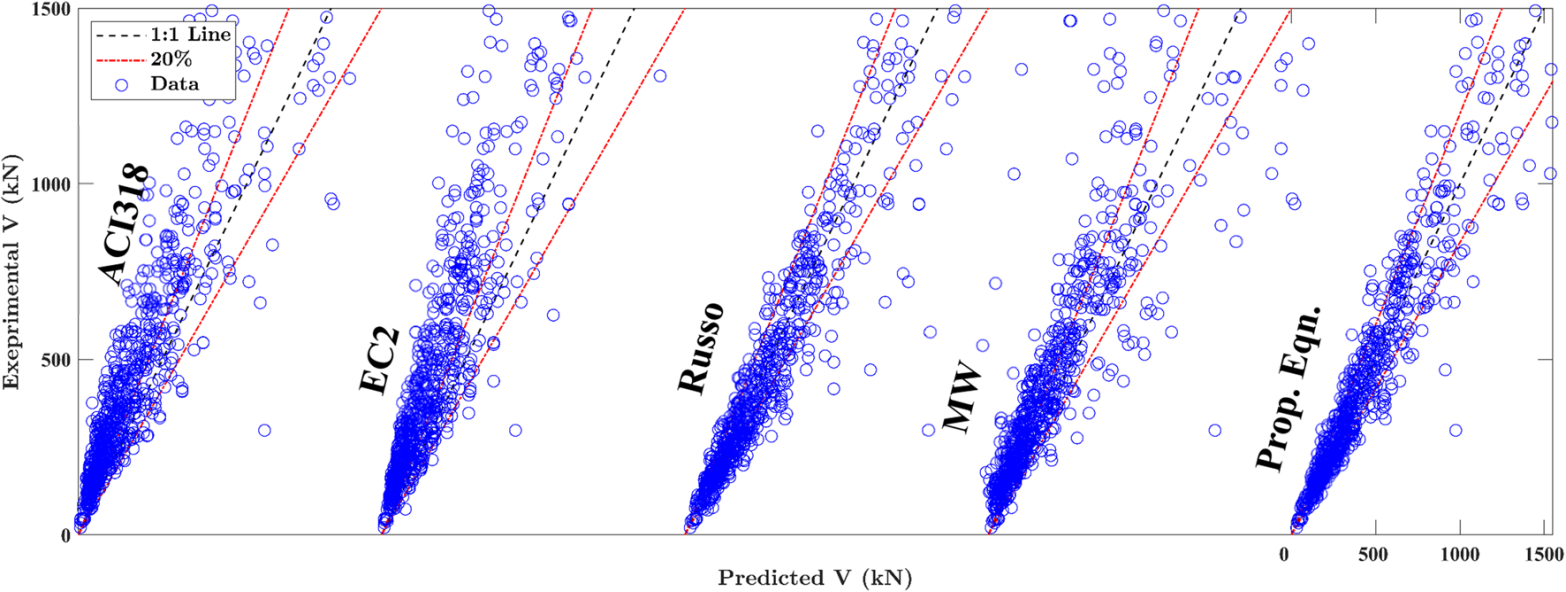
Comparison between proposed equations and previous models.

In this section, a comparison of the proposed equations with four present closed-form models (listed in Table 6), including two standard codes, i.e., ACI 318-19^17^, EC2^18^, and equations proposed by Matamoros and Wong (MW)^13^, and Russo et al.^14^ are introduced for performance evaluation. Table 7 summarises the statistical information about the predictive capability of these models compared to the proposed equations for two different reinforcement configurations, i.e., the case without web reinforcement (WOR) and the case with web reinforcement (WWR). The values of (*μ*, CoV) obtained by the proposed equations are (1.003, 0.207) and (1.004, 0.192) for WOR and WWR cases, respectively, which shows that these expressions perform well in terms of predictive stability and robustness compared to the present closed-form models. Additionally, Fig. 6 presents the scatter plots to illustrate the relationship between experimental and predicted results based on the entire database obtained by the proposed expressions and the four closed-form models. In Fig. 6, ACI 318-19, EC2, and MW expressions exhibit similar performance, with over-diagonal-skewed distribution, indicating that these models tend toward conservative prediction. On the other hand, the proposed equations demonstrate concentrated prediction-to-test ratios around unity, with (μ, CoV) values of (1.003, 0.198), marking the best results among these models. Furthermore, the CATBoost model displays superior performance with (*μ*, CoV) values of (1.01, 0.088), highlighting its excellent efficacy in employing ML techniques for shear strength prediction of RCDBs.

**Table 7:** Comparison of the developed ML models.

Metrics	Without web reinforcement (WOR)					With web reinforcement (WWR)					Overall					
	MW^13^	Russo^14^	EC2^18^	ACI^17^	Prop	MW^13^	Russo^14^	EC2^18^	ACI^17^	Prop	MW^13^	Russo^14^	EC2^18^	ACI^17^	CatB	Prop
Mean $\mu$	0.782	1.083	0.589	0.669	1.003	0.822	1.075	0.732	0.718	1.004	0.807	1.078	0.677	0.699	1.01	1.003
CoV	0.308	0.251	0.299	0.283	0.207	0.432	0.233	0.35	0.409	0.192	0.392	0.24	0.353	0.372	0.085	0.198
R2	0.824	0.866	0.487	0.671	0.917	0.634	0.942	0.722	0.559	0.937	0.664	0.932	0.692	0.579	0.986	0.935
MAPE	43.08	17.93	82.88	62.32	16.80	70.09	13.57	56.70	65.93	13.70	59.74	15.28	66.73	64.544	5.389	14.89
RMSE( kN)	168.6	147.3	288	230.6	115.9	474.2	189.3	413.2	520.6	196.7	386.7	174.4	370.2	433	79.1	170.3
a20-index	0.301	0.621	0.087	0.177	0.68	0.407	0.763	0.27	0.241	0.78	0.367	0.708	0.2	0.217	0.975	0.742

**Figure 7:**
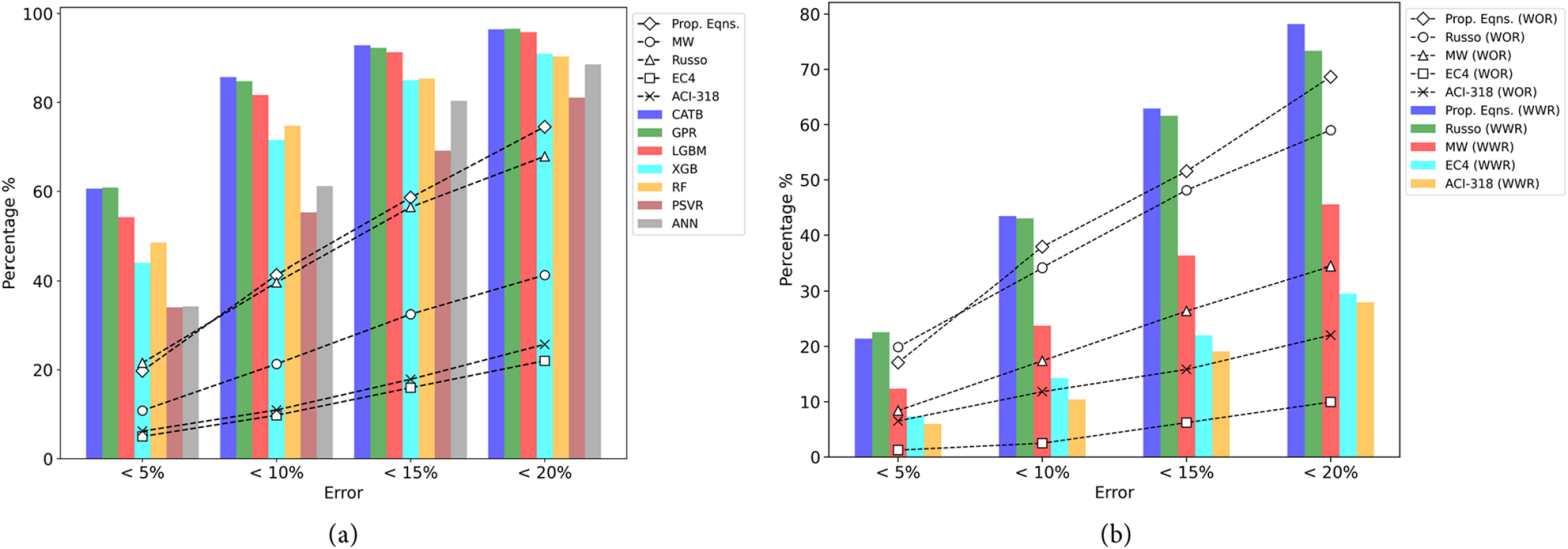
Prediction errors of design standards and established ML models. (**a**) The proposed equations, ML models and previous models, (**b**) The proposed equations and previous models for WOR and WWR cases.

Figure 7 illustrates the prediction errors of both design standards and the developed ML models. In Fig. 7a, CATB, GPR, and LGBM models demonstrate precision, with over 81% of test samples falling within the 10% error range. In contrast, the MW and Russo formulas exhibit 21% and 39% of test samples within the same error range, respectively. As noticed, ACI318 and EC2 provisions perform less effectively, capturing only 11% and 16% of test samples within the 10% error range, respectively. These results highlight the superior accuracy of most ML models, particularly CATB, GPR, and LGBM, in predicting the shear strength of RCDBs compared to traditional design standards. In Fig. 7b, the performance of the proposed equation and Russo formula for the WOR case is comparable, with a slight advantage for the proposed equation. The better performance of the proposed equation in the WOR case is evident in Table 7, where it exhibits a smaller error metric (i.e., CoV of 0.207) compared to the Russo formula (i.e., CoV of 0.251). In the WWR case, the proposed equation outperforms previous models, as shown in Fig. 7b, giving slightly better predictions (i.e., CoV of 0.192) compared to the Russo formula (i.e., CoV of 0.233) and outperforming ACI 318-19, EC2, and MW results, displaying almost twice the number of test samples as MW formulas and four times the number of test samples as ACI318 and EC2 for the same error ranges. Although the results of the proposed equations and the Russo formula are comparable, the proposed equations are more straightforward. Furthermore, all performance metrics for the proposed equations, outlined in Table 7, surpass those of the previously introduced mechanical models.

## Feature importance analysis

**Figure 8:**
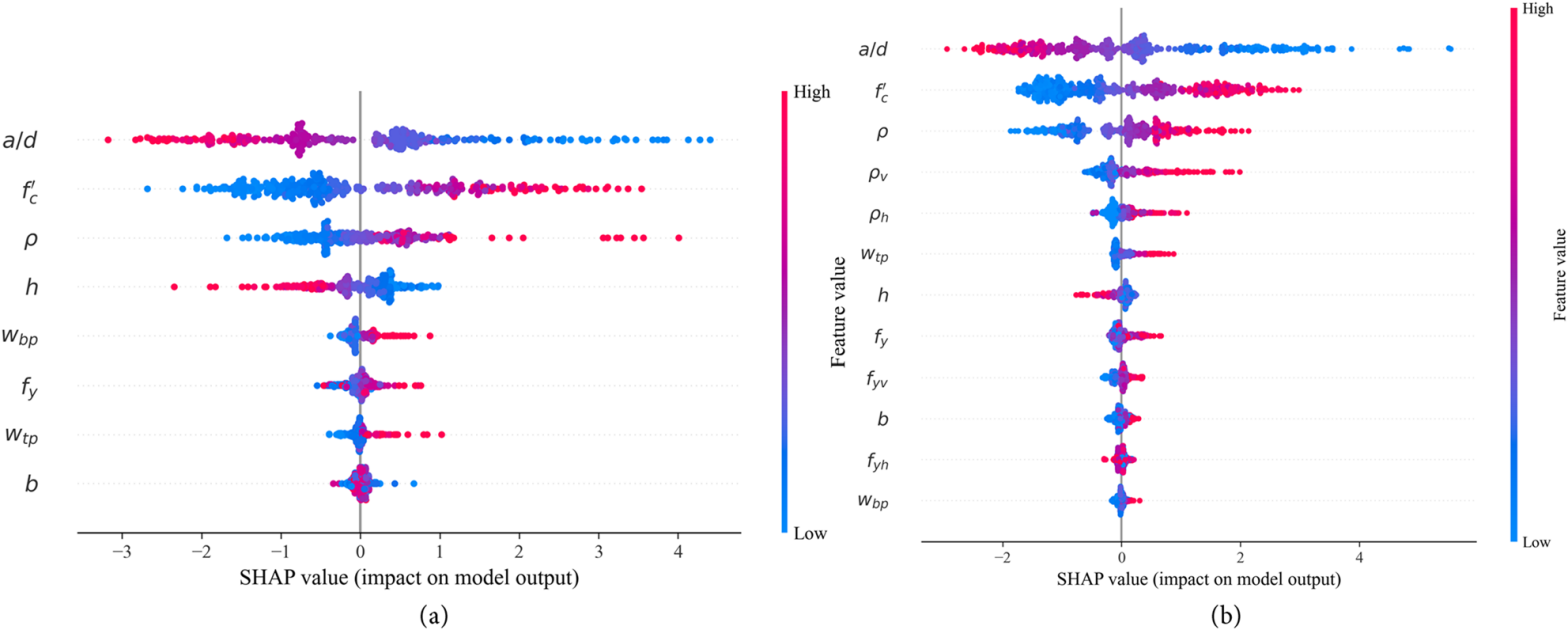
Features importance for inputs influencing shear strength of RC deep beams. (**a**) Database without web reinforcement, (**b**) database with web reinforcement.

Evaluating the influence of input parameters on the shear strength of RCDBs is a critical aspect of designing RCDBs. This study employs the Shapley Additive Explanation (SHAP) method to analyze the impact of input parameters on the shear strength parameter, *V*_*u*_/*b*_*w*_*h*^45^. Figure 8a and b display the SHAP feature importance of each input feature for the WOR and WWR databases, respectively. A feature importance value greater than zero indicates a positive correlation between the variable and the strength index. In contrast, a value less than zero signifies a negative impact on the strength index. The span-to-depth ratio (*a*/*d*), concrete strength (*f*_*c*_′), and longitudinal reinforcement ratio (*ρ*_*l*_) stand out as the most influential design parameters within the dataset for both WOR and WWR RCDBs. In addition, feature importance analysis shows that vertical and horizontal web reinforcement ratio (*ρ*_*v*_, *ρ*_*h*_) are the forth and fifth most important features for WWR database. The importance of the remaining variables' features is ranked in descending order.

Additionally, it can be observed that, except for the *a*/*d* ratio and beam height (h), all other input variables have a positive and mixed impact on the strength index. Increasing concrete strength, reinforcement ratios (*ρ*_*l*_, *ρ*_*v*_, *ρ*_*h*_), and their yield strength will enhance the shear strength of RCDBs, while *a*/*d* ratio and beam height (h) negatively influence shear strength. The negative impact of the *a*/*d* ratio aligns with experimental results conducted by Kani^41^, which showed that beams exhibit higher shear resistance at lower *a*/*d* values. Furthermore, increasing the beam height (*h*) reduces the shear resistance, as a deeper beam leads to deterioration of the shear transfer strength by aggregate interlock of the critical shear crack and relatively high energy release, thereby aggravating the reduction in shear resistance^46^.

## Reliability analysis

This section introduces the results of reliability indices for the shear strength of RCDBs for the CATB model and the two proposed equations. In addition, it assesses the existing design factors outlined in two existing code standards, including ACI318-19^17^ or EC2^18^. The limit state function *g* of shear strength of RCDBs^47^ can be defined as:

6 $$g=R-Q=\frac{1}{{\theta }_{\text{R}}}{V}_{uc}-\left(D+L\right)$$

where *R* is the random values of shear strength of RCDBs, defined as the predicted shear capacity (*V*_*uc*_) divided by the prediction-to-test ratio ${\theta }_{R}$, and *Q* is the random values of load effect, including the dead load (*D*) and live load (*L*), The value *V*_*uc*_ is calculated for each model from Table 6 with the partial resistance factors taken as unity, and using the random values of design variables given in Table 8. Using the distribution fit tool in Matlab, it was found that ${\theta }_{\text{R}}$ ratio is best fitted with lognormal distribution with mean and variance corresponding to each code standard, as indicated in Table 9. The nominal values *D*_*n*_ and *L*_*n*_ can be computed from the design resistance *V*_*d*_ for a given live-to-dead load ratio (*L*_*n*_/*D*_*n*_) as follows:

7 $${V}_{d}\left(\frac{{f}_{ck}}{{\gamma }_{c}},\frac{{f}_{y}}{{\gamma }_{s}}\right) \; or \; \phi {V}_{n}\left({f}_{ck},{f}_{y}\right)={S}_{d}\left(i.e.\; {\gamma }_{D}{D}_{n}+{\gamma }_{L}{L}_{n}\right)$$

8 $${D}_{n}=\frac{{R}_{d} \; or\; \phi {R}_{n}}{{\gamma }_{D}+{\gamma }_{L}\cdot k},\; {L}_{n}={D}_{n}\cdot k$$

where k is the live-to-dead load ratio *L*_*n*_/*D*_*n*_, the reduced designed resistance (*V*_*d*_) is extracted from dividing the characteristic strength of concrete and steel materials (${f}_{ck}$ and *f*_*y*_) by the material partial factors (*γ*_*c*_ and *γ*_*s*_)^18^ or multiplying the nominal resistance (*V*_*n*_) by a strength reduction factor (*ϕ*)^17^, and then *V*_*d*_ is balanced by the enlarged designed load effect (*S*_*d*_) to ensure a suitable safety margin. *S*_*d*_ is obtained by multiplying the nominal load values, including dead and live loads (*D*_*n*_ and *L*_*n*_), by, respectively, partial load factors (*γ*_*D*_ and *γ*_*L*_) and then combining them linearly. These partial factors are summarised in Table 9 for each code standard.

**Table 8:** Statistical properties of random variables.

Properties	Variables	Mean	Cov (%)	Std.	Distribution	Space	Refs.
Geometry	${b}_{w}$ (mm)	${b}_{w}+2.286$	–	4.826 (mm)	Normal	200	^47,48^
	*h* (mm)	*h*	2.0	–	Normal	{1000, 2000, 3000}	^49,50^
	*d* (mm)	*d-4.826*	–	12.7 (mm)	Normal	0.97 (h)	^47,48^
	*a*/*d*	*a*/*d*	–	–	Deterministic	{0.5, 1.25, 2.0}	^47^
Material	${f}_{c}^{\prime}$ (MPa)	$\chi {f}_{c}^{\prime}$*	10.1	–	Normal	{20, 40, 60}	^51,52^
	${f}_{y}$ (MPa)	$1.2{f}_{y}$	8.3	–	Lognormal	{235, 355, 420}	^50,53,54^
	${f}_{yh}$ (MPa)	$1.2{f}_{y}$	8.3	–	Lognormal	235	^50,53,54^
	${f}_{yv}$ (MPa)	$1.2{f}_{y}$	8.3	–	Lognormal	235	^50,53,54^
Reinforcement	${\rho }_{l}$	${\rho }_{l}$	1.25	–	Normal	{0.004, 0.012, 0.02}	^50,54,55^
	${\rho }_{hw}$	${\rho }_{hw}$	1.25	–	Normal	{0.002, 0.06, 0.01}	^50,54,55^
	${\rho }_{vw}$	${\rho }_{hw}$	1.25	–	Normal	{0.002, 0.06, 0.01}	^50,54,55^
Load	k (load ratio)	k	–	–	Deterministic	{0.5, 1.25, 2.0}	
	D (dead load)	1.05D	0.1	–	Normal	–	^24,56^
	L (live load)	L	0.18	–	Gumbel	–	^47,56^

*$$\chi =3.0469-0.13543{f}_{c}^{\prime}+0.31743{\left(0.1{f}_{c}^{\prime}\right)}^{2}-0.02413{\left(0.1{f}_{c}^{\prime}\right)}^{3}$$ ^51^.

**Table 9:** Load and resistance factors, the prediction-to-test ratio θ_R distributions, recommended strength reduction factor ϕ.

	ACI318^17^	EC2^18^	CATB	Prop.Eqn (WOR)	Prop.Eqn (WWR)
Load factor $\gamma$					
Dead load ${\gamma }_{D}$	1.2	1.35	1.2	1.2**	1.2**
Live load ${\gamma }_{L}$	1.6	1.5	1.6	1.6	1.6
Best-fit distribution ${\theta }_{\text{R}}$ (Table *)	Lognormal	Lognormal	Lognormal	Lognormal	Lognormal
Mean (Table *)	0.699	0.677	1.01	1.003	1.004
CoV (Table *)	0.372	0.353	0.085	0.207	0.192
Target reliability $\beta$	3.5^59^	3.8^18^	5.29*	3.5**	3.5**
Evaluated strength reduction factor $\phi$ for the target reliability index	0.58	0.78	1.0	0.76	0.78

*Target reliability $\beta =5.29$ evaluated for strength reduction factor $\phi =1.0$.

**Load factors and target reliability $\beta$ are assumed to be identical to these of ACI318.

The safety level of structures can be measured by the reliability index *β*, a factor related to the failure probability *P*_*f,*_ as follows^57^:

9 $$\beta ={\Phi }^{-1}\left({P}_{f}\right)$$

where Φ is the standard cumulative distribution function. Monte Carlo simulation (MCS) is employed to determine the reliability index due to its simplicity, insensitivity to problem dimensions, and satisfactory accuracy^56^. In MCS, the failure probability can be calculated as

10 $${P}_{f}=\frac{{N}_{fail}}{N}$$

where *N* and *N*_*fail*_ are the total number of simulations and the number of failed simulations (when the limit state function is violated, i.e. g ≤ 0), respectively. To accurately predict the reliability index of the design codes, the uncertainty or randomness of all input variables, including material geometry and loads, should be considered^47^. Thirteen random variables are considered in this study, and the statistical properties are summarised in Table 8. The random numbers of variable inputs are generated with continuous variations stochastically chosen from their respective distribution functions (Table 8) and drawn from a wide range of geometric and geometry parameters of RCDBs configurations. They include three values of concrete compressive strength *f*_*c*_′ = {20, 40, 60} MPa, three values of beam height *h* = {1000, 2000, 3000} mm, three ratios for longitudinal, VWR, and VWR ratios, three values of longitudinal steel yield stress *f*_*yl*_ = {235, 355, 420} MPa, four ratios for *a*/*d* = {0.5, 1.0, 1.5, 2.0}, four ratios of *L*_*n*_/*D*_*n*_ = {0.5, 1.0, 1.5, 2.0}. In total, there are 3 × 3 × 3 × 3 × 3 × 3 × 4 × 4 = 11,664 beam configurations considered for each considered model. The target safety level stipulated by ACI318^17^ and EC2^18^ provisions for the shear strength of RCDBs are 3.5 and 3.8, respectively. The accuracy of MCS is dependent on the number of samples *N*. The number of samples *N* used in this study for achieving a reliability index β equal to 3.8 with acceptable accuracy (CoV of 5%) is 5,528,430^58^.

**Figure 9:**
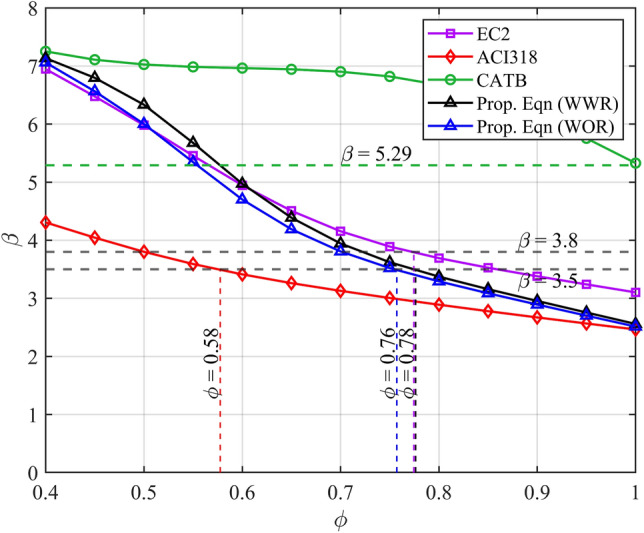
Variation of reliability index *β* in terms of strength reduction factor *ϕ* for the proposed equations, EC2 and ACI318.

As illustrated in Fig. 9, the strength reduction factors (*ϕ*) for the proposed equations are 0.76 and 0.78 for cases without web reinforcement (WOR) and with web reinforcement (WWR), respectively, at a reliability index value of 3.5. The higher strength reduction factor in the WWR case is attributed to the ductile behaviour exhibited by beams with web reinforcement compared to those without web reinforcement. Furthermore, the strength reduction factors corresponding to the target reliability for the shear strength design of RCDBs according to ACI318 and EC2 are 0.58 and 0.78, respectively. While the strength reduction factor for the proposed equation without web reinforcement is comparable to that of EC2 (*ϕ* = 0.78), a notable distinction lies in their mean values of *θ*_*R*_. As detailed in Table 8, the proposed equation yields a mean value for *θ*_*R*_ close to 1.0, whereas the EC2 code standard yields a smaller mean value of 0.677. As per Eqs. (6) and (10), smaller mean values of *θ*_*R*_ correspond to low failure probability and relatively high strength reduction factors. Therefore, the reliability associated with the proposed equations surpasses the reliability results obtained by applying code standards. Moreover, Fig. 9 reveals that the CATB model, when used with *ϕ* = 1.0, can achieve a high-reliability index of 5.29. This high reliability index is attributed to the low CoV error metric of the CATB model compared to other models, as outlined in Table 9, indicating the reliability of using ML models in enhancing the predictive accuracy for the shear strength of RCDBs.

## Conclusions

In conclusion, this study compiled a comprehensive experimental database of 840 experimental tests for the shear strength of RCDBs from various research papers. It employed eight machine learning models optimised using the Bayesian Optimization (BO) technique. In addition, proposed expressions are presented for designing RCDBs. From the evolution results, the following conclusions can be drawn:

- The CATBoost, GPR, and LGBM models exhibited outstanding accuracy and stability, surpassing traditional design standards. The CATBoost model demonstrated the best prediction accuracy and generalisation ability, outperforming other ML models.
- The introduced explicit design formulas, derived through symbolic regression, are straightforward and robust, offering simplicity and robustness compared to previous approaches.
- Comparison with closed-form models and design standards, such as ACI 318-19 and EC2, highlighted the efficiency of the proposed equations, which displayed superior predictive stability and robustness.
- SHAP analysis revealed that increasing concrete strength, reinforcement ratios (*ρ*_*l*_, *ρ*_*v*_, *ρ*_*h*_) and their yield strength will enhance the performance of RCDBs, while increasing *a*/*d* ratio and beam height (h) will negatively impact the shear strength parameter, *V*_*u*_/*b*_*w*_*h*.
- The reliability analysis indicated that the CATBoost model and proposed equations surpassed code standards regarding reliability and accuracy.

In summary, integrating the ML-based approach presents a promising approach for accurately predicting the shear strength of RCDBs, providing valuable insights for engineering applications.

### Supplementary Information

Supplementary Information.

## Data Availability

All data generated or analysed during this study are included in this published article and available in a public repository: https://github.com/kmegahed/Deep-beam-ML-models.
