# Supplementary material for: Prediction and reliability analysis of shear strength of RC deep beams
Source: Sci Rep. 2024 Jun 25;14:14590. doi: 10.1038/s41598-024-64386-w (PMC11199521; doi:10.1038/s41598-024-64386-w)
Supplement: Supplementary file 1 — Supplementary Information. [file 41598_2024_64386_MOESM1_ESM.zip › Sup data/eqn.docx]

Unreinforced
Error: 0.216 $\left( rho +\frac{0.036}{\left( a_{d}+ rho \right)_{d}^{a}} \right)*\left( -0.029*fc*\left( a_{d}- 0.275 \right)^{0.091}+ 3.780 \right)$

Error: 0.217 $1.112*\frac{rho^{0.537}}{a_{d}^{ad}+ 0.049*fc^{1.331}*rho}+\frac{1.112}{fc}$

Error: 0.217 $a_{d}^{rho}*\left( -0.140 +\frac{\left( a_{d}*rho \right)^{0.313}+ \left( fc + 0.199 \right)^{-0.375}}{a_{d}+ 0.935} \right)$

Error: 0.211 $a_{d}*\left( fc*\frac{a_{d}*rho + 0.197}{rho} \right)^{a_{d}*\left( 0.044*a_{d}- 0.363 \right)}$

Error: 0.213 $\left( a_{d}*\frac{fc}{rho}+ 0.065*fc^{2} \right)^{-0.023*a_{d}- 0.152}- 0.121$

Error: 0.205 ${0.483}^{0.075*fc*rho}*\left( 12.551*\frac{rho}{fc} \right)^{0.146*a_{d}+ 0.232}$

Error: 0.217 $a_{d}^{1.088}*\left( 1.052*rho \right)^{0.533*a_{d}+ 0.040*fc*rho}+\frac{1.262}{fc}$

Error: 0.206 $\frac{rho^{0.376}}{rho + 0.376*rho^{a_{d}}+ 0.376*\left( a_{d}+ fc \right)^{0.207*a_{d}+ 0.179}}$

Error: 0.205 $\left( 0.646*fc + 0.046*\frac{0.851*fc + 4.423}{rho} \right)^{-0.150*a_{d}- 0.261}$

Error: 0.217 $\left( a_{d}*\left( a_{d}+ 0.114*fc - 0.625 +\frac{0.058}{rho} \right)+\frac{0.851}{a_{d}} \right)^{-0.890}$

Error: 0.214 $1.098*\left( fc +\frac{a_{d}^{2}*fc + 2.219}{rho} \right)^{\frac{0.099}{rho - 0.870}}- 0.308$

Error: 0.211 (0.277*a_d^2*(a_d + fc)/rho + fc)^(0.227/(rho - 1.176)) - 0.143

Error: 0.214 $\left( 1.732*a_{d}^{2}*\frac{rho}{fc} \right)^{0.304*a_{d}}*\left( a_{d}+ 0.217 \right)$

Error: 0.217 $\left( fc*\frac{a_{d}*rho + 0.731}{rho} \right)^{-0.054*a_{d}- 0.152}- 0.080$

Error: 0.231 $a_{d}^{1.390}*\left( -0.001*fc + rho + 0.163 -\frac{0.000124}{rho} \right)_{d}^{a}+ 1.216*rho$

Error: 0.213 $\frac{5.958*rho + \left( 2.946*rho + 0.348 \right)_{d}^{a}}{{1.020}^{a_{d}+ fc}+ 1.460}$

Error: 0.208 $\frac{\left( 0.129*fc - 0.245 +\frac{0.330}{rho} \right)^{a_{d}*\left( rho - 0.245 \right)+ rho}}{fc^{0.365}}$

Error: 0.213 $\frac{1.100}{\left( \left( a_{d}+\frac{rho}{a_{d}} \right)*\frac{a_{d}^{2}*fc + fc}{rho} \right)^{0.137}}- 0.209$

Error: 0.216 $\frac{0.410}{\left( 0.017*a_{d}*\left( a_{d}+ fc \right)+ 0.008*\frac{a_{d}}{rho}+ 1 \right)^{1.423}}$

Error: 0.206 $\left( 0.324*fc^{2}*rho + 0.113*\frac{fc}{rho} \right)^{-0.134*a_{d}- 0.224}$

Error: 0.214 1$.104*\frac{a_{d}^{0.772}}{\left( fc + 0.622*\frac{fc}{a_{d}^{2}*rho} \right)^{0.286*a_{d}}}$

Error: 0.206 $3.331*rho^{0.361}*\left( 3*a_{d}+ fc + 3.331 \right)^{-0.185*a_{d}- 0.268}$

Error: 0.221 $rho^{0.970}+ \left( a_{d}- 0.050 \right)*\left( a_{d}*rho - 0.001*fc + 0.171 \right)_{d}^{a}$

Error: 0.209 $0.168*{0.697}^{a_{d}*\left( a_{d}+ 0.019*fc \right)}+ \left( \frac{rho}{0.020*fc + 0.049} \right)^{0.832}$

Error: 0.209 $\left( a_{d}^{2}*\left( a_{d}+ fc \right)*\frac{0.843*rho + 0.116}{rho}+ fc \right)^{-0.258}- 0.088$

Error: 0.216 $\left( a_{d}^{2}*\left( a_{d}^{2}+ fc \right)*\left( fc*rho +\frac{0.155}{rho} \right) \right)^{-0.069}- 0.526$

Error: 0.215 $\left( a_{d}^{2}*\frac{a_{d}^{2}+ fc - 1.819}{rho}+\frac{fc}{a_{d}} \right)^{-0.110}- 0.294$

Error: 0.224 $\frac{{0.591}_{d}^{a}}{{0.682}^{0.372*fc - 1.617}+ fc^{0.337}}+ rho^{0.682}- 0.100$

Error: 0.208 $1.443*\left( -1.150 +\frac{1.023*fc*rho + 0.374}{rho} \right)^{-0.178*a_{d}+ rho - 0.409}$

Error: 0.219 $a\_d*\frac{rho}{rho + \left( a_{d}^{2}*\left( 0.097*fc*rho + 0.065 \right)+ rho \right)^{1.174}}$

Error: 0.221 ${0.985}^{fc}*\frac{rho + \left( 1.431*fc \right)^{rho}- 0.837}{{0.046}^{a_{d}}+ a_{d}}$

Error: 0.211 $\left( \frac{rho}{fc*\left( a_{d}*rho + 0.310 \right)+ 0.310} \right)^{0.073*a_{d}+ 0.181}- 0.048$

Error: 0.223 $a_{d}^{1.138}*\left( a_{d}*rho - 0.001*fc + 0.160 \right)_{d}^{a}+ rho$

Error: 0.214 $\frac{a_{d}^{0.762}}{\left( fc +\frac{fc}{a_{d}*rho*\left( 2*a_{d}+ 0.955 \right)} \right)^{0.296*a_{d}}}$

Error: 0.218 $1.173*rho^{0.162*a_{d}+ 0.162*rho}*\left( fc^{-0.447}+ 2*rho \right)- 0.004$

Error: 0.208 $3.659*\frac{{0.477}^{a_{d}}}{\left( \left( fc +\frac{2.490}{a_{d}} \right)*\frac{rho + 0.101}{rho} \right)^{0.452}}$

Error: 0.212 $\frac{a_{d}*rho + rho^{0.312}}{\left( a_{d}+ 0.202*fc \right)^{0.347*a_{d}}}$

Error: 0.217 $rho^{0.835}+\frac{rho + 0.155}{{1.012}^{a_{d}*\left( a_{d}*fc + 2.479 \right)}}$

Error: 0.205 $\left( \frac{rho}{0.048*fc + 0.227} \right)^{a_{d}*\left( rho + 0.135 \right)+ 0.262}$

Error: 0.205 $\left( 0.509*fc*\frac{rho + 0.103}{rho} \right)^{-0.152*a_{d}- 0.251}$

Error: 0.214 $\frac{rho^{0.351}}{{0.159}^{a_{d}- 0.076}+ {1.013}^{fc}*\left( a_{d}- 0.076 \right)}$

Error: 0.210 $1.355*{0.987}^{a_{d}+ fc}*\frac{rho^{0.424}}{a_{d}+ rho^{0.779*a_{d}}}$

Error: 0.221 $\frac{\left( a_{d}*rho \right)^{0.257}}{fc^{0.257*a_{d}}}$

Error: 0.218 $\frac{rho + \left( a_{d}*rho \right)^{0.257}}{fc^{0.264*a_{d}}}$

Error: 0.217 $\frac{a_{d}*rho + \left( a_{d}*rho \right)^{0.257}}{fc^{0.264*a_{d}}}$

Error: 0.217 $\frac{a_{d}*rho + \left( a_{d}*rho \right)^{0.257}}{\left( a_{d}+ fc - 0.639 \right)^{0.264*a_{d}}}$

Error: 0.204 $\left( 0.665*fc + 0.072*\frac{fc}{rho} \right)^{-0.141*a_{d}- 0.240}$

Error: 0.212 $\left( \frac{rho}{0.019*a_{d}*\left( a_{d}^{3}*fc + fc \right)+ 0.019*fc} \right)^{0.405}$

Error: 0.207 $1.313*fc^{-0.235*a_{d}- 0.126}*rho^{-1.364*a_{d}*rho + 0.258}$

Error: 0.209 $\left( 0.335*a_{d}*\left( fc + 0.194*\frac{fc}{rho} \right) \right)^{-0.075*a_{d}- 0.321}$

Error: 0.212 $1.660*fc^{-0.220*a_{d}- 0.102}*rho^{0.319}$

Error: 0.214 $1.182*{0.987}^{fc}*\frac{rho^{0.390}}{a_{d}+ \left( rho + 0.034 \right)_{d}^{a}}$

Error: 0.208 $2.496*fc^{-0.211*a_{d}- 0.183}*rho^{0.363}$ best*******

Error: 0.214 $\frac{a_{d}+ 0.162}{\left( \frac{fc}{a_{d}*rho*\left( a_{d}+ 0.128 \right)} \right)^{0.281*a_{d}}}$

Error: 0.206 $2.260*\left( fc +\frac{fc}{a_{d}*rho} \right)^{-0.142*a_{d}- 0.220}$ best*******

Error: 0.214 $\frac{a_{d}*rho + rho^{0.287}}{\left( 0.494*fc \right)^{0.287*a_{d}}}$

Error: 0.211 ${0.982}^{0.638*a_{d}*fc}*a_{d}*\left( {0.163}_{d}^{a}+ 2*rho \right)+ rho$

Error: 0.211 ${0.982}^{0.638*a_{d}*fc + 1.834}*a_{d}*\left( {0.163}_{d}^{a}+ 2*rho \right)+ rho$

Error: 0.210 $\left( a_{d}+ 0.112*fc \right)^{-0.417*a_{d}- 0.417*rho}*\left( a_{d}*rho + rho^{0.327} \right)$

Error: 0.215 $\frac{rho^{0.310}}{\left( 0.259*a_{d}+ 0.259*fc \right)^{0.310*a_{d}}}$

Error: 0.213 $\frac{rho^{0.310}}{\left( a_{d}+ 0.259*fc - 1.272 \right)^{0.319*a_{d}}}$

Error: 0.209 $fc^{-0.228*a_{d}- 0.102}*rho^{-a_{d}*rho - rho + 0.228}$

Error: 0.209 $1.002*fc^{-0.228*a_{d}- 0.102}*rho^{-a_{d}*rho - rho + 0.228}$

Error: 0.214 $\frac{a_{d}+ 0.139}{\left( \frac{fc}{a_{d}^{2}*rho} \right)^{0.274*a_{d}}}$

Error: 0.211 $\left( \frac{fc}{a_{d}*rho} \right)^{-0.217*a_{d}- 0.101}*\left( a_{d}+ 0.626 \right)$

Error: 0.212 $\left( \frac{fc}{rho*\left( a_{d}+ rho \right)} \right)^{-0.216*a_{d}- 0.086}*\left( a_{d}+ 0.398 \right)$

Error: 0.206 $2.072*\left( \frac{fc + rho}{a_{d}*rho} \right)^{-0.140*a_{d}- 0.212}$

Error: 0.212 $\frac{rho^{0.339}}{\left( a_{d}+ 0.071*fc \right)^{0.442*a_{d}}}$

Error: 0.208 $1.128*rho^{0.356}*\left( a_{d}+ 0.051*fc \right)^{-0.442*a_{d}- 0.157}$

Error: 0.207 $3.641*\frac{\left( \frac{0.433}{0.002*fc + 0.834} \right)^{a_{d}}}{\left( \frac{fc}{rho} \right)^{0.330}}$

Error: 0.212 $5.370*\frac{{0.480}^{a_{d}}}{\left( \frac{fc}{rho} \right)^{0.379}}$

Error: 0.217 $a_{d}*\left( rho*\frac{a_{d}^{2}+ 1.268}{fc} \right)^{0.286*a_{d}}$

Error: 0.212 $1.681*\frac{rho^{0.171*a_{d}+ 0.159}}{\left( 0.389*fc + 1 \right)^{0.425}}$

Error: 0.207 $\left( 2*a_{d}+ 0.131*\frac{fc}{a_{d}*rho} \right)^{-0.196*a_{d}- 0.153}$

Error: 0.207 $\left( 0.131*\frac{fc}{a_{d}*rho} \right)^{-0.196*a_{d}- 0.153}$ best*******

Error: 0.207 $\left( 2*a_{d}+ 0.131*\frac{fc}{a_{d}*rho} \right)^{-0.196*a_{d}- 0.153}$

Error: 0.214 $\frac{rho}{0.043*a_{d}+ 0.043*fc*rho*\left( {2.190}_{d}^{a}- 0.557 \right)+ 2*rho}$

Error: 0.204 $\left( rho*\left( -0.037 + 2.527*\frac{a_{d}+ 1.025}{fc} \right) \right)^{0.156*a_{d}+ 0.156}$

Error: 0.205 $\left( 2.684 + fc*\frac{0.017*fc +\frac{0.136}{rho}}{a_{d}} \right)^{-0.189*a_{d}- 0.154}$

Error: 0.208 $\left( fc +\frac{0.243}{rho} \right)^{-0.183*a_{d}+ 2*rho - 0.352}$

Error: 0.215 $-0.057*a_{d}^{0.755}+\frac{1.177}{\left( a_{d}*\frac{fc}{rho} \right)^{0.235}}$

Error: 0.226 $2.148*rho - 0.016 +\frac{0.722}{\left( 0.366*fc \right)^{0.519*a_{d}}+ 2.148}$

Error: 0.207 $\left( 8.545*\frac{rho}{fc} \right)^{0.132*a_{d}+ 0.224}$ best*******

Error: 0.205 $\left( 2.063*\frac{rho}{fc*\left( rho + 0.241 \right)} \right)^{0.132*a_{d}+ 0.220}$

Error: 0.227 $0.701*a_{d}*rho^{0.477*a_{d}}+\frac{a_{d}+ 0.426}{a_{d}*fc}$

Error: 0.226 $0.701*a_{d}*rho^{0.477*a_{d}}+\frac{a_{d}+ rho^{0.195}}{a_{d}*fc}$

Error: 0.215 $rho + \left( a_{d}+ rho \right)*\left( fc +\frac{0.220}{rho} \right)^{-0.448*a_{d}- 0.085}$

Error: 0.213 $rho + \left( a_{d}+\frac{rho}{a_{d}} \right)*\left( fc +\frac{0.220}{rho} \right)^{-0.448*a_{d}- 0.085}$

Error: 0.217 $\frac{a_{d}}{\left( 0.412*fc +\frac{0.136}{a_{d}*rho} \right)^{0.657*a_{d}}}+ rho$

Error: 0.215 $\frac{a_{d}}{\left( 0.415*fc +\frac{rho + 0.143}{a_{d}*rho} \right)^{0.642*a_{d}}}+ rho$

Error: 0.204 $\left( 0.013*fc^{2}+ 0.098*\frac{fc}{rho} \right)^{-0.135*a_{d}- 0.233}$

Error: 0.205 $\left( 0.853*fc*\frac{rho + 0.098}{rho} \right)^{-0.135*a_{d}- 0.234}$

Error: 0.205 $\left( 0.689*fc + 0.078*\frac{fc}{rho} \right)^{-0.139*a_{d}- 0.236}$

Error: 0.215 $\frac{rho^{0.302}}{\left( 0.315*fc \right)^{0.302*a_{d}}}$

Error: 0.215 $\frac{rho^{0.302}}{\left( 0.315*fc - 0.008 \right)^{0.302*a_{d}}}$

Error: 0.213 $\frac{a_{d}*rho + rho^{0.298}}{\left( 0.347*fc \right)^{0.321*a_{d}}}$

Error: 0.213 $\frac{a_{d}*rho + rho^{0.298}}{\left( 0.347*fc + 0.347*rho \right)^{0.321*a_{d}}}$

Error: 0.214 $\left( 0.023*a_{d}^{3}*\frac{fc}{rho}+ fc \right)^{rho - 0.461}$

Error: 0.214 $\left( 0.022*a_{d}^{3}*\frac{fc}{rho}+ fc + 2.129 \right)^{rho - 0.461}$

$$\left( a_{d}*\left( 106.7*a_{d}*rho*\left( s2 +\frac{fck}{a_{d}}-2.14*a_{d} \right)+ 2.18*a_{d}+2.18*s1 \right) \right)^{0.39}$$

$a_{d}^{0.36}*\left( \mathrm{rho}^{0.688}*\left( a_{d}*s2 + f_{c}+ 2*s1 -\frac{0.137239827231015}{\mathrm{rho}} \right)+ 3.17 \right)$ Error 0.1776

$6.497*\left( a_{d}*rho*\left( f_{c}-\frac{1.129634}{a_{d}}-159.49*a_{d}*rho + a_{d}*s1 + s2 \right) \right)^{0.375}$ Error: 0.1638

$\left( a_{d}*\left( 1.48*a_{d}*s1 + rho*\left( -81.35*a_{d}^{2} + 55.9011690004935*f\_c - 116.08 \right) \right) \right)^{0.4408813}+ 0.63$

$6.39*\left( a_{d}*rho*\left( 0.0763*a_{d}*f\_c*\left( s1 - 1.827 \right)+ f\_c + s2 - 1.484 \right) \right)^{0.366}+ 0.0336$

$0.94*a_{d}^{0.994}+ a_{d}^{\frac{0.91}{a_{d}}}+ \mathrm{rho}^{0.68}*\left( a_{d}*1.508*\left( s1 + s2 \right)+ f\_c \right)+ 0.713$ Error: 0.1879

$0.156*a_{d}*s1 + 6.3985*\left( a_{d}*rho*\left( f_{c}+ 1.05*s2 - 7.76 \right) \right)^{0.369}+ 0.156$

$\frac{\left( rho*\left( a_{d}*\left( a_{d}*s1 + fck + s2 \right)- 0.58 \right) \right)^{1.32*rho + 0.438}}{a_{d}*rho + 0.13}$ error 0.1635

$6.1*\left( \left( a_{d}*\left( -1.6*a_{d}+ f_{c} \right)- 0.14 \right)*\left( rho + 0.001*s1*\left( a_{d}+ 0.75 \right)+ 0.001*s2 + 4.97*{10}^{5} \right)- 0.0084 \right)^{0.39}$

$6.34*\left( rho*\left( a_{d}*\left( a_{d}*\left( s1-1.53*a_{d} \right)+ f\_c \right)- 5.25 \right)+ 0.022 \right)^{0.387}+ 0.325$ error 0.1699

$6.32*\left( 0.022*a_{d}^{2}*\left( s1 +s2 \right)+ a_{d}*rho*\left( f_{c}- 1.58-1.82*a_{d}^{2} \right) \right)^{0.39}$ error 0.1616

$a_{d}+ 1.598*\frac{\left( rho*\left( 1.56*a_{d}*s1 + 1.17*f_{c} \right) \right)^{0.676}}{rho + \left( 1.17*rho \right)^{a_{d}}+ 0.345}+ 0.84$

$\left( a_{d}*\left( 135.75*rho + 0.549 \right)*\left( -2.18*a_{d}+ fck + 1.858*s1 + 1.37*s2 - 1.76 \right) \right)^{0.377}- 0.48$

$6.495*\left( a_{d}*rho*\left( a_{d}^{2}*\left( s1 - 2.48 \right)+ f_{c} + s2 -\frac{2.48}{a_{d}} \right) \right)^{0.36}+ 0.054$

PC['vert']=PC['rho_v']*PC['fyv'];PC['horz']=PC['rho_h']*PC['fyh']

PC['vn']=PC['V']/(PC['h'] * PC['b'])*1000/PC['f_c']

PC['f_c']=PC['f_c']/30.0

PC['s1']=PC['vert']/PC['f_c'];PC['s2']=PC['horz']/PC['f_c']

$\left( \frac{2*rho}{f_{ck}*\left( a_{d}^{2}- 0.039*a_{d}*\left( a_{d}*s1 + s2 \right)+ 0.54 \right)} \right)^{2.549*rho + 0.42}$ Error: 0.167

${1.017}^{1.592216*a_{d}*f_{ck}*s1 + s2}*\frac{rho^{0.286}}{{1.12}^{a_{d}*\left( a_{d}+ 0.282 \right)}+ 0.803*a_{d}*f_{ck}}$ Error: 0.1608

Error: 0.1617 $\frac{{0.758}_{d}^{a}}{\left( a_{d}*f_{ck}*\left( a_{d}*\left( -2.0*s1 - 2.9*s2 + 5.8 \right)+\frac{f_{ck}+ 0.72}{rho} \right) \right)^{0.326}}$

Error: 0.1577 $\frac{{0.99}^{f_{ck}*s2}}{{0.382}^{a_{d}}+ a_{d}*f_{ck}*\left( \left( f_{ck}*rho*\left( s1 + s2 + 2.13 \right) \right)^{-0.443}+ 0.403 \right)+ 2.13}$

Error: 0.16 $\left( \frac{rho}{a_{d}*\left( f_{ck}*\left( a_{d}*\left( 0.119*a_{d}+ f_{ck} \right)*\left( a_{d}-0.22*s1-0.22*s2+1.097 \right)+ f_{ck} \right)+ 1.097 \right)} \right)^{0.329}$

Error: 0.162 $\left( a_{d}*\frac{f_{ck}*\left( a_{d}*\left( f_{ck}+ 0.46 \right)*\left( a_{d}- 0.105*s1 - 0.105*s2 \right)+ 1.31*f_{ck} \right)+ 0.93}{rho} \right)^{-0.33}$

Error: 0.164 $\frac{rho^{0.27}}{{0.93}^{s1 + s2 + 1.47}+ f_{ck}^{rho + 0.64}*\left( 1.19*a_{d}+ rho^{1.3*a_{d}} \right)}$

Error: 0.164 $\left( \left( a_{d}+ 0.48 \right)*\frac{a_{d}*f_{ck}*\left( f_{ck}+ 0.19 \right)*\left( a_{d}- 0.073*s1 - 0.073*s2 \right)+ f_{ck}}{rho} \right)^{-0.35}$

Error:0.16 $\left( \frac{rho}{a_{d}^{2}*f_{ck}*\left( f_{ck}+ \left( f_{ck}+ 0.91 \right)*\left( a_{d}*\left( -0.087*f_{ck}*s1 - 0.087*s2 \right)+ a_{d} \right) \right)+ f_{ck}} \right)^{0.33}$

Error: 0.159 $\frac{rho + 0.89*\left( f_{ck}^{2}*rho \right)^{0.265}}{{0.98}^{2.385*\left( s1 +s2 \right)}*a_{d}^{1.28}*f_{ck}+ f_{ck}}$ $\frac{P}{hb}=\frac{rho + 0.89*\left( f_{ck}^{2}*rho \right)^{0.265}}{{0.98}^{2.385*\left( s1 +s2 \right)}*a_{d}^{1.28}+1.0}$

$$\frac{rho + 0.89*\left( fck^{2}*rho \right)^{0.265}}{{0.98}^{2.385*s1 + 2.385*s2}*a_{d}^{1.28}*fck + fck}$$

Error: 0.164 $\frac{rho^{0.29}}{{0.92}^{0.74*\left( s1 + s2 \right)}+ a_{d}*\left( a_{d}*rho*\left( a_{d}- 1.2 \right)+ f_{ck}^{0.74} \right)}$

Error: 0.165 $\frac{rho^{0.28}}{{0.94}^{f_{ck}*\left( a_{d}+ s1 + s2 - 1.82 \right)}+ {1.023}^{a_{d}^{2}}*a_{d}*f_{ck}^{0.82}}$

Error: 0.1617 $\frac{rho^{0.268}}{{0.98}^{s1}+ 1.089*a_{d}*\frac{0.14*a_{d}+ f_{ck}}{\left( a_{d}*f_{ck}+ s1 + s2 \right)^{0.168}}}$

Error: 0.166 $\frac{0.85* {0.686}_{d}^{a}}{\left( 0.606*f_{ck}*\frac{0.85*a_{d}*f_{ck}+ 0.52}{rho}- s2 \right)^{0.32}}$

Error: 0.163 $\left( \frac{rho}{a_{d}*\left( a_{d}+ f_{ck}^{2}*\left( a_{d}^{2}*\left( 2.6-0.25*\left( s1+s2 \right) \right)+1.0 \right) \right)+ f_{ck}} \right)^{0.31}$

PC['vn']=PC['V']/(PC['h'] * PC['b'])*1000/PC['f_c']

PC['f_c']=PC['f_c']/30.0

PC['ss']=PC['vert']*2+PC['horz']

PC['s1']=PC['vert']/PC['f_c'];PC['s2']=PC['horz']/PC['f_c']

features=['ad','f_c','rho','s1','s2']

X=PC[features];y=PC['vn']

Error: 0.168 $\left( \frac{rho}{rho + \left( a_{d}+ rho \right)*\left( -0.039*f_{ck}*\left( s1 + s2 \right)+ f_{ck} \right)} \right)^{1.562*a_{d}*rho + 0.422}$

Error: 0.163 $\left( \frac{rho}{a_{d}*f_{ck}*\left( a_{d}*\left( a_{d}+ f_{ck}- 0.059*\left( a_{d}+ f_{ck} \right)*\left( s1 + s2 \right) \right)+ f_{ck} \right)+ 0.374} \right)^{0.340}$

Error: 0.164 $\left( \frac{rho}{a_{d}*\left( -0.028*a_{d}*f_{ck}*\left( s1 + s2 \right)+ f_{ck} \right)*\left( a_{d}^{2}+ f_{ck}+ 0.655 \right)} \right)^{0.357}$

Error: 0.163 $\left( \frac{rho}{a_{d}*\left( f_{ck}+ 0.134 \right)*\left( f_{ck}+ \left( a_{d}+ f_{ck} \right)*\left( a_{d}- 0.041*\left( a_{d}+ f_{ck} \right)*\left( s1 + s2 \right) \right) \right)+ 0.338} \right)^{0.338}$

Error: 0.163 $\left( \frac{rho}{a_{d}^{2}*f_{ck}*\left( -0.085*a_{d}^{2}*\left( s1 - 1.207 \right)+ a_{d}+ f_{ck}- 0.085*s2 \right)+ 0.762*f_{ck}} \right)^{0.351}$

Error: 0.163 $\frac{rho^{0.279}}{{0.950}^{s1 + s2}+ a_{d}*f_{ck}^{0.713}+ 0.060*a_{d}^{a_{d}- 0.414*f_{ck}}*f_{ck}}$

Error: 0.161 $\frac{\frac{{0.557}^{ad}}{{0.978}^{s2}}+ 0.012*ad*s1 + 0.014}{\left( \frac{f_{c}^{2}+ 0.391}{rho} \right)^{0.288}}$

Error: 0.166 $\left( \left( f_{c}+ 0.629 \right)*\frac{rho + \left( ad + rho \right)*\left( ad^{2}*f_{c}+ f_{c}- 0.020*s1 - 0.020*s2 \right)+ 0.177}{rho} \right)^{-0.330}$

Error: 0.164 $\frac{rho}{\left( rho*\left( ad + f_{c}*\left( -0.072*ad*\left( ad*\left( s2 - 1.904 \right)+ s1 \right)+ ad + 0.531 \right) \right) \right)^{0.719}}$

Error: 0.162 $\left( rho*\frac{rho*\frac{s1 + s2}{f_{c}+ rho}+\frac{0.405}{f_{c}+ 0.586}}{ad*f_{c}*\left( ad^{2}+ 0.998 \right)} \right)^{0.299}$

Error: 0.161 $\frac{rho}{rho + \left( rho*\left( 0.404*f_{c}+ rho \right)*\left( ad*\left( ad - 0.058*s1 - 0.058*s2 \right)+ ad + 0.692 \right) \right)^{0.622}}$

PC['vert']=PC['rho_v']*PC['fyv'];PC['horz']=PC['rho_h']*PC['fyh']

PC['vn']=PC['V']/(PC['h'] * PC['b'])*1000

PC['f_c']=PC['f_c']/30.0

Error: 0.175 $2.210*ad^{-ad - 0.257}*f_{c}+ 70.071*rho + \left( 2*f_{c}*s1 + s2 \right)^{0.274}$

Error: 0.170 $\left( f_{c}+\frac{f_{c}+ rho*s1 + 1.159}{-0.111*ad - 0.098} \right)*\left( -13.607*rho*\left( 0.004*rho \right)^{rho}- 0.321 \right)$

Error: 0.174 $\frac{4.453*f_{c}+ 4.453*rho*\left( s1 + s2 \right)^{ad}}{ad + f_{c}^{0.311}- 0.172}+ 0.490 + 104.164*\frac{rho}{ad}$

Error: 0.172 $0.822*f_{c}+\frac{ad^{0.772}+ rho*\left( s1^{ad + 0.827}+ 122.038 \right)}{ad + rho*s1}+\frac{f_{c}}{ad^{ad}}$

Error: 0.171 $\left( ad*\left( -0.047*\frac{f_{c}}{rho}+ s1 + s2 \right)+ 36.658*f_{c} \right)^{-0.125*ad + 2.979*rho + 0.554}- 0.571$

Error: 0.170 $\left( f_{c}*\frac{rho}{ad + rho} \right)^{2*ad*rho + 0.494}*\left( s1 + 35.488 \right)+ 0.792$

Error: 0.171 ${1.102}^{s1}+ f_{c}+\frac{f_{c}}{\left( ad^{2} \right)^{ad}}+ rho*\left( 4.213*s2 +\frac{94.423}{ad} \right)$

Error: 0.164 $8.417*rho^{0.299}*\left( ad^{-ad - 0.299}*f_{c}^{0.804}+ f_{c}^{0.136*ad}+ 0.024*s1^{ad} \right)$

Error: 0.166 $8.417*rho^{0.299}*\left( ad^{-ad - 0.299}*f_{c}^{0.804}+ f_{c}^{0.136*ad}+ 0.024*s1 \right)$

Error: 0.163 $rho^{ad*rho + 0.326}*\left( \left( ad*s1 + 0.214*s2 \right)^{0.594}+\frac{f_{c}+ 1.144}{0.067*ad + 0.047} \right)$

Error: 0.176 $\left( \frac{3*f_{c}+ rho*\left( ad*s1^{2}+ 171 \right)}{ad + f_{c}*rho + 0.295}+ 0.692 \right)^{0.946}$

Error: 0.160 $f_{c}*\left( ad^{2} \right)^{-ad + 0.107*s1}+ f_{c}+ \left( s2 +\frac{s1^{2}}{ad} \right)^{0.174}+ 99.791*\frac{rho}{ad}$

Error: 0.168 $f_{c}*\left( ad^{2} \right)^{-ad + 0.107*s1}+ f_{c}+ \left( s1 + s2 \right)^{0.174}+ 99.791*\frac{rho}{ad}$

Error: 0.173 $\left( \frac{ad}{rho} \right)^{rho*s1}+\frac{2*f_{c}+ rho*\left( s2 + 103.103 \right)}{ad + rho^{ad}}$

Error: 0.171 $f_{c}+ \left( rho*\left( s1*\left( s1 + s2 \right)+\frac{174.962}{ad} \right) \right)^{0.748}+ 0.610 +\frac{f_{c}}{ad^{1.652*ad}}$

Error: 0.172 $f_{c}^{0.468}*\left( 7.797*{0.517}^{ad}+ 15.595*rho^{0.517}+ 7.797*rho*s1 - 1.137 \right)$

Error: 0.175 ${1.035}^{s2}+\frac{f_{c}}{ad^{1.151}}+ 0.081*ad*s1 + f_{c}+ 103.900*\frac{rho}{ad^{ad}}$

Error: 0.162 $24.223*\left( 0.001*s1 + 0.001*s2 + f_{c}*\frac{rho}{ad} \right)^{0.462}+\frac{f_{c}}{ad^{1.462*ad}}$

PC['vert']=PC['rho_v']*PC['fyv'];PC['horz']=PC['rho_h']*PC['fyh']

PC['vn']=PC['V']/(PC['h'] * PC['b'])*1000/PC['f_c']

PC['f_c']=PC['f_c']/30.0

Error: 0.161 ${0.787}^{ad}*\frac{{1.017}^{s1 +\frac{s2}{f_{c}}}- 0.030}{\left( ad*f_{c}*\frac{f_{c}+ 1.042}{rho} \right)^{0.325}}$

Error:0.163 $\frac{{0.530}^{ad}}{\left( rho^{-0.197}-0.386 \right)*\left( f_{c}- 0.070 + \left( s1 + s2-0.197*f_{c} \right)^{-0.115*ad} \right)}+ rho$

Error: 0.164 $\frac{rho^{0.283}}{{0.930}^{s1 + s2}+ \left( ad + 0.052*f_{c} \right)*\left( ad*rho + f_{c} \right)^{0.804}}$

Error: 0.164 $\frac{rho^{0.296}}{{0.928}^{s1 + s2 + 1.095}+ f_{c}^{0.796}*\left( ad + 0.037*f_{c} \right)}$

Error: 0.159 $\frac{rho^{0.253}}{{1.218}^{ad - 0.306*s2}+ f_{c}*\left( ad + 0.091 \right)}+ 0.006*s1 - 0.003$

Error: 0.163 $\frac{rho^{0.301}}{0.981*ad*f_{c}^{0.836}+ rho + \left( s1 + s2 + 0.760 \right)^{-0.250}}$

Error: 0.163 $\left( f_{c}*\frac{ad^{2}*\left( ad*f_{c}+ ad + 2.338*f_{c}- 0.245*s1 - 0.245*s2 \right)+ 1.279}{rho} \right)^{-0.312}$

Error: 0.162 $\left( f_{c}*\left( ad*\left( ad - 0.302*s1 \right)+ 2.993 \right)*\frac{ad^{2}*f_{c}+ 0.352}{rho} \right)^{-0.314}$

Error: 0.161 $\left( \frac{rho}{f_{c}*\left( ad^{2}*\left( 1.358*ad*\left( 2*rho - 0.132 \right)*\left( s1 + s2 \right)+ ad + 1.358*f_{c} \right)+ 0.737 \right)} \right)^{0.347}$

Error: 0.157 $\left( f_{c}*\left( ad^{2}+ 0.821 \right)*\frac{1.756*ad*f_{c}+\frac{1.275}{s1 + s2}}{rho} \right)^{-0.321}$

$$\left( \frac{rho}{f_{c}*\left( ad^{2}+ 0.821 \right)\left( 1.756*\frac{a}{d}*f_{c}+\frac{1.275}{s1 + s2} \right)} \right)^{0.321}$$

Error: 0.164 $\frac{0.322}{\left( 0.044*ad*f_{c}*\frac{ad*\left( ad - 0.127*\left( s1+s2 \right) \right)+ f_{c}}{rho}+ 1 \right)^{0.363}}$

Error: 0.157 $\frac{0.815}{\left( f_{c}*\frac{ad*\left( ad*f_{c}*\left( 0.611*ad + 2*\frac{ad}{s1 + s2} \right)+ f_{c} \right)+ ad}{rho} \right)^{0.296}}$ $\frac{0.815}{\left( f_{c}*\frac{ad}{rho}*\left( \left( ad^{2}*f_{c}^{2}*\left[ \left( 0.611 + \frac{2}{s1 + s2} \right)+ 1 \right] \right)+ 1 \right) \right)^{0.296}}$

Error: 0.161 $\frac{rho^{0.286}}{{0.947}^{s1 + 1.170*s2}+ f_{c}^{-0.016*ad*f_{c}*s1 + 0.917}*\left( ad + f_{c}*rho \right)}$

Error: 0.164 $\left( \frac{rho}{ad*\left( ad*f_{c}*\left( ad+1.549 \right)+ad*\left( -0.100*s1-0.100*s2 \right) \right)*\left( f_{c}+rho+0.357 \right)+ f_{c}} \right)^{0.322}$

Error: 0.163 $\frac{{0.789}^{ad}}{\left( ad*\frac{f_{c}^{2}+ f_{c}- 0.098*s1 - 0.098*s2 + 0.198}{rho}+ s1 + s2 \right)^{0.326}}$

Error: 0.162 $\frac{{0.772}^{ad}}{\left( ad*f_{c}*\left( ad +\frac{-0.093*ad*\left( s1 + s2 \right)+ f_{c}+ 1.169}{rho} \right) \right)^{0.324}}$

Error: 0.165 $\left( \frac{rho}{f_{c}*\left( ad*\left( 1.920*ad*\left( 1.466*ad*f_{c}- 0.133*s2 \right)+ f_{c} \right)+ ad + 0.548 \right)} \right)^{0.312}$

Error: 0.163 $\frac{rho^{0.272}}{{1.156}^{ad*\left( -0.580*f_{c}*s1 - 0.580*s2 \right)}+ \left( ad*\left( 0.140*ad + f_{c} \right) \right)^{0.953}}$

Error: 0.164 $\frac{rho^{0.287}}{ad*f_{c}^{0.846}+ \left( f_{c}*rho + 0.872 \right)^{-0.231*f_{c}+ 0.559*s1 + 0.559*s2}}$

Error: 0.157 $\frac{0.009*f_{c}^{ad - 0.115*s2}*s1 + rho^{0.153}+ 0.009*s2 - 0.267}{ad*f_{c}+ 0.758}$

Error: 0.157 $\frac{rho^{0.309}}{ad*f_{c}^{0.879}+rho+0.726}+0.006*s1 - 0.004 + 0.006*\frac{0.602*s2 - 0.744}{f_{c}^{ad}}$

Error: 0.163 $\frac{rho^{0.275}}{{0.933}^{ad*\left( s1 + s2 \right)}+ 0.096*ad^{2}+ ad*f_{c}^{0.912}}$

Error: 0.163 $\left( \frac{rho}{ad^{2}*f_{c}*\left( ad*\left( f_{c}- 0.139*s1 - 0.139*s2 \right)+ ad + f_{c} \right)+ f_{c}} \right)^{0.326}$

Error: 0.164 $\left( \frac{rho}{-0.091*ad*s2 - 0.045*s1 + \left( ad*f_{c}^{2}+ f_{c} \right)*\left( ad^{2}+ rho + 0.600 \right)} \right)^{0.339}$

Error: 0.159 $rho^{0.286}*\frac{\left( f_{c}-0.190*ad \right)^{rho*ad*\left( f_{c}+1 \right)}}{ad*f_{c}+ \left( s1 + s2 \right)^{-0.190}}$

Error: 0.160 $\left( \left( ad*f_{c}+ 0.706 \right)*\frac{1.231*ad^{2}*\left( -0.109*f_{c}*s1 + 2*f_{c}- 0.109*s2 \right)+ f_{c}}{rho} \right)^{-0.309}$

Error: 0.167 $\left( \frac{rho}{ad*f_{c}*\left( ad*\left( ad*\left( -0.168*ad*s2 + 2*f_{c} \right)+ ad \right)+ f_{c} \right)+ f_{c}} \right)^{0.313}$

Error: 0.164 $\frac{rho^{0.292}}{{0.934}^{s1 + s2}+ ad*\left( {0.954}^{ad*f_{c}}*f_{c}+ ad^{2}*rho \right)}$

Error: 0.160 $\frac{rho^{0.292}}{ad*\left( ad*rho + f_{c}^{0.836} \right)+ f_{c}*rho^{ad + 0.241}+ \left( s1 + s2 + 0.361 \right)^{-0.254}}$

Error: 0.161 $\left( \frac{rho}{\left( f_{c}+ rho \right)*\left( ad*\left( ad - 0.138 \right)*\left( f_{c}+ 0.381 \right)+ 0.381 \right)} \right)^{0.381 +\frac{rho}{f_{c}*\left( s1 + s2 \right)}}$

Error: 0.162 $\left( \frac{rho}{ad*f_{c}*\left( f_{c}+ \left( ad - 0.159 \right)*\left( ad - 0.165*s1 \right)*\left( f_{c}+ 0.297 \right)+ 1.255 \right)} \right)^{0.342}$

Error:0.169 $1.093*{0.696}^{ad}*\left( \left( \left( f_{c}*\left( ad + f_{c} \right)+\frac{ad + 0.591}{rho} \right)*\left( f_{c}+ 1.420*rho - 0.417 \right) \right)^{-0.104}- 0.431 \right)$

PC['vn']=PC['V']/(PC['h'] * PC['b'])/PC['f_c']*1000

features=['ad','f_c','rho']

Error: 0.199 $\frac{rho^{0.174*ad - 2*rho}}{f_{c}^{0.397}}$

Error: 0.198 $(ad + 0.8)*\frac{f_{c}^{-0.256}- 0.157}{-0.157 + rho^{-0.291*ad}}$

Error: 0.208 $ad^{0.923}*\left( rho + \left( f_{c}- 1.874 +\frac{0.264}{ad*rho} \right)^{-0.541*ad} \right)$

Error: 0.190 $4.832*\frac{\left( ad*\frac{rho}{f_{c}} \right)^{0.295}}{ad*\left( rho + 2 \right) + 0.416}- 0.067$

Error: 0.193 $\frac{1.072}{\left( ad^{2}*\frac{f_{c}}{rho}+ 2*f_{c}^{2}*rho \right)^{0.097}}- 0.371$

Error: 0.186 $-0.005*f_{c}*rho + 3.167*\left( \frac{f_{c}}{rho} \right)^{-0.096*ad - 0.309}\to3.167\left( \frac{rho}{fc} \right)^{0.096a_{d}+0.309}-0.005f_{c}*rho$

Error: 0.189 $\frac{0.531}{\left( 0.604*ad*\left( 0.047*f_{c}+\frac{0.024}{rho} \right)+ 1 \right)^{1.256}}$

PC['vn']=PC['V']/(PC['h'] * PC['b'])/PC['f_c']*1000

features=['ad','f_c','rho']

Error: 0.191 $1.082*\left( \frac{rho}{ad*f_{c}*\left( ad + 0.110*f_{c}*rho \right)+ 0.983} \right)^{0.113}- 0.314$

Error: 0.193 $rho*\frac{\left( 0.070*f_{c}*rho + 0.029 \right)^{1.277*ad*rho - 0.912}}{ad + 0.257}$

Error: 0.193 $rho^{0.299}*\left( rho + \left( 0.230*f_{c} \right)^{-0.385*ad} \right)$

Error: 0.193 $\left( 0.629*ad^{2}*\frac{f_{c}}{rho}+ f_{c} \right)^{-0.131}- 0.250$

Error: 0.194 $\left( f_{c}+ 0.513*\frac{ad^{2}*f_{c}+ 1.430}{rho} \right)^{-0.115}- 0.307$

Error: 0.190 $\left( \frac{rho}{ad^{2}*f_{c}*\left( f_{c}*rho^{2}+ 0.301 \right)+ 1.056} \right)^{0.170}- 0.186$

Error: 0.193 $1.531*\frac{rho^{0.392}}{\left( 0.261*f_{c} \right)^{0.358*ad}}$

Error: 0.199 $\frac{f_{c}^{rho}+ 1.650*rho^{0.426*ad}*\left( ad*f_{c} \right)^{0.722}}{f_{c}}$

Error: 0.195 $\left( \frac{rho}{ad + rho} \right)^{0.415}*\left( {0.988}^{ad*f_{c}}+ 0.130 \right)$

Error: 0.192 $\left( \frac{rho}{0.158*ad*f_{c}*\left( ad + rho \right)+ 0.402} \right)^{0.247}- 0.097$

Error: 0.188 $\left( \frac{rho}{f_{c}*\left( ad^{2}+ 0.144 \right)*\left( rho + 0.129 \right)+ rho - 0.172} \right)^{0.244}- 0.102$

Error: 0.194 $\left( \frac{rho}{1.382*ad^{2}*f_{c}*\left( rho + 0.204 \right)+ 1.012} \right)^{0.105}- 0.362$

Error: 0.193 $\left( 0.492*ad^{2}*\frac{f_{c}}{rho}+ f_{c}*\left( f_{c}*rho - 0.415 \right) \right)^{-0.096}- 0.377$

Error: 0.188 $\frac{1.244}{\left( ad*\left( ad + f_{c}+\frac{rho + 0.552}{rho} \right) \right)^{0.303}}- 0.2$

Error: 0.188 $\frac{1.244}{\left( ad*\left( ad + f_{c}+\frac{rho + 0.552}{rho} \right) \right)^{0.303}}- 0.2$

Error: 0.208 $\frac{1}{f_{c}^{0.249}*\left( ad + 1.046 \right)}+ rho^{2.097*rho + 0.391}- 0.236$

Error: 0.195 $1.084*\left( \frac{rho}{ad*f_{c}*\left( ad + rho \right)+ 1.945} \right)^{0.114}- 0.311$

Error: 0.194 $1.615*\frac{rho^{0.361}}{{1.013}_{c}^{f}*ad^{1.043}+ 0.742}- 0.014$

Error: 0.201 $rho^{f_{c}*rho^{2}+ 0.619}- 0.054 +\frac{0.280}{\left( 0.256*f_{c} \right)^{0.413*ad}}$

Error: 0.186 $2.976*\frac{rho^{0.489}}{f_{c}^{0.190*ad + 1.190*rho + 0.142}- 0.623}$

Error: 0.194 $\frac{1.050}{\left( ad^{2}*\frac{f_{c}}{rho} \right)^{0.108}}- 0.322$

Error: 0.191 $\frac{1.195}{\left( 0.076*ad*\left( ad*f_{c}+\frac{1.145}{rho} \right)+ 0.076*f_{c}+ 1 \right)^{0.863}}$

Error: 0.194 $0.444*\left( 0.316*f_{c}^{2} \right)^{-0.122*ad -\frac{0.001}{rho}}$

Error: 0.192 $\left( \frac{rho}{ad*\left( 0.136*ad + rho \right)*\left( ad + f_{c} \right)+ 0.596} \right)^{0.218}- 0.136$

Error: 0.206 $\left( ad*\left( ad*\left( f_{c}- 10.894 \right)+ 2.374 +\frac{0.320}{rho} \right) \right)^{2*rho - 0.554}$

Error: 0.209 $\left( ad + rho \right)*\left( rho + \left( 0.619*f_{c}+\frac{0.164}{ad*rho} \right)^{-0.614*ad} \right)$

Error: 0.186 $\left( 0.193*f_{c}+ 0.322 +\frac{0.109}{rho} \right)^{-0.277*ad - 0.463}$

Error: 0.186 $\left( 0.193*f_{c}+ 0.322 +\frac{0.109}{rho} \right)^{-0.277*ad - 0.463}$

Error: 0.187 $\left( 0.193*f_{c}+ 15.137*rho +\frac{0.109}{rho} \right)^{-0.277*ad - 0.463}$

Error: 0.187 $\left( 0.193*f_{c}+ 15.137*rho + 0.090 +\frac{0.109}{rho} \right)^{-0.277*ad - 0.463}$

Error: 0.187 $\left( 0.193*f_{c}+ 15.137*rho + 0.074 +\frac{0.109}{rho} \right)^{-0.277*ad - 0.463}$

Error: 0.193 $2.860*\frac{rho^{0.180*ad + 0.186}}{f_{c}^{0.428}}$

Error: 0.193 $2.860*\frac{rho^{0.180*ad + 0.186}}{f_{c}^{0.428}}$

Error: 0.193 $\left( 0.453*ad^{2}*\frac{f_{c}}{rho}+ f_{c}^{2}*rho \right)^{-0.099}- 0.365$

Error: 0.195 $\left( \frac{rho}{0.492*ad^{2}*f_{c}+ 1.076} \right)^{0.133}- 0.256$

Error: 0.190 $1.222*\left( \frac{rho}{ad*\left( f_{c}*rho + 0.568 \right)} \right)^{0.289}- 0.217$

Error: 0.189 $1.222*\left( \frac{rho}{ad*\left( f_{c}*rho + 0.549 \right)} \right)^{0.289}- 0.217$

Error: 0.189 $1.222*\left( \frac{rho}{ad*\left( rho*\left( f_{c}+ 0.855 \right)+ 0.549 \right)} \right)^{0.289}- 0.217$

Error: 0.209 $\left( ad +\frac{rho}{ad^{2}} \right)*\left( rho + \left( f_{c}+\frac{0.187}{ad*rho} \right)^{-0.552*ad} \right)$

Error: 0.216 $\frac{ad}{ad + f_{c}^{0.653*ad}}+\frac{rho^{0.744}}{\left( f_{c}*rho \right)^{2*rho}}$

PC['vn']=PC['V']/(PC['h'] * PC['b'])/PC['f_c']*1000

Error: 0.201 $\frac{rho^{0.390}+ rho}{ad + rho + \left( 0.016*ad*\left( f_{c}- 2.287 \right) \right)^{ad}}$

Error: 0.196 $\frac{{0.574}^{\frac{0.012}{rho}}+ rho}{f_{c}^{0.431}*\left( ad + rho^{0.809*ad} \right)}$

Error: 0.192 $\left( \frac{rho}{ad^{2}*\left( f_{c}- 2.397 \right)+ 0.371} \right)^{0.128}- 0.244$

Error: 0.191 $2.137*ad*\frac{ad^{ad}}{\left( ad + f_{c}+\frac{0.529}{rho} \right)^{0.643*ad}}$

Error: 0.195 $\left( \frac{rho}{0.015*ad^{2}*f_{c}+ 0.157} \right)^{2*rho + 0.503}$

Error: 0.198 $\frac{\left( ad*rho \right)^{0.002*ad*f_{c}+ 0.343}}{\left( ad^{2}+ 0.356 \right)^{0.578}}$

Error: 0.213 $ad*rho^{0.573*ad + f_{c}*rho^{2}}+ f_{c}^{2.143*rho - 0.883}$

Error: 0.192 $\left( 0.656*ad^{2}*\frac{f_{c}}{rho}+ f_{c}^{2}*rho \right)^{-0.124}- 0.268$

Error: 0.188 $\left( 0.570*f_{c}*\left( 0.701 +\frac{0.149}{rho} \right) \right)^{-0.141*ad - 0.234}$

Error: 0.185 $1.657*\left( \frac{rho}{\left( f_{c}+ 3.098 \right)*\left( rho + 0.078 \right)} \right)^{0.140*ad + 0.319}$

Error: 0.193 $1.572*\frac{rho^{0.412}}{\left( \left( ad + f_{c} \right)*\left( rho + 0.192 \right) \right)^{0.366*ad}}$

Error: 0.197 $\left( ad*\frac{0.245*ad*f_{c}+ 1.849}{rho}+ f_{c} \right)^{-0.152}- 0.232$

Error: 0.196 $\frac{ad^{0.714}}{\left( \frac{f_{c}}{2*ad^{2}*rho} \right)^{0.286*ad}}$

Error: 0.193 $\left( ad^{2}*\frac{f_{c}}{2*rho}+ f_{c}^{2}*rho \right)^{-0.099}- 0.362$

Error: 0.210 $\left( ad + rho \right)*\left( rho + \left( f_{c}- 0.956 +\frac{0.175}{ad*rho} \right)^{-0.557*ad} \right)$

Error: 0.196 $\left( \frac{rho}{\left( 1.266*ad + rho \right)*\left( ad*\left( f_{c}+ rho \right)- 2.323 \right)} \right)^{0.154}- 0.162$

Error: 0.196 $\frac{ad}{\left( 0.464*f_{c}+\frac{0.124}{ad*rho} \right)^{0.661*ad}}+ rho$

Error: 0.189 $\frac{1.229}{\left( ad*\left( f_{c}+ 3.884 +\frac{0.532}{rho} \right) \right)^{0.282}}- 0.227$

Error: 0.192 $3.462*f_{c}^{-rho - 0.404}*rho^{0.179*ad + 0.240}$

Error: 0.195 $\left( \frac{rho}{ad^{2}*f_{c}*\left( rho + 0.367 \right)+ 1.257} \right)^{0.118}- 0.309$

Error: 0.194 $\left( 0.584*ad^{2}*\frac{f_{c}}{rho}+ f_{c} \right)^{-0.110}- 0.318$

Error: 0.200 $\frac{1}{{1.032}^{\frac{0.231}{rho}}*f_{c}^{0.411}*\left( ad + \left( 2*rho \right)^{ad} \right)}$

Error: 0.196 $\left( \frac{rho}{0.423*ad^{2}*f_{c}+ 0.474} \right)^{0.098}- 0.373$

Error: 0.187 $\frac{0.391}{\left( 0.012*ad*\left( f_{c}+\frac{0.552}{rho} \right)+ 1 \right)^{1.675}}$

Error: 0.187 $3.662*\left( 4.820*f_{c}+\frac{f_{c}}{rho} \right)^{-0.101*ad - 0.322}$

Error: 0.194 $\left( \frac{0.595*ad^{2}*f_{c}+ 0.692}{rho} \right)^{-0.137}- 0.236$

Error: 0.193 $\frac{1.089}{\left( ad*f_{c}*\left( \frac{ad}{rho}+ f_{c}*rho \right) \right)^{0.084}}- 0.434$

Error: 0.197 $\left( f_{c}*\left( 0.022*\frac{ad^{2}}{rho}+ 0.294 \right) \right)^{-0.493}$

Error: 0.218 $ad*\left( rho + \left( f_{c}+\frac{0.124}{rho} \right)^{-0.560*ad} \right)$

Error: 0.212 $ad*\left( rho + \left( f_{c}+\frac{0.150}{ad*rho} \right)^{-0.557*ad} \right)$

Error: 0.188 $\left( ad*\left( 0.405*f_{c}+ 1.267 +\frac{0.218}{rho} \right) \right)^{-0.348}- 0.168$

Error: 0.196 $\frac{-0.021*ad + \left( f_{c}*rho - 0.082 \right)^{0.310}}{0.131*ad*f_{c}+ 1.064}$

Error: 0.189 $rho*\frac{-0.165*ad + \left( rho*\left( f_{c}+\frac{f_{c}}{ad} \right) \right)^{-0.353}}{0.077*ad + rho}$

Error: 0.195 $4.060*\left( -0.000015 +\frac{rho}{ad*f_{c}*\left( ad + 0.273 \right)} \right)^{0.429}$

Error: 0.193 $\left( \frac{rho}{ad^{2}*f_{c}} \right)^{0.132}- 0.229$

Error: 0.193 $\left( \frac{rho}{0.799*ad^{2}*f_{c}+ 0.440} \right)^{0.123}- 0.264$

Error: 0.189 $\left( \frac{rho}{ad*\left( rho*\left( 0.355*f_{c}+ 1.333 \right)+ 0.193 \right)} \right)^{0.379}- 0.150$

$$\left( a_{d}*\left( 106.7*a_{d}*rho*\left( -2.14*a_{d}+ s2 +\frac{fck}{a_{d}} \right)+ 2.177302*a_{d}+2.18*s1 \right) \right)^{0.39}$$

$a_{d}^{0.36}*\left( \mathrm{rho}^{0.688}*\left( a_{d}*s2 + f\_c + 2*s1 -\frac{0.137239827231015}{\mathrm{rho}} \right)+ 3.1736393 \right)$ 0.1776

$$6.497*\left( a_{d}*rho*\left( -159.49*a_{d}*rho + a_{d}*s1 + f\_c + s2 -\frac{1.129634}{a_{d}} \right) \right)^{0.375}$$

$$\left( a_{d}*\left( 1.48*a_{d}*s1 + rho*\left( -81.35*a_{d}^{2} + 55.9011690004935*f\_c - 116.08 \right) \right) \right)^{0.4408813}+ 0.63$$

$$6.39*\left( a_{d}*rho*\left( 0.0763*a_{d}*f\_c*\left( s1 - 1.827 \right)+ f\_c + s2 - 1.484 \right) \right)^{0.366}+ 0.0336$$

$0.94*a_{d}^{0.994}+ a_{d}^{\frac{0.91}{a_{d}}}+ \mathrm{rho}^{0.68}*\left( a_{d}*1.508*\left( s1 + s2 \right)+ f\_c \right)+ 0.713$ 0.1879

$$0.156*a_{d}*s1 + 6.3985*\left( a_{d}*rho*\left( f\_c + 1.05*s2 - 7.76 \right) \right)^{0.369}+ 0.156$$

$$\frac{\left( rho*\left( a_{d}*\left( a_{d}*s1 + fck + s2 \right)- 0.58 \right) \right)^{1.32*rho + 0.438}}{a_{d}*rho + 0.13} error 0.1635$$

$6.1*\left( \left( a_{d}*\left( -1.6*a_{d}+ f\_c \right)- 0.14 \right)*\left( rho + 0.001*s1*\left( a_{d}+ 0.75 \right)+ 0.001*s2 + 4.97*{10}^{5} \right)- 0.0084 \right)^{0.39}$

$6.34*\left( rho*\left( a_{d}*\left( a_{d}*\left( -1.53*a_{d}+ s1 \right)+ f\_c \right)- 5.25 \right)+ 0.022 \right)^{0.387}+ 0.325$ error 0.1699

$6.32*\left( 0.022*a_{d}^{2}*\left( s1 +s2 \right)+ a_{d}*rho*\left( -1.82*a_{d}^{2}+ f\_c - 1.58 \right) \right)^{0.39}$ error 0.1616

$$a_{d}+ 1.598*\frac{\left( rho*\left( 1.56*a_{d}*s1 + 1.17*f\_c \right) \right)^{0.676}}{rho + \left( 1.17*rho \right)_{d}^{a}+ 0.345}+ 0.84$$

$$\left( a_{d}*\left( 135.75*rho + 0.549 \right)*\left( -2.18*a_{d}+ fck + 1.858*s1 + 1.37*s2 - 1.76 \right) \right)^{0.377}- 0.48$$

$$6.495*\left( a_{d}*rho*\left( a_{d}^{2}*\left( s1 - 2.48 \right)+ f\_c + s2 -\frac{2.48}{a_{d}} \right) \right)^{0.36}+ 0.054$$

PC['vert']=PC['rho_v']*PC['fyv'];PC['horz']=PC['rho_h']*PC['fyh']

PC['vn']=PC['V']/(PC['h'] * PC['b'])*1000/PC['f_c']

PC['f_c']=PC['f_c']/30.0

PC['ss']=PC['vert']*2+PC['horz']

PC['s1']=PC['vert']/PC['f_c'];PC['s2']=PC['horz']/PC['f_c']

(2*rho/(f_c*(ad^2 - 0.039*ad*(ad*s1 + s2) + 0.54)))^(2.549*rho + 0.42) Error: 0.167

1.017^(1.592216*ad*f_c*s1 + s2)*rho^0.286/(1.12^(ad*(ad + 0.282)) + 0.803*ad*f_c) Error: 0.1608

Error: 0.1617 0.758^ad/(ad*f_c*(ad*(-2.0*s1 - 2.9*s2 + 5.8) + (f_c + 0.72)/rho))^0.32553884

Error: 0.15775887410155323 0.99275345^(f_c*s2)/(0.38260987^ad + ad*f_c*((f_c*rho*(s1 + s2 + 2.1271954))^(-0.44292453) + 0.40296805) + 2.1271954)

Error: 0.16006603259010496 (rho/(ad*(f_c*(ad*(0.11934049*ad + f_c)*(ad - 0.21518488*s1 - 0.21518488*s2 + 1.0967479) + f_c) + 1.0967479)))^0.32876948

Mean Squared Error: 0.1616960958265315 (ad*(f_c*(ad*(f_c + 0.45751578)*(ad - 0.10517342*s1 - 0.10517342*s2) + 1.3115842*f_c) + 0.93340546)/rho)^(-0.3329309)

Mean Squared Error: 0.16388543507052541 rho^0.27682614/(0.9327286^(s1 + s2 + 1.4772595) + f_c^(rho + 0.64428164)*(1.1987364*ad + rho^(1.3292648*ad)))

Error: 0.16377762670055404 ((ad + 0.47940058)*(ad*f_c*(f_c + 0.19041009)*(ad - 0.07335686*s1 - 0.07335686*s2) + f_c)/rho)^(-0.34569216)

Error: 0.16049932396878444 (rho/(ad^2*f_c*(f_c + (f_c + 0.91211253)*(ad*(-0.08672052*f_c*s1 - 0.08672052*s2) + ad)) + f_c))^0.33197397

Error: 0.15871600688001788 (rho + 0.89259046687749*(f_c^2*rho)^0.2651595)/(0.98223317^(2.3854148*s1 + 2.3854148*s2)*ad^1.2831234*f_c + f_c)

Error: 0.16362429709103654 rho^0.2895358/(0.92319286^(0.73782676*s1 + 0.73782676*s2) + ad*(ad*rho*(ad - 1.2034018) + f_c^0.73782676))

Error: 0.16535466639556712 rho^0.27921093/(0.9403078^(f_c*(ad + s1 + s2 - 1.8202094)) + 1.0231358^(ad^2)*ad*f_c^0.8198478)

Error: 0.16165156721510698 rho^0.26750705/(0.98282707^s1 + 1.08937387736595*ad*(0.14292416*ad + f_c)/(ad*f_c + s1 + s2)^0.16783817)

Error: 0.16595943600361615 0.853327030249253*0.686147330633142^ad/(0.6062143*f_c*(0.85100704*ad*f_c + 0.5187557)/rho - s2)^0.31689417

Error: 0.1630347232183897 (rho/(ad*(ad + f_c*(ad^2*f_c*(-0.25154865*s1 - 0.25154865*s2 + 2.6116545) + f_c)) + f_c))^0.31065893

PC['vn']=PC['V']/(PC['h'] * PC['b'])*1000/PC['f_c']

PC['f_c']=PC['f_c']/30.0

PC['ss']=PC['vert']*2+PC['horz']

PC['s1']=PC['vert']/PC['f_c'];PC['s2']=PC['horz']/PC['f_c']

features=['ad','f_c','rho','s1','s2']

X=PC[features];y=PC['vn']

Mean Squared Error: 0.1652029357777135 rho^0.27729765/(0.88535154^(0.52552974*f_c*(s1 + s2 - 0.8710819)) + ad*(0.02775837*ad^2 + f_c)^0.855289030051107)

Mean Squared Error: 0.1678194565827842 (rho/(rho + (ad + rho)*(-0.0385834636045742*f_c*(s1 + s2) + f_c)))^(1.56166569826139*ad*rho + 0.4223947)

Error: 0.16308654298846184 (rho/(ad*f_c*(ad*(ad + f_c - 0.05864696*(ad + f_c)*(s1 + s2)) + f_c) + 0.374452))^0.34045708

Error: 0.16383618817499512 (rho/(ad*(-0.028174559*ad*f_c*(s1 + s2) + f_c)*(ad^2 + f_c + 0.6550527)))^0.3573858

Error: 0.16281573412596614 (rho/(ad*(f_c + 0.13434973)*(f_c + (ad + f_c)*(ad - 0.040739615*(ad + f_c)*(s1 + s2))) + 0.3378967))^0.3378967

Error: 0.1625437927857339 (rho/(ad^2*f_c*(-0.08473299*ad^2*(s1 - 1.2067732) + ad + f_c - 0.08473299*s2) + 0.7621819*f_c))^0.35065967

Error: 0.16305643353362 rho^0.27880418/(0.95001113^(s1 + s2) + ad*f_c^0.7133867 + 0.060425367*ad^(ad - 0.41357868383692*f_c)*f_c)

Error: 0.1614701592894475 (0.5565549^ad/0.9784725^s2 + 0.0115033817969284*ad*s1 + 0.01415956)/((f_c^2 + 0.39105052)/rho)^0.28757662

Error: 0.1660390671066667 ((f_c + 0.62852806)*(rho + (ad + rho)*(ad^2*f_c + f_c - 0.019894026*s1 - 0.019894026*s2) + 0.17679925)/rho)^(-0.33045933)

Error: 0.16446453478122425 rho/(rho*(ad + f_c*(-0.07226578*ad*(ad*(s2 - 1.9042596) + s1) + ad + 0.53136136808679)))^0.7187445

Error: 0.16175370977521084 (rho*(rho*(s1 + s2)/(f_c + rho) + 0.4048103/(f_c + 0.58628905))/(ad*f_c*(ad^2 + 0.99750566)))^0.2991804

Error: 0.16109246878584574 rho/(rho + (rho*(0.40383095*f_c + rho)*(ad*(ad - 0.057846162*s1 - 0.057846162*s2) + ad + 0.691948778))^0.6216529)

PC['vert']=PC['rho_v']*PC['fyv'];PC['horz']=PC['rho_h']*PC['fyh']

PC['vn']=PC['V']/(PC['h'] * PC['b'])*1000

PC['f_c']=PC['f_c']/30.0

Error: 0.1753173245064409 2.21020460306051*ad^(-ad - 0.25669122)*f_c + 70.0707706568631*rho + (2*f_c*s1 + s2)^0.27441838

Error: 0.17034498322888864 (f_c + (f_c + rho*s1 + 1.1594324)/(-0.11126381*ad - 0.097698003344893))*(-13.6070221346509*rho*(0.0037504428*rho)^rho - 0.3209166)

Error: 0.17416789707797295 (4.452615*f_c + 4.452615*rho*(s1 + s2)^ad)/(ad + f_c^0.31070423 - 0.17245609) + 0.49032852 + 104.164051714952*rho/ad

Error: 0.1722432309358823 0.822093263192336*f_c + (ad^0.7716831 + rho*(s1^(ad + 0.8274047) + 122.03838))/(ad + rho*s1) + f_c/ad^ad

Error: 0.17109391785501257 (ad*(-0.046756454*f_c/rho + s1 + s2) + 36.6577609080391*f_c)^(-0.12475116*ad + 2.97894218537522*rho + 0.55392593) - 0.57063466

Error: 0.16987139884239083 (f_c*rho/(ad + rho))^(2*ad*rho + 0.49391347)*(s1 + 35.4883986395978) + 0.7918104

Error: 0.1713409779763929 1.1019047^s1 + f_c + f_c/(ad^2)^ad + rho*(4.21324496754221*s2 + 94.4234694042693/ad)

Error: 0.1639172887659914 8.41699687948258*rho^0.29944125*(ad^(-ad - 0.29944125)*f_c^0.80441874 + f_c^(0.13627683*ad) + 0.023856888*s1^ad)

Error: 0.16560989093761377 8.41699687948258*rho^0.29944125*(ad^(-ad - 0.29944125)*f_c^0.80441874 + f_c^(0.13627683*ad) + 0.023856888*s1)

Error: 0.16315734928547385 rho^(ad*rho + 0.32571977)*((ad*s1 + 0.21442744*s2)^0.5936983 + (f_c + 1.1443319)/(0.06721839*ad + 0.046583244996426))

Error: 0.17643710702317672 ((3*f_c + rho*(ad*s1^2 + 170.99838))/(ad + f_c*rho + 0.294923467) + 0.69246405)^0.94631636

Error: 0.16018431739943537 f_c*(ad^2)^(-ad + 0.10718014*s1) + f_c + (s2 + s1^2/ad)^0.17401163 + 99.7907189043139*rho/ad

Error: 0.1678191135415467 f_c*(ad^2)^(-ad + 0.10718014*s1) + f_c + (s1 + s2)^0.17417115 + 99.7907189043139*rho/ad

Error: 0.17315474278066856 (ad/rho)^(rho*s1) + (2*f_c + rho*(s2 + 103.102554))/(ad + rho^ad)

Error: 0.1706631298613098 f_c + (rho*(s1*(s1 + s2) + 174.96242/ad))^0.7477423 + 0.60961527 + f_c/ad^(1.6522918*ad)

Error: 0.17154865186044047 f_c^0.4679759*(7.79730135400138*0.51670355^ad + 15.5946027080028*rho^0.51670355 + 7.79730135400138*rho*s1 - 1.1373924)

Error: 0.17466125993088122 1.0346096^s2 + f_c/ad^1.1511151 + 0.08131611*ad*s1 + f_c + 103.900011900108*rho/ad^ad

Error: 0.16176552969596572 24.2226959586025*(0.0010068334*s1 + 0.0010068334*s2 + f_c*rho/ad)^0.46186283 + f_c/ad^(1.46186283*ad)

PC['vert']=PC['rho_v']*PC['fyv'];PC['horz']=PC['rho_h']*PC['fyh']

PC['vn']=PC['V']/(PC['h'] * PC['b'])*1000/PC['f_c']

PC['f_c']=PC['f_c']/30.0

Error: 0.16140238592602435 0.78729343^ad*(1.0171463^(s1 + s2/f_c) - 0.030016629)/(ad*f_c*(f_c + 1.0422592)/rho)^0.32480577

Error: 0.16264270118107554 0.53018117^ad/((rho^(-0.19716683) - 0.38574344)*(f_c - 0.06985062 + (-0.19716683*f_c + s1 + s2)^(-0.115116335*ad))) + rho

Error: 0.16380774995285158 rho^0.28308782/(0.930042^(s1 + s2) + (ad + 0.051854270848491*f_c)*(ad*rho + f_c)^0.8036735)

Error: 0.16381330968040358 rho^0.2962329/(0.92803484^(s1 + s2 + 1.0945278) + f_c^0.795943335*(ad + 0.03725128*f_c))

Error: 0.15910181983286975 rho^0.25262484/(1.2184963^(ad - 0.30589262*s2) + f_c*(ad + 0.091101356)) + 0.005941891*s1 - 0.00254994029997766

Error: 0.16346946240352273 rho^0.300637/(0.98056674*ad*f_c^0.8358583 + rho + (s1 + s2 + 0.7595448)^(-0.24987414))

Error: 0.1634392622735554 (f_c*(ad^2*(ad*f_c + ad + 2.337942*f_c - 0.24452624219796*s1 - 0.24452624219796*s2) + 1.2787184)/rho)^(-0.311677)

Error: 0.1622262187073454 (f_c*(ad*(ad - 0.30196404*s1) + 2.9930253)*(ad^2*f_c + 0.35158855)/rho)^(-0.314316273924933)

Error: 0.16143899011516186 (rho/(f_c*(ad^2*(1.3579063*ad*(2*rho - 0.13184755)*(s1 + s2) + ad + 1.3579063*f_c) + 0.73683125)))^0.34706897

Error: 0.15673637202141089 (f_c*(ad^2 + 0.8212575)*(1.7556022*ad*f_c + 1.27464391701614/(s1 + s2))/rho)^(-0.32067218)

Error: 0.16407531537928421 0.322149029700879/(0.0442051097258268*ad*f_c*(ad*(ad - 0.12663691*s1 - 0.12663691*s2) + f_c)/rho + 1)^0.36318433

Error: 0.15698306682146948 0.814677076999706/(f_c*(ad*(ad*f_c*(0.610931768696345*ad + 2*ad/(s1 + s2)) + f_c) + ad)/rho)^0.29569978

Error: 0.1610103750699458 rho^0.28605306/(0.9473162^(s1 + 1.169921*s2) + f_c^(-0.01560928*ad*f_c*s1 + 0.91673315)*(ad + f_c*rho))

Error: 0.16407135199874961 (rho/(ad*(ad*f_c*(ad + 1.5487891) + ad*(-0.099813424*s1 - 0.099813424*s2))*(f_c + rho + 0.3567469) + f_c))^0.32241502

Error: 0.16285322253667847 0.788762^ad/(ad*(f_c^2 + f_c - 0.09810462*s1 - 0.09810462*s2 + 0.19802734)/rho + s1 + s2)^0.326019661775464

Error: 0.16206827931876444 0.77186346^ad/(ad*f_c*(ad + (-0.092942804*ad*(s1 + s2) + f_c + 1.1686008)/rho))^0.32373604

Error: 0.16502304088612388 (rho/(f_c*(ad*(1.9204354*ad*(1.4664217*ad*f_c - 0.13330439*s2) + f_c) + ad + 0.548243)))^0.312

Error: 0.16336624020940238 rho^0.27215633/(1.1563936^(ad*(-0.58049244*f_c*s1 - 0.58049244*s2)) + (ad*(0.14016183*ad + f_c))^0.9534871)

Error: 0.16389370324056532 rho^0.28719333/(ad*f_c^0.84595084 + (f_c*rho + 0.8719396)^(-0.23115161674746*f_c + 0.5594068*s1 + 0.5594068*s2))

Error: 0.15702238688285605 (0.00910711711545228*f_c^(ad - 0.115062832066206*s2)*s1 + rho^0.15301284 + 0.00910711711545228*s2 - 0.26657313)/(ad*f_c + 0.75761503)

Error: 0.15650643150700372 rho^0.3089876/(ad*f_c^0.87858176 + rho + 0.7256604) + 0.0058200816*s1 - 0.00361916437281173 + 0.0058200816*(0.6023688*s2 - 0.74396384)/f_c^ad

Error: 0.16278971628354402 rho^0.2751883/(0.9329289^(ad*(s1 + s2)) + 0.095795184*ad^2 + ad*f_c^0.9120516)

Error: 0.163424047574831 (rho/(ad^2*f_c*(ad*(f_c - 0.1386729*s1 - 0.1386729*s2) + ad + f_c) + f_c))^0.326348610550759

Error: 0.1643459175257892 (rho/(-0.090670846*ad*s2 - 0.045335423*s1 + (ad*f_c^2 + f_c)*(ad^2 + rho + 0.59992296)))^0.3390997

Error: 0.1589181876724612 rho^0.286067*(-0.19004355*ad + f_c)^(rho*(ad*f_c + ad))/(ad*f_c + (s1 + s2)^(-0.19004355))

Error: 0.16018978188915753 ((ad*f_c + 0.7059134)*(1.230749*ad^2*(-0.10947949*f_c*s1 + 2*f_c - 0.10947949*s2) + f_c)/rho)^(-0.30856508)

Error: 0.1674812680275955 (rho/(ad*f_c*(ad*(ad*(-0.16797642*ad*s2 + 2*f_c) + ad) + f_c) + f_c))^0.3125012

Error: 0.1638284628271944 rho^0.2924111/(0.93370634^(s1 + s2) + ad*(0.95442516^(ad*f_c)*f_c + ad^2*rho))

Error: 0.15997524858423187 rho^0.291906/(ad*(ad*rho + f_c^0.83636755) + f_c*rho^(ad + 0.24145067) + (s1 + s2 + 0.36065948)^(-0.25423622))

Error: 0.1609901537157504 (rho/((f_c + rho)*(ad*(ad - 0.13782041)*(f_c + 0.38091865) + 0.38091865)))^(0.38091865 + rho/(f_c*(s1 + s2)))

Error: 0.16198719594020938 (rho/(ad*f_c*(f_c + (ad - 0.15887646)*(ad - 0.1652256*s1)*(f_c + 0.29684803) + 1.2546856)))^0.34223622

Error: 0.16856698847186183 1.0932852*0.69564354^ad*(((f_c*(ad + f_c) + (ad + 0.591188)/rho)*(f_c + 1.41950162433571*rho - 0.4170695))^(-0.10375514) - 0.43068522)

PC['vn']=PC['V']/(PC['h'] * PC['b'])/PC['f_c']*1000

features=['ad','f_c','rho']

Error: 0.19864297620000154 rho^(0.173944*ad - 2*rho)/f_c^0.39691243

Error: 0.1979522708579545 (ad + 0.8003141)*(f_c^(-0.2557186) - 0.15669526)/(-0.15669526 + rho^(-0.29057407*ad))

Error: 0.20846088670765364 ad^0.9230947*(rho + (f_c - 1.8743323 + 0.26398525/(ad*rho))^(-0.54092884*ad))

Error: 0.19007399891062574 4.8318086*(ad*rho/f_c)^0.29541546/(ad*rho + 2*ad + 0.41571784) - 0.066898495

Error: 0.19317676931670838 1.0720665/(ad^2*f_c/rho + 2*f_c^2*rho)^0.09701975 - 0.37116364

Error: 0.18558992588713247 -0.0053056508736324*f_c*rho + 3.1674929*(f_c/rho)^(-0.0958634*ad - 0.30919015)

Error: 0.18907483095316688 0.530715491091671/(0.603983200352238*ad*(0.0466189*f_c + 0.0238449967958163/rho) + 1)^1.2564816

PC['vn']=PC['V']/(PC['h'] * PC['b'])/PC['f_c']*1000

features=['ad','f_c','rho']

Error: 0.19087420987055978 1.08213274126713*(rho/(ad*f_c*(ad + 0.11002313*f_c*rho) + 0.9828094))^0.112845406 - 0.31397173

Error: 0.1932658841624657 rho*(0.069979146*f_c*rho + 0.0291538496634413)^(1.2774279*ad*rho - 0.91230106)/(ad + 0.25745162)

Error: 0.1930164243583314 rho^0.2989391*(rho + (0.23047012*f_c)^(-0.3851998*ad))

Error: 0.19296412446222647 (0.628893044474373*ad^2*f_c/rho + f_c)^(-0.13141385) - 0.25022328

Error: 0.19438572036436172 (f_c + 0.51270664*(ad^2*f_c + 1.4304041)/rho)^(-0.11497319) - 0.30652204

Error: 0.1896446113929794 (rho/(ad^2*f_c*(f_c*rho^2 + 0.30110124) + 1.0562274))^0.17028008 - 0.18603688

Error: 0.1933336506235278 1.53118164031949*rho^0.39154142/(0.26068112*f_c)^(0.35791066*ad)

Error: 0.19940005887078863 (f_c^rho + 1.64967233655031*rho^(0.42563167*ad)*(ad*f_c)^0.7221795)/f_c

Error: 0.1948186067549963 (rho/(ad + rho))^0.4153753*(0.9875785^(ad*f_c) + 0.129918526)

Error: 0.1924519492988548 (rho/(0.1575473*ad*f_c*(ad + rho) + 0.4018111))^0.24651968 - 0.096628524

Error: 0.1876939987901253 (rho/(f_c*(ad^2 + 0.14431654)*(rho + 0.1293831) + rho - 0.17219193))^0.24431057 - 0.10239257

Error: 0.1942336090633044 (rho/(1.3817029*ad^2*f_c*(rho + 0.203979) + 1.01179334434388))^0.10507456 - 0.3622439

Error: 0.1931599581101996 (0.49164847*ad^2*f_c/rho + f_c*(f_c*rho - 0.41487166))^(-0.095534645) - 0.37655526

Error: 0.18849807007612587 1.2437377/(ad*(ad + f_c + (rho + 0.5520484)/rho))^0.3030311 - 0.2

Error: 0.18849807007612587 1.2437377/(ad*(ad + f_c + (rho + 0.5520484)/rho))^0.3030311 - 0.2

Error: 0.20819154594733794 1/(f_c^0.24948196*(ad + 1.0457006)) + rho^(2.09661062876693*rho + 0.39114395) - 0.236

Error: 0.19488083171653325 1.08371325392224*(rho/(ad*f_c*(ad + rho) + 1.9452975))^0.11408769 - 0.31064796

Error: 0.19389258055028 1.61490671340089*rho^0.36078218/(1.0134267^f_c*ad^1.0430878 + 0.7417303) - 0.01396161

Error: 0.20063344416249443 rho^(f_c*rho^2 + 0.61939853) - 0.054114286 + 0.27967897/(0.25574926*f_c)^(0.41327348*ad)

Error: 0.18634378687112682 2.9761903*rho^0.48943815/(f_c^(0.1896267*ad + 1.1896267*rho + 0.141572417582961) - 0.62322044)

d Error: 0.194364550243671 1.04970175300921/(ad^2*f_c/rho)^0.10799112 - 0.3216715

Error: 0.19109012279541931 1.19504279308134/(0.0764655587430395*ad*(ad*f_c + 1.1450098/rho) + 0.0764655587430395*f_c + 1)^0.86298347

Error: 0.1938168964795666 0.4439747775286*(0.316213723*f_c^2)^(-0.12194894*ad - 0.0011019728050434/rho)

Error: 0.19172579353785724 (rho/(ad*(0.1356318*ad + rho)*(ad + f_c) + 0.59598273))^0.21817505 - 0.13583188

Error: 0.2057366737120081 (ad*(ad*(f_c - 10.8939441552654) + 2.3744411 + 0.31975225/rho))^(2*rho - 0.55364245)

Error: 0.2089248930619706 (ad + rho)*(rho + (0.61925203*f_c + 0.16367619625222/(ad*rho))^(-0.61363554*ad))

i = 7 1.034263773697072 Mean Squared Error: 0.18613850700028634 (0.19282725*f_c + 0.321691290834375 + 0.109140694/rho)^(-0.27730215*ad - 0.46325356)

i = 8 1.0348870090010664 Mean Squared Error: 0.18670769181040459 (0.19282725*f_c + 15.1368255503561*rho + 0.109140694/rho)^(-0.27730215*ad - 0.46325356)

i = 9 1.0294765897534373 Mean Squared Error: 0.1869436387045423 (0.19282725*f_c + 15.1368255503561*rho + 0.0900929270743425 + 0.109140694/rho)^(-0.27730215*ad - 0.46325356)

i = 10 1.0304159304375355 Mean Squared Error: 0.1869008944147758 (0.19282725*f_c + 15.1368255503561*rho + 0.074364696685125 + 0.109140694/rho)^(-0.27730215*ad - 0.46325356)

Error: 0.19297641007566652 2.85965716*rho^(0.18044226*ad + 0.186151597460208)/f_c^0.42750025

Error: 0.19297641007566652 2.85965716*rho^(0.18044226*ad + 0.186151597460208)/f_c^0.42750025

Error: 0.19333824996740007 (0.45320743*ad^2*f_c/rho + f_c^2*rho)^(-0.09945931) - 0.36531252

i = 7 1.0390375402706593 Mean Squared Error: 0.1953558364284361 (rho/(0.4922368*ad^2*f_c + 1.0757453))^0.1329713 - 0.2556673

i = 8 1.016671109270561 Mean Squared Error: 0.1899269892604623 1.22157950271922*(rho/(ad*(f_c*rho + 0.5677589)))^0.28874776 - 0.217442918572093

i = 9 1.0320587528872536 Mean Squared Error: 0.18909276149419127 1.22157950271922*(rho/(ad*(f_c*rho + 0.548952113)))^0.28874776 - 0.217442918572093

i = 10 1.0214635232797438 Mean Squared Error: 0.18934711003410815 1.22157950271922*(rho/(ad*(rho*(f_c + 0.85505897) + 0.548952113)))^0.28874776 - 0.217442918572093

Error: 0.20934319725166464 (ad + rho/ad^2)*(rho + (f_c + 0.18732229/(ad*rho))^(-0.55155647*ad))

Error: 0.2160310878144535 ad/(ad + f_c^(0.65312815*ad)) + rho^0.74424666/(f_c*rho)^(2*rho)

PC['vn']=PC['V']/(PC['h'] * PC['b'])/PC['f_c']*1000

Error: 0.20070797107002217 (rho^0.38960296 + rho)/(ad + rho + (0.015781086*ad*(f_c - 2.2872898))^ad)

Error: 0.19636636524824752 (0.5742973^(0.01162973/rho) + rho)/(f_c^0.43130448*(ad + rho^(0.8092*ad)))

Error: 0.19223897050042682 (rho/(ad^2*(f_c - 2.3968972) + 0.37112538))^0.12764066 - 0.2437771

Error: 0.19122956492434243 2.137227*ad*ad^ad/(ad + f_c + 0.5291064/rho)^(0.643436879485559*ad)

Error: 0.19497584959947936 (rho/(0.014520158855293*ad^2*f_c + 0.15698631))^(2*rho + 0.50290704)

Error: 0.19756234367320782 (ad*rho)^(0.0024345245017185*ad*f_c + 0.3432943)/(ad^2 + 0.35572916)^0.5779847

Error: 0.2127465295835781 ad*rho^(0.572632277197084*ad + f_c*rho^2) + f_c^(2.1425557*rho - 0.883415)

Error: 0.19194158841122091 (0.6562589*ad^2*f_c/rho + f_c^2*rho)^(-0.124345556) - 0.2675521

Error: 0.18776306856812375 (0.5701629*f_c*(0.70095116 + 0.148729200574854/rho))^(-0.141047679626481*ad - 0.23447408)

Error: 0.18476902274708082 1.6569768*(rho/((f_c + 3.0975882)*(rho + 0.077995345)))^(0.14040595*ad + 0.3186759)

Error: 0.1925827446687029 1.57228499183245*rho^0.41191053/((ad + f_c)*(rho + 0.19171566))^(0.36565307*ad)

0.19685713341227096 (ad*(0.24499752*ad*f_c + 1.8489356)/rho + f_c)^(-0.15244147) - 0.23165385

Error: 0.1958600967521372 ad^0.7135758/(f_c/(2*ad^2*rho))^(0.286120462322599*ad)

Error: 0.19305335267182838 (ad^2*f_c/(2*rho) + f_c^2*rho)^(-0.099295646) - 0.3615062

Error: 0.20987306005870093 (ad + rho)*(rho + (f_c - 0.95638204 + 0.17458238/(ad*rho))^(-0.5569308*ad))

Error: 0.19582672500111717 (rho/((1.2659795*ad + rho)*(ad*(f_c + rho) - 2.3229945)))^0.15442708 - 0.16232435

Error: 0.19642376026481426 ad/(0.46363407*f_c + 0.123826978104749/(ad*rho))^(0.6608561*ad) + rho

Error: 0.1892645531955782 1.2285595/(ad*(f_c + 3.8844066 + 0.53199595/rho))^0.281689346410538 - 0.227070192714015

Error: 0.19168766258122344 3.46187470277642*f_c^(-rho - 0.40350634)*rho^(0.17861035*ad + 0.240170746923085)

rror: 0.1947761839495437 (rho/(ad^2*f_c*(rho + 0.36736637) + 1.2571145))^0.11828578 - 0.30858922

Error: 0.1936320306741344 (0.5841873*ad^2*f_c/rho + f_c)^(-0.10985942) - 0.318183109

Error: 0.1998009724347945 1/(1.0323564^(0.23121777/rho)*f_c^0.4112704*(ad + (2*rho)^ad))

error: 0.19580091224484414 (rho/(0.4229528*ad^2*f_c + 0.47407138))^0.098244675 - 0.3729295

Error: 0.18657981225841042 0.391498031373246/(0.0119761836996297*ad*(f_c + 0.5515856/rho) + 1)^1.6750098

Error: 0.18721516028203608 3.6617086*(4.81972261339626*f_c + f_c/rho)^(-0.1006359*ad - 0.321684)

Error: 0.18692079732049918 (f_c/rho)^(-0.101550356*ad - 0.3227756)*(3.766312995 - 9.84733130822308*rho)

i = 9 1.0318270341728315 Mean Squared Error: 0.18654777321568314 3.6617086*(4.81972261339626*f_c^2*rho + f_c/rho)^(-0.1006359*ad - 0.321684)

i = 10 1.027596710386325 Mean Squared Error: 0.1868289006895285 3.6617086*(5.44610203944276*f_c^2*rho + f_c/rho)^(-0.1006359*ad - 0.321684)

Error: 0.1937109015497254 ((0.5950323*ad^2*f_c + 0.69189397841997)/rho)^(-0.13733865) - 0.23558708

Error: 0.19348617179779476 1.08935294385919/(ad*f_c*(ad/rho + f_c*rho))^0.08417625 - 0.4341287

Error: 0.1967641937100679 (f_c*(0.022034662106467*ad^2/rho + 0.294146))^(-0.4929798)

Error: 0.21774736366501232 ad*(rho + (f_c + 0.12364918/rho)^(-0.560014567*ad))

Error: 0.21198035282343888 ad*(rho + (f_c + 0.150312690077067/(ad*rho))^(-0.55703396*ad))

Error: 0.1883099806475295 (ad*(0.40478942*f_c + 1.2673715 + 0.2179564/rho))^(-0.34784818) - 0.16780692

Error: 0.19597349726869615 (-0.020834757*ad + (f_c*rho - 0.081733376)^0.3098322)/(0.13146035*ad*f_c + 1.064169)

Error: 0.18885259410480465 rho*(-0.16549622*ad + (rho*(f_c + f_c/ad))^(-0.3525606))/(0.07689967*ad + rho)

Error: 0.1950711831707213 4.06006285688015*(-1.57515318265158e-5 + rho/(ad*f_c*(ad + 0.27347624)))^0.42941016

Error: 0.19303701043462423 (rho/(ad^2*f_c))^0.13182046 - 0.229351489862488

Error: 0.1934447756281957 (rho/(0.7987765*ad^2*f_c + 0.4395037643618))^0.12280926 - 0.2638372

Error: 0.1886983951844325 (rho/(ad*(rho*(0.3548754*f_c + 1.3328335) + 0.1929951)))^0.37850448 - 0.14985959
