# Supplementary material for: Prediction and reliability analysis of shear strength of RC deep beams
Source: Sci Rep. 2024 Jun 25;14:14590. doi: 10.1038/s41598-024-64386-w (PMC11199521; doi:10.1038/s41598-024-64386-w)
Supplement: Supplementary file 1 — Supplementary Information. [file 41598_2024_64386_MOESM1_ESM.zip › Sup data/Summary of supplementary data2.docx]

Table 1: Summary of Collected 840 RC deep beams database.

| **Ref.** | **#** | ***a/d*** | ***f_c_′* (MPa)** | ***ρ_l_* (%)** | ***f_y_* (MPa)** | ***ρ_v_* (%)** | ***f_yv_* (MPa)** | ***ρ_h_* (%)** | ***f_yh_* (MPa)** | **v_n_ =V_u_/b_w_h (MPa)** |
| --- | --- | --- | --- | --- | --- | --- | --- | --- | --- | --- |
| [1] | 55 | 1.16-2.43 | 14.0-48.0 | 0.98-3.42 | 321-370 | 0.0-1.22 | 0-331 | 0 | 0-331 | 1.08-4.69 |
| [2] | 14 | 1.53-1.53 | 17.0-25.0 | 2.72-4.25 | 302-315 | 0.0-0.95 | 0-326 | 0 | 0 | 2.47-4.68 |
| [3] | 24 | 0.35-1.18 | 19.0-25.0 | 0.52-1.73 | 287-287 | 0.0-2.45 | 0-303 | 0.0-2.45 | 0-303 | 3.8-5.66 |
| [4] | 52 | 1.0-2.08 | 16.0-23.0 | 1.94-1.94 | 431-431 | 0.0-1.25 | 484-484 | 0.0-0.91 | 484-484 | 2.01-5.07 |
| [5] | 8 | 0.42-1.53 | 23.0-33.0 | 0.27-1.15 | 580-580 | 0.2-0.24 | 550-550 | 0.35-0.51 | 550-550 | 2.21-7.5 |
| [6] | 16 | 1.0-1.0 | 13.9-26.4 | 1.08-1.52 | 420-420 | 0 | 0 | 0 | 0 | 1.98-4.37 |
| [7] | 2 | 1.75-1.99 | 89.4-89.4 | 2.8-2.8 | 452-452 | 0.16-0.16 | 569-569 | 0 | 0 | 5.45-6.55 |
| [8] | 24 | 1.5-2.5 | 52.0-73.0 | 3.769-3.77 | 414-414 | 0.24-1.81 | 414-414 | 0 | 0 | 3.16-8.97 |
| [9] | 6 | 0.56-1.13 | 31.0-49.0 | 2.6-2.6 | 520-520 | 0.0-0.12 | 0-240 | 0.0-0.12 | 0-240 | 5.99-12.14 |
| [10] | 7 | 2.0-2.0 | 32.0-91.0 | 2.02-2.02 | 410-410 | 0.34-0.5 | 370-370 | 0 | 0 | 2.66-3.79 |
| [11] | 52 | 0.5-2.0 | 24.0-74.0 | 1.29-1.56 | 414-414 | 0.0-0.37 | 414-414 | 0.0-0.94 | 414-414 | 2.46-10.25 |
| [12] | 12 | 1.1-1.1 | 25.0-32.0 | 1.15-1.28 | 469-522 | 0.0-0.45 | 0-455 | 0 | 0 | 2.79-3.81 |
| [13] | 8 | 0.84-0.85 | 30.7-42.5 | 2.6 | 530 | 0-0.41 | 0-250 | 0-0.89 | 0.0-511 | 4.64-10 |
| [14] | 22 | 0.91-1.67 | 31.0-88.0 | 1.38-3.62 | 364-530 | 0.0-0.22 | 0-448 | 0.0-1.44 | 0-577 | 2.51-9.35 |
| [15] | 5 | 2.5-2.5 | 120.1-120.1 | 1.588-6.699 | 431-472 | 0.43-1.75 | 407-458 | 0 | 0 | 1.32-8.46 |
| [16] | 21 | 0.95-1.96 | 11.3-46.8 | 0.57-3.83 | 483-483 | 0 | 0 | 0 | 0 | 1.05-7.27 |
| [17] | 1 | 1.85-1.85 | 14.9-14.9 | 2.37-2.37 | 328-328 | 0 | 0 | 0 | 0 | 1.34-1.34 |
| [18] | 16 | 1.51-1.51 | 22.0-27.0 | 0.75-3.05 | 267-712 | 0 | 0 | 0 | 0 | 1.94-3.37 |
| [19] | 6 | 1.35-1.38 | 17.9-26.1 | 1.59-1.59 | 483-483 | 0 | 0 | 0 | 0 | 2.71-3.77 |
| [20] | 6 | 2.02-2.22 | 21.0-39.7 | 0.98-3.36 | 303-586 | 0 | 0 | 0 | 0 | 1.58-3.55 |
| [21] | 6 | 1.0-2.0 | 22.3-32.4 | 1.78-2.47 | 426-490 | 0 | 0 | 0 | 0 | 1.47-6.39 |
| [22] | 5 | 0.67-1.34 | 23.3-37.0 | 0.83-1.67 | 463-463 | 0 | 0 | 0 | 0 | 4.24-6.31 |
| [23] | 2 | 0.7-1.01 | 13.7-21.5 | 0.26-0.76 | 320-320 | 0 | 0 | 0 | 0 | 1.31-3.11 |
| [22] | 7 | 0.3-0.88 | 13.1-66.6 | 3.0-6.0 | 364-389 | 0 | 0 | 0 | 0 | 3.54-14.87 |
| [24] | 2 | 1.5 | 23.1-79.5 | 3.34 | 414 | 0 | 0 | 0 | 0 | 2.27-5.38 |
| [25] | 3 | 1.05-1.87 | 26.1-42.4 | 0.95-1.12 | 367-455 | 0 | 0 | 0 | 0 | 1.89-3.49 |
| [26] | 9 | 0.5-1.5 | 15.0-20.6 | 0.66-1.9 | 420-420 | 0 | 0 | 0 | 0 | 1.48-4.42 |
| [27] | 4 | 1.43-2.0 | 19.5-20.3 | 0.84-1.75 | 550-550 | 0 | 0 | 0 | 0 | 1.21-1.5 |
| [28] | 3 | 1.0-1.0 | 35.5-40.8 | 1.69-1.91 | 1004-1026 | 0 | 0 | 0 | 0 | 4.23-5.71 |
| [29] | 19 | 0.53-1.13 | 31.0-79.0 | 0.9-1.0 | 408-408 | 0 | 0 | 0 | 0 | 2.27-11.45 |
| [30] | 4 | 1.68-1.7 | 39.4-44.1 | 2.6-2.6 | 620-620 | 0 | 0 | 0 | 0 | 1.92-4.86 |
| [31] | 3 | 1.93-1.93 | 38.0-51.0 | 0.44-0.72 | 468-865 | 0 | 0 | 0 | 0 | 1.64-1.87 |
| [32] | 10 | 2.3-2.3 | 54.0-98.0 | 1.82-3.24 | 500-500 | 0 | 0 | 0 | 0 | 1.87-4.74 |
| [33] | 4 | 1.14-1.27 | 28.0-32.0 | 1.25-1.4 | 420-420 | 0.1-0.31 | 450-450 | 0.0-0.35 | 450-450 | 4.06-4.86 |
| [34] | 6 | 1.5-2.0 | 22.0-24.0 | 0.54-0.54 | 441-441 | 0.11-0.33 | 420-420 | 0.11-0.33 | 420-420 | 1.98-3.11 |
| [35] | 13 | 1.08-1.08 | 58.0-65.0 | 1.32-1.66 | 585-585 | 0.0-0.82 | 397-397 | 0.0-0.4 | 397-397 | 5.78-9.38 |
| [36] | 31 | 1.2-2.5 | 19.0-37.0 | 2.29-2.93 | 441-503 | 0.0-0.86 | 0-558 | 0.0-0.45 | 0-503 | 2.23-8.46 |
| [37] | 12 | 1.05-2.01 | 27.0-36.0 | 0.4-0.85 | 492-492 | 0.3-0.3 | 605-605 | 0.3-0.3 | 605-605 | 2.41-3.77 |
| [38] | 8 | 0.89-0.89 | 27.0-43.0 | 1.8-1.8 | 541-541 | 0 | 0 | 0 | 0 | 4.82-7.31 |
| [39] | 11 | 1.56-2.08 | 18.0-32.0 | 2.11-2.38 | 454-482 | 0.0-0.3 | 0-421 | 0.0-0.37 | 0-421 | 2.21-4.0 |
| [40] | 12 | 1.0-1.0 | 26.0-70.0 | 1.4-1.47 | 546-569 | 0 | 0 | 0 | 0 | 3.13-7.43 |
| [41] | 19 | 0.5-1.5 | 29.0-86.0 | 4.34-4.34 | 552-552 | 0.0-1.01 | 552-552 | 0.0-1.27 | 552-552 | 4.1-16.65 |
| [42] | 10 | 0.76-1.88 | 77.0-120.0 | 1.34-2.41 | 400-400 | 0.67-1.0 | 427-427 | 0.0-0.37 | 427-427 | 5.9-9.52 |
| [43] | 9 | 1.18-2.39 | 23.0-48.0 | 0.52-2.29 | 401-880 | 0.0-0.33 | 0-405 | 0.0-0.45 | 0-855 | 2.96-7.53 |
| [44] | 9 | 0.53-2.0 | 44.0-54.0 | 1.04-2.18 | 578-589 | 0 | 0 | 0 | 0 | 2.55-11.5 |
| [45] | 6 | 1.0-1.0 | 22.0-28.0 | 0.4-0.54 | 528-528 | 0.11-0.33 | 405-405 | 0.11-0.33 | 405-405 | 2.17-3.8 |
| [46] | 4 | 1.56-1.78 | 27.0-34.0 | 1.89-2.16 | 498-498 | 0.5-0.5 | 529-529 | 0 | 529-529 | 4.2-4.84 |
| [47] | 16 | 0.61-0.83 | 35.0-68.0 | 1.69-1.99 | 439-439 | 0.0-0.57 | 0-463 | 0.0-0.63 | 0-463 | 5.7-11.87 |
| [48] | 7 | 1.55-2.28 | 29.0-38.0 | 0.69-0.69 | 652-652 | 0.0-0.1 | 0-490 | 0 | 0 | 0.87-2.42 |
| [49] | 3 | 1.2-1.2 | 38.0-91.0 | 7.35-11.33 | 457-457 | 0 | 0 | 0 | 0 | 9.99-15.47 |
| [50] | 12 | 0.82-1.57 | 22.0-50.0 | 2.05-4.07 | 427-462 | 0.0-0.75 | 407-407 | 0.0-0.17 | 407-407 | 2.84-10.52 |
| [51] | 7 | 1.18-1.26 | 68.0-80.0 | 3.32-3.32 | 580-580 | 0.0-0.45 | 550-550 | 0 | 550-550 | 5.42-10.47 |
| [52] | 11 | 0.44-0.44 | 36.0-45.0 | 1.13-1.13 | 400-400 | 0.0-0.66 | 0-440 | 0.0-0.48 | 0-440 | 6.73-8.24 |
| [53] | 19 | 0.5-1.5 | 23.0-38.0 | 1.99-2.11 | 372-402 | 0.0-0.8 | 372-402 | 0 | 0 | 2.36-8.56 |
| [4] | 4 | 0.77-1.34 | 20.5-21.7 | 1.93-1.93 | 431-431 | 0 | 0 | 0 | 0 | 3.19-4.39 |
| [54] | 10 | 2.5-2.5 | 39.0-80.0 | 2.23-3.51 | 495-495 | 0.09-0.19 | 820-820 | 0 | 0 | 3.89-5.08 |
| [55] | 5 | 1.37-2.09 | 24.0-28.0 | 0.28-0.56 | 469-490 | 0.29-0.44 | 346-429 | 0 | 346-429 | 1.76-3.03 |
| [56] | 18 | 0.94-2.0 | 55.0-58.0 | 0.73-1.83 | 569-569 | 0 | 0 | 0 | 0 | 1.61-7.65 |
| [57] | 18 | 0.27-2.16 | 41.0-59.0 | 1.23-1.23 | 505-505 | 0.48-0.48 | 375-375 | 0 | 0 | 2.73-12.27 |
| [58] | 11 | 0.85-1.69 | 63.0-80.0 | 2.58-2.58 | 499-499 | 0.0-2.86 | 0-447 | 0.0-3.17 | 0-447 | 6.09-14.09 |
| [59] | 10 | 0.28-1.67 | 65.0-72.0 | 2.0-4.08 | 499-538 | 0.48-0.48 | 353-385 | 0 | 0 | 4.55-16.82 |
| [60] | 49 | 0.5-2.5 | 21.0-98.0 | 0.4-2.14 | 458-1330 | 0.0-0.95 | 0-1051 | 0 | 0 | 2.1-14.5 |
| [61] | 9 | 1.25-1.75 | 21.0-24.0 | 0.65-0.65 | 357-357 | 0.06-0.42 | 248-248 | 0.1-0.54 | 248-248 | 1.8-2.85 |
| [6] | 21 | 0.97-1.01 | 14.0-26.0 | 0.78-1.13 | 420-420 | 0.0-0.35 | 0-420 | 0 | 0-420 | 1.82-4.37 |
| [62] | 8 | 0.3-0.9 | 48.0-48.0 | 0.76-1.43 | 630-670 | 0.0-0.5 | 457-457 | 0.34-0.34 | 457-457 | 6.25-9.17 |
| [63] | 14 | 0.57-2.28 | 38.0-41.0 | 1.24-1.43 | 484-495 | 0.35-0.42 | 328-369 | 0.0 | 0-369 | 2.76-6.93 |

# is the number of tests for each reference.

**References**

[1] A. P. Clark, “Diagonal Tension in Reinforced Concrete Beams,” *ACI J. Proc.*, vol. 48, no. 10, doi: 10.14359/11876.

[2] I. M. V. K. G. Moody R. C. Elstner, and E. Hognestad, “Shear Strength of Reinforced Concrete Beams Part 1 -Tests of Simple Beams,” *ACI J. Proc.*, vol. 51, no. 12, doi: 10.14359/11680.

[3] P. J. R. Fung-Kew Kong David F. Cole, “Web Reinforcement Effects on Deep Beams,” *ACI J. Proc.*, vol. 67, no. 12, doi: 10.14359/7336.

[4] K. N. S. and A. S. Vantsiotis, “Shear Strength of Deep Beams,” *ACI J. Proc.*, vol. 79, no. 3, doi: 10.14359/10899.

[5] N. K. Subedi, A. E. Vardy, and N. Kubotat, “Reinforced concrete deep beams some test results,” *Mag. Concr. Res.*, vol. 38, no. 137, pp. 206–219, 1986.

[6] J. W. and N. Lehwalter, “Size Effects in Short Beams Loaded in Shear,” *ACI Struct. J.*, vol. 91, no. 5, doi: 10.14359/4177.

[7] P. Y. L. K. and B. V. Rangan, “Shear Strength of High-Performance Concrete Beams,” *ACI Struct. J.*, vol. 95, no. 6, doi: 10.14359/581.

[8] K.-S. L. Sung-Woo Shin Jung-Ill Moon, and S. K. Ghosh, “Shear Strength of Reinforced High-Strength Concrete Beams with Shear Span-to-Depth Ratios between 1.5 and 2.5,” *ACI Struct. J.*, vol. 96, no. 4, doi: 10.14359/691.

[9] K. H. T. and H. Y. Lu, “Shear Behavior of Large Reinforced Concrete Deep Beams and Code Comparisons,” *ACI Struct. J.*, vol. 96, no. 5, doi: 10.14359/738.

[10] R. S. Pendyala and P. Mendis, “Experimental study on shear strength of high-strength concrete beams,” *Struct. J.*, vol. 97, no. 4, pp. 564–571, 2000.

[11] J.-K. O. and S.-W. Shin, “Shear Strength of Reinforced High-Strength Concrete Deep Beams,” *ACI Struct. J.*, vol. 98, no. 2, doi: 10.14359/10184.

[12] N. Zhang and K.-H. Tan, “Size effect in RC deep beams: Experimental investigation and STM verification,” *Eng. Struct.*, vol. 29, no. 12, pp. 3241–3254, 2007.

[13] K.-H. Tan, G.-H. Cheng, and N. Zhang, “Experiment to mitigate size effect on deep beams,” *Mag. Concr. Res.*, vol. 60, no. 10, pp. 709–723, 2008.

[14] K. S. Ismail, “Shear behaviour of reinforced concrete deep beams,” University of Sheffield, 2016.

[15] J. J. Roller and H. G. Russel, “Shear strength of high-strength concrete beams with web reinforcement,” *Struct. J.*, vol. 87, no. 2, pp. 191–198, 1990.

[16] J. M. and I. M. Viest, “Shear Strength of Reinforced Concrete Frame Members Without Web Reinforcement,” *ACI J. Proc.*, vol. 53, no. 3, doi: 10.14359/11558.

[17] T. S. Chang and C. E. Kesler, “Static and fatigue strength in shear of beams with tensile reinforcement,” in *Journal Proceedings*, 1958, vol. 54, no. 6, pp. 1033–1057.

[18] D. Watstein and R. G. Mathey, “Strains in beams having diagonal cracks,” in *Journal Proceedings*, 1958, vol. 55, no. 12, pp. 717–728.

[19] J. J. Rodriguez, A. C. Bianchini, I. M. Viest, and C. E. Kesler, “Shear strength of two-span continous reinforced concrete beams,” in *Journal Proceedings*, 1959, vol. 55, no. 4, pp. 1089–1130.

[20] R. D. De Cossio and C. P. Siess, “Behavior and strength in shear of beams and frames without web reinforcement,” in *Journal Proceedings*, 1960, vol. 56, no. 2, pp. 695–736.

[21] F. Leonhardt and R. Walther, “The Stuttgart shear tests,” *Cem. \& Concr. Assoc. Libr.*, vol. 11, no. 28, pp. 49–54, 1961.

[22] Q. Q. Liang, B. Uy, M. A. Bradford, and H. R. Ronagh, “Strength Analysis of Steel–Concrete Composite Beams in Combined Bending and Shear,” *J. Struct. Eng.*, vol. 131, no. 10, pp. 1593–1600, 2005, doi: 10.1061/(asce)0733-9445(2005)131:10(1593).

[23] V. Ramakrishnan and Y. Ananthanarayana, “Ultimate strength of deep beams in shear,” in *Journal Proceedings*, 1968, vol. 65, no. 2, pp. 87–98.

[24] A. G. Mphonde and G. C. Frantz, “Shear tests of high-and low-strength concrete beams without stirrups,” in *Journal Proceedings*, 1984, vol. 81, no. 4, pp. 350–357.

[25] J. G. M. David M. Rogowsky and See Y. Ong, “Tests of Reinforced Concrete Deep Beams,” *ACI J. Proc.*, vol. 83, no. 4, doi: 10.14359/10558.

[26] N. Lehwalter, “Bearing capacity of concrete compression struts in truss-systems, exemplified by the case of short beams,” 1988.

[27] P. Adebar, “One-way shear strength of large footings,” *Can. J. Civ. Eng.*, vol. 27, no. 3, pp. 553–562, 2000, doi: 10.1139/l00-008.

[28] T. LERTSRISAKULRAT, “Concept of Concrete Compressive Fracture Energy in RC Deep Beams without Transverse Reinforcement,” コンクリート工学年次論文集, vol. 23, no. 3, pp. 97–102, 2001.

[29] K.-H. Yang, H.-S. Chung, E.-T. Lee, and H.-C. Eun, “Shear characteristics of high-strength concrete deep beams without shear reinforcements,” *Eng. Struct.*, vol. 25, no. 10, pp. 1343–1352, 2003.

[30] K. H. Tan, G. H. Cheng, and H. K. Cheong, “Size effect in shear strength of large beams—Behaviour and finite element modelling,” *Mag. Concr. Res.*, vol. 57, no. 8, pp. 497–509, 2005.

[31] H. Seliem, A. Hosny, H. Dwairi, and S. Rizkalla, “Shear behavior of concrete beams reinforced with MMFX steel without web reinforcement,” *NC State Univ. Final Report, Proj. No. IS-06-08*, 2006.

[32] E. Thorenfeldt and G. Drangsholt, “Shear capacity of reinforced high-strength concrete beams,” *Spec. Publ.*, vol. 121, pp. 129–154, 1990.

[33] A. B. M. Gerardo Aguilar Gustavo J. Parra-Montesinos, Julio A. Ramirez, and James K. Wight, “Experimental Evaluation of Design Procedures for Shear Strength of Deep Reinforced Concrete Beams,” *ACI Struct. J.*, vol. 99, no. 4, doi: 10.14359/12123.

[34] P. Amornpinyo, “Influence of horizontal to vertical reinforcement ratio on behavior of reinforced concrete deep beams designed by strut–and–tie method,” Khon Kaen University, 2010.

[35] A. Arabzadeh, R. Aghayari, and A. Rahai, “Investigation of experimental and analytical shear strength of reinforced concrete deep beams,” *Int. J. Civ. Eng.*, vol. 9, pp. 207–214, 2011.

[36] D. Birrcher, R. Tuchscherer, M. Huizinga, S. Wood, and J. Jirsa, “Strength and Serviceability Design of Reinforced Concrete Inverted-T Beams,” vol. 7, p. 400, 2013.

[37] S. Brena and N. C. Roy, “Evaluation of load transfer and strut strength of deep beams with short longitudinal bar anchorages,” *ACI Struct. J.*, vol. 106, pp. 678–689, 2009.

[38] Y. Li, H. Chen, W.-J. Yi, F. Peng, Z. Li, and Y. Zhou, “Effect of member depth and concrete strength on shear strength of RC deep beams without transverse reinforcement,” *Eng. Struct.*, vol. 241, p. 112427, 2021, doi: https://doi.org/10.1016/j.engstruct.2021.112427.

[39] A. Demir, N. Caglar, and H. Ozturk, “Parameters affecting diagonal cracking behavior of reinforced concrete deep beams,” *Eng. Struct.*, vol. 184, pp. 217–231, 2019, doi: https://doi.org/10.1016/j.engstruct.2019.01.090.

[40] A. K. El-Sayed and A. B. Shuraim, “Size effect on shear resistance of high strength concrete deep beams,” *Mater. Struct.*, vol. 49, no. 5, pp. 1871–1882, 2016, doi: 10.1617/s11527-015-0619-1.

[41] I. Fang, J. Chen, and L. Hong, “Shear behavior of high strength concrete deep beams,” 1995.

[42] S. J. F. and R. I. Gilbert, “Experimental Studies on High-Strength Concrete Deep Beams,” *ACI Struct. J.*, vol. 95, no. 4, doi: 10.14359/554.

[43] J. de D. G.-M. and A. S. Lubell, “Behavior of Deep Beams Containing High-Strength Longitudinal Reinforcement,” *ACI Struct. J.*, vol. 113, no. 1, doi: 10.14359/51687910.

[44] R. K. and G. A. Rao, “Experimental Verification of ACI 318 Strut-and-Tie Method for Design of Deep Beams without Web Reinforcement,” *ACI Struct. J.*, vol. 118, no. 1, doi: 10.14359/51728083.

[45] S. Kunopas, “Influence of horizontal to vertical reinforcement ratio on behavior of reinforced concrete deep beams designed by strut–and–tie method,” Khon Kaen University, Thailand, 2008.

[46] D. Lee, “An experimental investigation in the effects of detailing on the shear behaviour of deep beams,” University of Toronto, 1982.

[47] I.-J. L. Wen-Yao Lu and Hsin-Wan Yu, “Shear Strength of Reinforced Concrete Deep Beams,” *ACI Struct. J.*, vol. 110, no. 4, doi: 10.14359/51685752.

[48] E. C. B. Boyan I. Mihaylov and Michael P. Collins, “Behavior of Large Deep Beams Subjected to Monotonic and Reversed Cyclic Shear,” *ACI Struct. J.*, vol. 107, no. 6, doi: 10.14359/51664021.

[49] G. T. Proestos, D. K. Palipana, and B. I. Mihaylov, “Evaluating the shear resistance of deep beams loaded or supported by wide elements,” *Eng. Struct.*, vol. 226, p. 111368, 2021, doi: https://doi.org/10.1016/j.engstruct.2020.111368.

[50] G. P.-M. Carlos G. Quintero-Febres and James K. Wight, “Strength of Struts in Deep Concrete Members Designed Using Strut-and-Tie Method,” *ACI Struct. J.*, vol. 103, no. 4, doi: 10.14359/16434.

[51] R. Vollum and J. Sagaseta, “Shear design of short-span beams,” *Mag. Concr. Res. - MAG CONCR RES*, vol. 62, pp. 267–282, 2010, doi: 10.1680/macr.2010.62.4.267.

[52] D. K. Sahoo, M. S. V. Sagi, B. Singh, and P. Bhargava, “Effect of Detailing of Web Reinforcement on the Behavior of Bottle-shaped Struts,” *J. Adv. Concr. Technol.*, vol. 8, no. 3, pp. 303–314, 2010, doi: 10.3151/jact.8.303.

[53] M. Salamy, H. Kobayashi, S. Unjoh, K. Kosa, and T. Nishioka, “A comparative study on RC deep beams behavior with shear span to depth ratio between 0.5 and 1.5,” 2005.

[54] K. F. S. and J. M. S. Al-Musawi, “Shear Design of High- and Normal Strength Concrete Beams with Web Reinforcement,” *ACI Struct. J.*, vol. 89, no. 6, doi: 10.14359/9644.

[55] A. Senturk and C. Higgins, “Evaluation of Reinforced Concrete Deck Girder Bridge Bent Caps with 1950s Vintage Details: Analytical Methods,” *ACI Struct. J.*, vol. 107, pp. 544–553, 2010.

[56] A. B. Shuraim and A. K. El-Sayed, “Experimental verification of strut and tie model for HSC deep beams without shear reinforcement,” *Eng. Struct.*, vol. 117, pp. 71–85, 2016, doi: https://doi.org/10.1016/j.engstruct.2016.03.002.

[57] F.-K. K. Kang-Hai Tan Susanto Teng, and LingweiI Guan, “High-Strength Concrete Deep Beams With Effective Span and Shear Span Variations,” *ACI Struct. J.*, vol. 92, no. 4, doi: 10.14359/991.

[58] F.-K. K. Kang-Hai Tan Susanto Teng, and Li-Wei Weng, “Effect of Web Reinforcement on High-Strength Concrete Deep Beams,” *ACI Struct. J.*, vol. 94, no. 5, doi: 10.14359/506.

[59] S. T. Kang-Hai Tan Fung-Kew Kong, and Hai-Yun Lu, “Main Tension Steel in High Strength Concrete Deep and Short Beams,” *ACI Struct. J.*, vol. 94, no. 6, doi: 10.14359/9735.

[60] Y. TANIMURA and T. SATO, “Evaluation of Shear Strength of Deep Beams with Stirrups,” *Q. Rep. RTRI*, vol. 46, no. 1, pp. 53–58, 2005, doi: 10.2219/rtriqr.46.53.

[61] P. Tasenhod, “Behaviors of reinforced concrete deep beams,” Khon Kaen University, Thailand, 2014.

[62] J.-H. Zhang, S.-S. Li, W. Xie, and Y.-D. Guo, “Experimental Study on Shear Capacity of High Strength Reinforcement Concrete Deep Beams with Small Shear Span–Depth Ratio,” *Materials (Basel).*, vol. 13, no. 5, 2020, doi: 10.3390/ma13051218.

[63] Z. Ning, T. Kang-Hai, and L. Chee-Lai, “Single-Span Deep Beams Subjected to Unsymmetrical Loads,” *J. Struct. Eng.*, vol. 135, no. 3, pp. 239–252, Mar. 2009, doi: 10.1061/(ASCE)0733-9445(2009)135:3(239).
