# Supplementary figures and images for: Prediction and reliability analysis of shear strength of RC deep beams
Source: Sci Rep. 2024 Jun 25;14:14590. doi: 10.1038/s41598-024-64386-w (PMC11199521; doi:10.1038/s41598-024-64386-w)

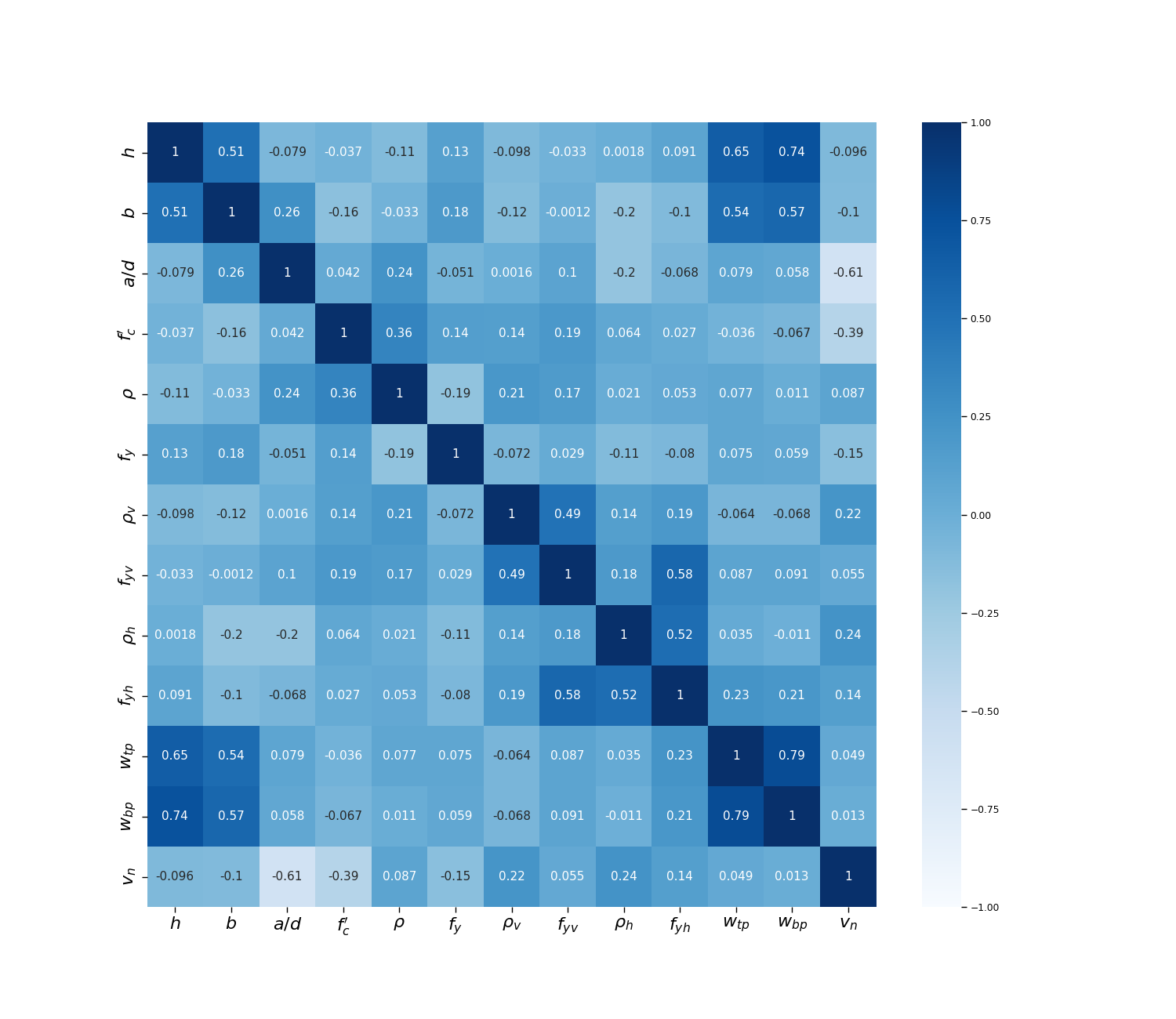

Supplement: Supplementary file 1 — Supplementary Information. [file 41598_2024_64386_MOESM1_ESM.zip › Sup data/Drawings/correlation_shear.png]

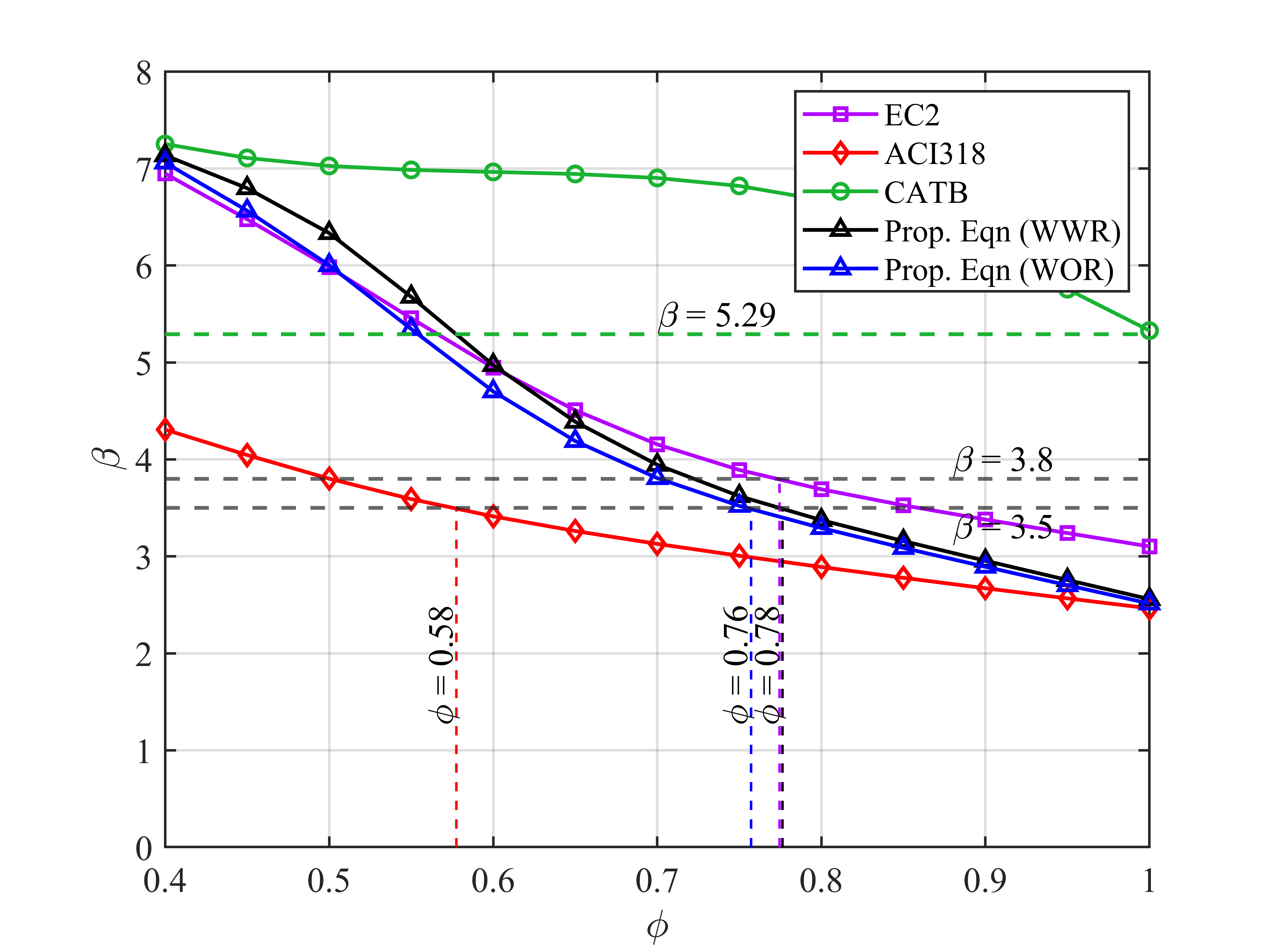

Supplement: Supplementary file 1 — Supplementary Information. [file 41598_2024_64386_MOESM1_ESM.zip › Sup data/Drawings/reliabilty.png]

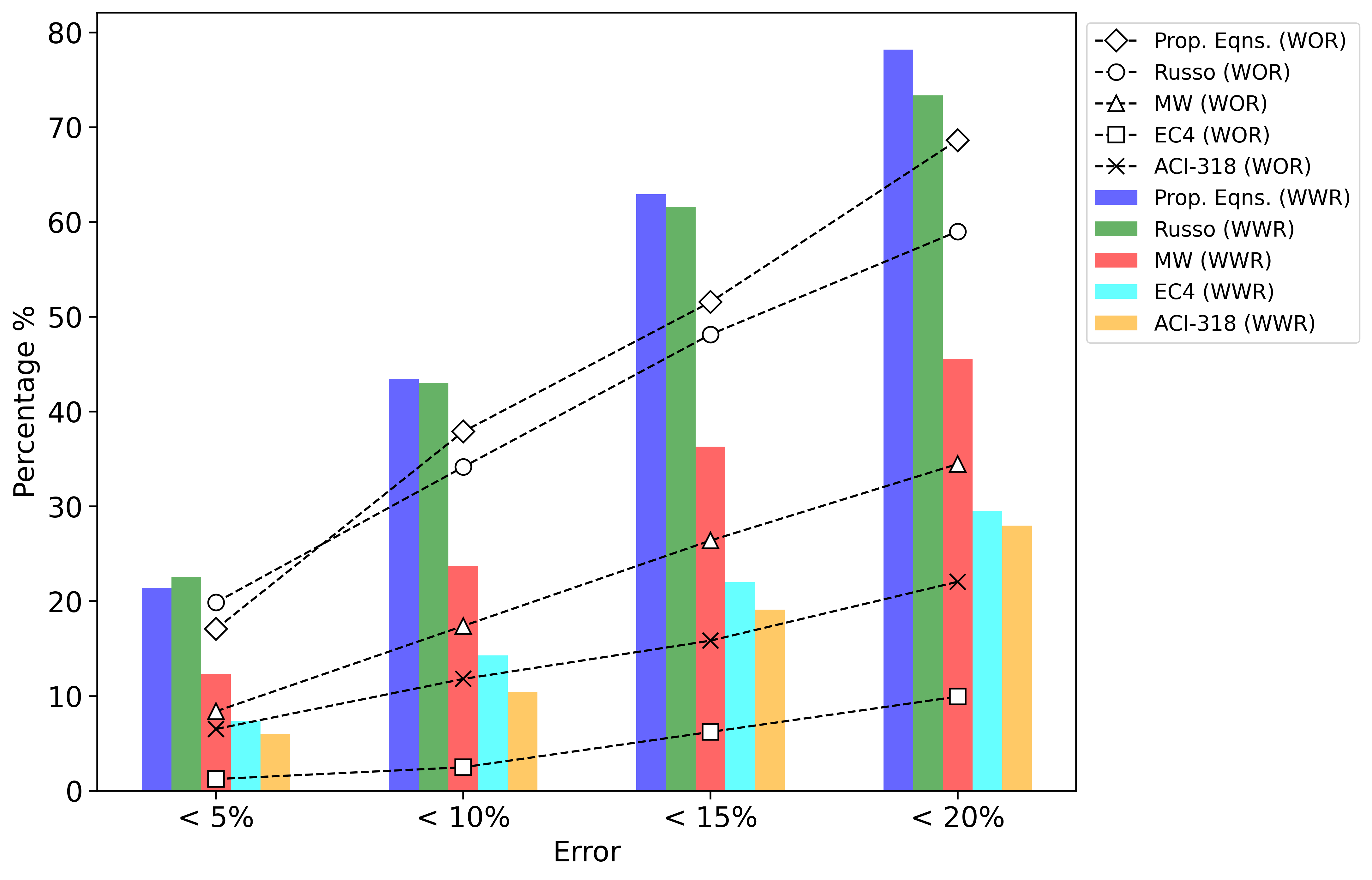

Supplement: Supplementary file 1 — Supplementary Information. [file 41598_2024_64386_MOESM1_ESM.zip › Sup data/Drawings/Shear222.png]

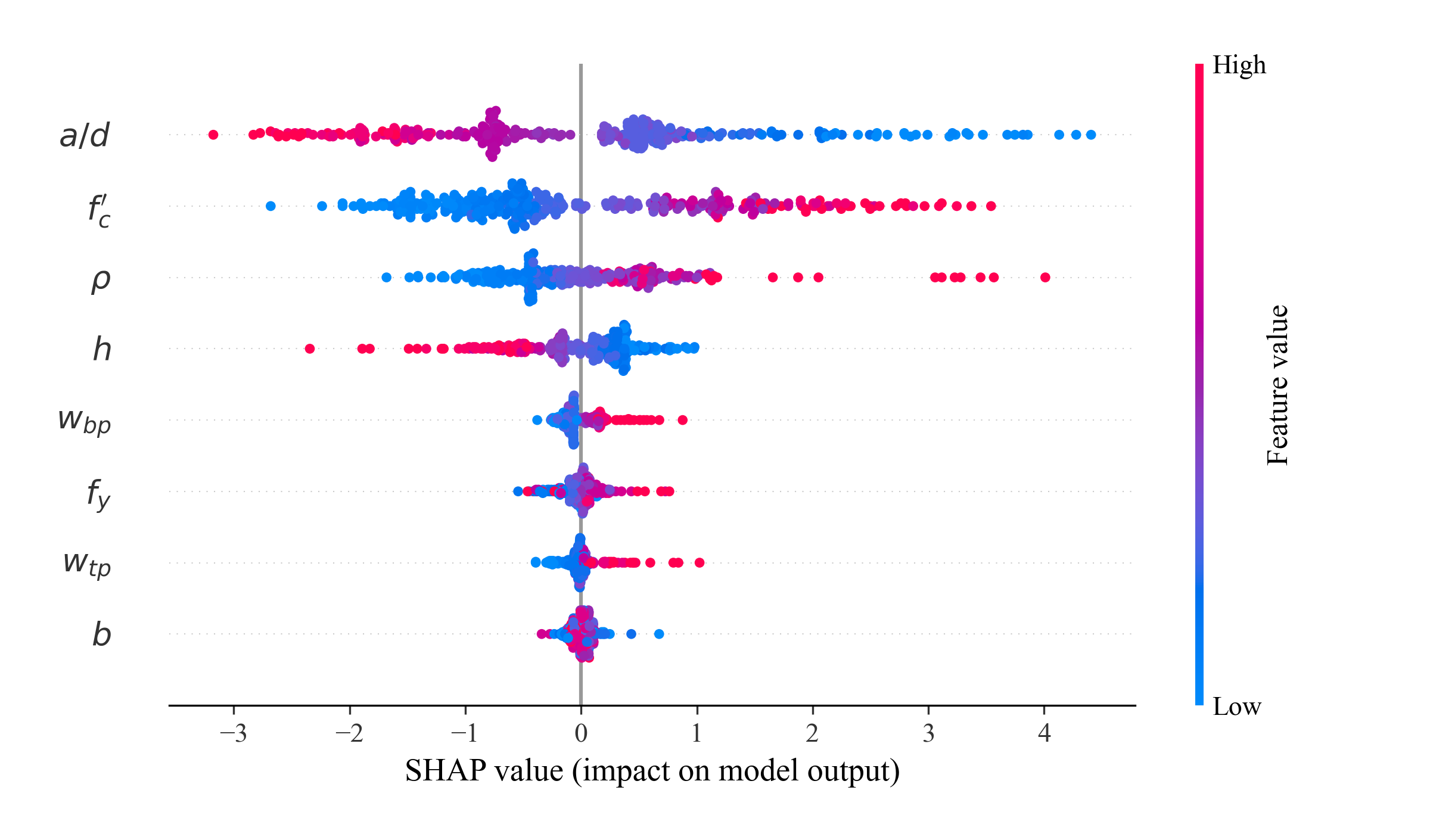

Supplement: Supplementary file 1 — Supplementary Information. [file 41598_2024_64386_MOESM1_ESM.zip › Sup data/Drawings/summary_plot_shear.png]

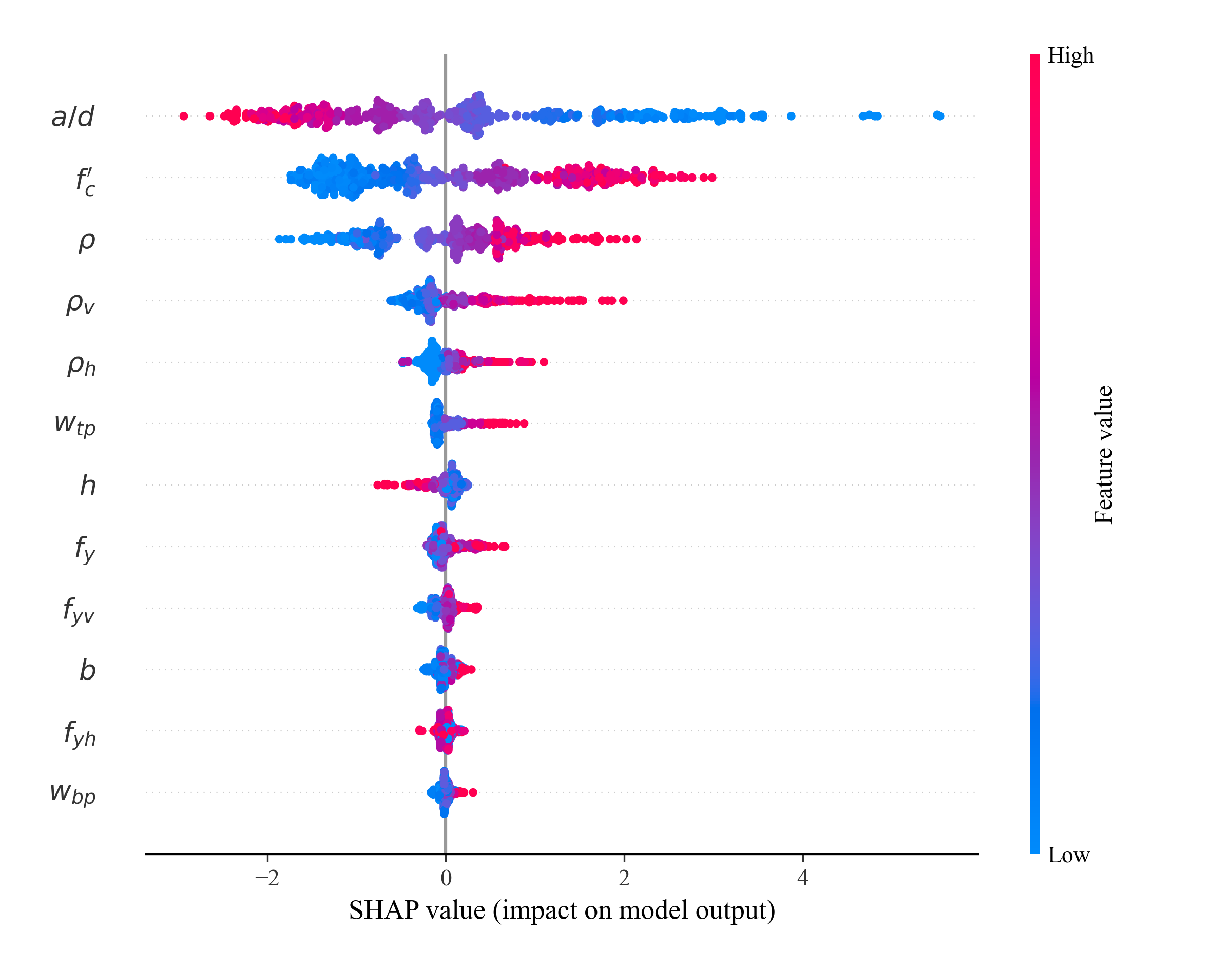

Supplement: Supplementary file 1 — Supplementary Information. [file 41598_2024_64386_MOESM1_ESM.zip › Sup data/Drawings/summary_plot_shear2.png]

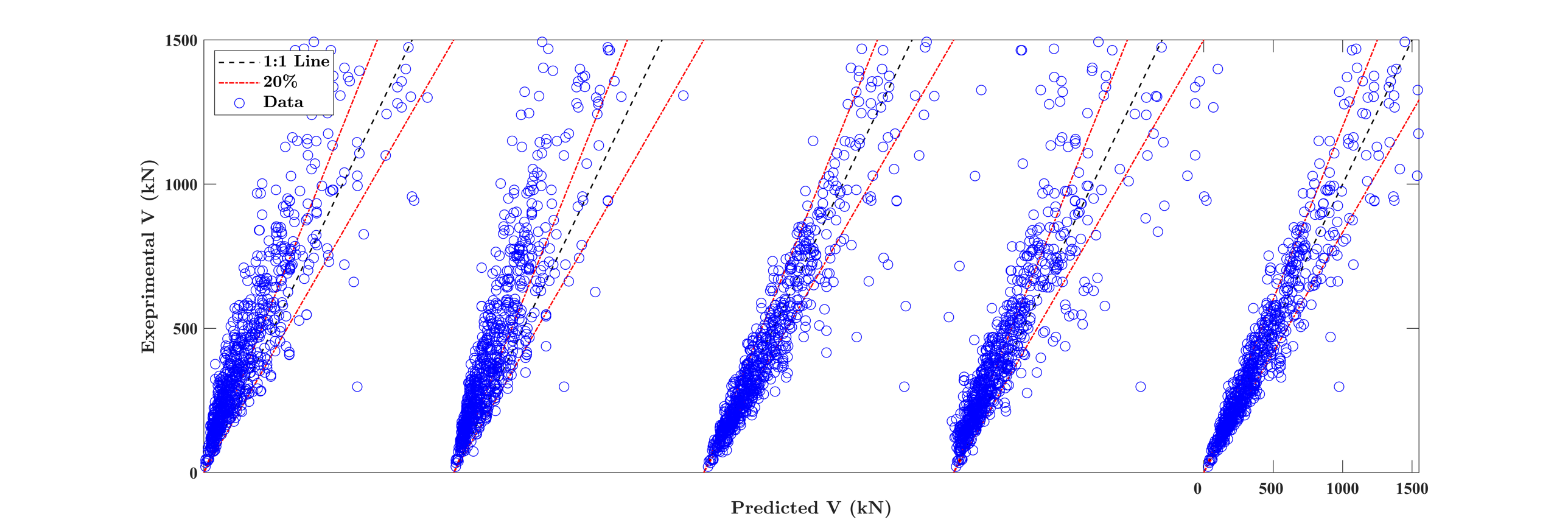

Supplement: Supplementary file 1 — Supplementary Information. [file 41598_2024_64386_MOESM1_ESM.zip › Sup data/Drawings/train_test_Codes0.png]

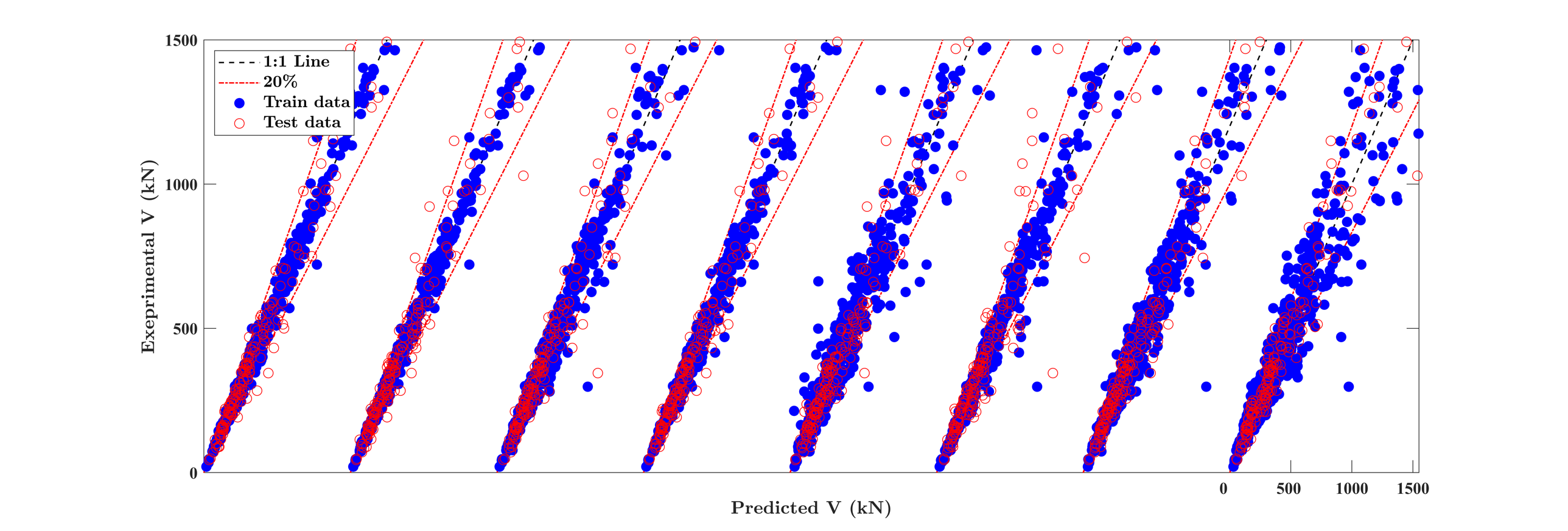

Supplement: Supplementary file 1 — Supplementary Information. [file 41598_2024_64386_MOESM1_ESM.zip › Sup data/Drawings/train_test_ML.png]
